# Kingdom-wide analysis of the evolution of the plant type III polyketide synthase superfamily

**DOI:** 10.1093/plphys/kiaa086

**Published:** 2020-12-30

**Authors:** Thomas Naake, Hiroshi A Maeda, Sebastian Proost, Takayuki Tohge, Alisdair R Fernie

**Affiliations:** 1 Max Planck Institute of Molecular Plant Physiology, Am Mühlenberg 1, 14476 Potsdam, Germany; 2 Department of Botany, University of Wisconsin–Madison, 430 Lincoln Drive, Madison, WI 53706, USA; 3 Laboratory of Molecular Bacteriology, Department of Microbiology and Immunology, Rega Institute, KU Leuven, Herestraat, 3000 Leuven, Belgium; 4 VIB-KU Leuven Center for Microbiology, Campus Gasthuisberg, Rega Instituut, Herestraat, 3000 Leuven, Belgium; 5 Nara Institute of Science and Technology, 8916-5 Takayama-cho, Ikoma, Nara 630-0192, Japan

## Abstract

The emergence of type III polyketide synthases (PKSs) was a prerequisite for the conquest of land by the green lineage. Within the PKS superfamily, chalcone synthases (CHSs) provide the entry point reaction to the flavonoid pathway, while LESS ADHESIVE POLLEN 5 and 6 (LAP5/6) provide constituents of the outer exine pollen wall. To study the deep evolutionary history of this key family, we conducted phylogenomic synteny network and phylogenetic analyses of whole-genome data from 126 species spanning the green lineage including *Arabidopsis thaliana*, tomato (*Solanum lycopersicum*), and maize (*Zea mays*). This study thereby combined study of genomic location and context with changes in gene sequences. We found that the two major clades, CHS and LAP5/6 homologs, evolved early by a segmental duplication event prior to the divergence of Bryophytes and Tracheophytes. We propose that the macroevolution of the type III PKS superfamily is governed by whole-genome duplications and triplications. The combined phylogenetic and synteny analyses in this study provide insights into changes in the genomic location and context that are retained for a longer time scale with more recent functional divergence captured by gene sequence alterations.

## Teaser

Phylogenetic and syntenic analyses of whole genome data reveal that macroevolution of the type III polyketide synthase superfamily is mainly governed by whole-genome duplications and triplications.

## Introduction

During plant evolution, the number of specialized metabolites and the enzymes responsible for their synthesis exploded (Weng et al., 2012; Moghe and Last, 2015). The number of protein folds, however, remained restricted (Chothia and Lesk, 1986; Weng et al., 2012). This is likely because novel biosynthetic pathways generally originate by gene duplication events and/or by functional divergence of existing genes (Moghe and Last, 2015). Commonly, duplicated genes, from already enzymatically active enzymes, were subjected to differential mutations resulting in a broader substrate specificity and a lower activation energy of catalysis, led single enzymes catalyzing multiple reactions, and thereby synthesizing multiple products (Weng et al., 2012).

About 450–500 million years ago, Charophycean freshwater green algae began to colonize land (Kenrick and Crane, 1997; Gensel, 2008; Banks et al., 2011). Early land plants needed to adapt quickly to their altered environment leading to the innovation of novel metabolic pathways, including phenylpropanoids, sporopollenin, and lignin biosynthesis. These include the provision of rigid building blocks to allow for growth on land and molecules for biotic and abiotic stress (Weng and Chapple, 2010; Weng et al., 2012). They achieved this by “recycling” enzymes from existing core pathways, co-opting them, and evolving novel functionalities (Lesburg et al., 1997; Wendt et al., 1997; Bohlmann et al., 1998; Austin and Noel, 2003; Weng, 2014; Moghe and Last, 2015). The availability of plant and algae genomes allowed to trace the diversification of plant enzyme families undergoing evolutionary alterations and shaping the vast plant chemical diversity seen today (Nelson and Werck-Reichhart, 2011; Shockey and Browse, 2011; Caputi et al., 2012; Kawai et al., 2014). These studies, while highly informative, were, however, restricted to phylogenetic analyses of gene/protein sequences and did not take into account the genomic context for all analyzed species which would reveal the deep ancestral history and the points of diversification of a gene family.

Given their strategic importance within the phenylpropanoid pathway, we postulate that type III polyketide synthases (PKSs) may have played a major role in the colonization of land by providing the precursors for the synthesis of flavonoids (Buer and Muday, 2004) and sporopollenin (Dobritsa et al., 2010; Kim et al., 2010). The type III PKS superfamily is a prime example of how the recruitment of an existing pathway led to the diversification of metabolic routes (Austin and Noel, 2003; Morita et al., 2019). PKS enzymes are likely derived from the β-ketoacyl acyl carrier protein (ACP) synthases of fatty acid biosynthesis (Yonekura-Sakakibara et al., 2019), as they share a protein fold (Austin and Noel, 2003; Yonekura-Sakakibara et al., 2019). PKSs, including type III PKS, like their predecessors from fatty acid metabolism, catalyze the sequential head-to-tail condensation of two-carbon acetate units derived from a malonate thioester into a growing linear polyketide chain (Austin and Noel, 2003). All type III PKSs share a common αβαβα structural fold, a conserved catalytic triad consisting of Cys–His–Asn and act as homodimers consisting of ∼40 kDa monomeric subunits. By contrast, type I PKSs which act in a modular or iterative fashion are multi-domain protein complexes, typically consisting of a large subunit of tandemly arranged domains with ketosynthases (KS), acyltransferases (AT), and ACPs representing their essential domains (Austin and Noel, 2003). Type II PKSs are multi-domain protein complexes consisting of discrete, separable proteins composed of a heterodimeric KS (KS and chain initiation factor subunits) and an ACP, but lacking the AT domain present in type I PKSs.

**Figure 1:**
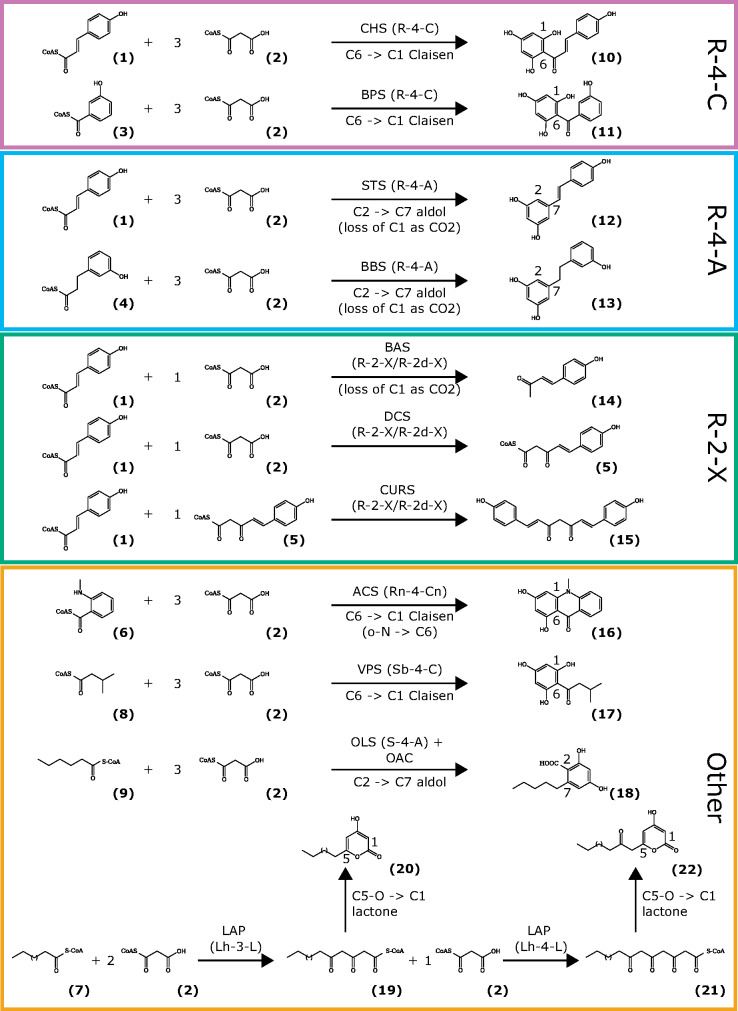
Overview of important reactions catalyzed by type III PKSs. The reaction type is defined on the basis of combinations of three features according to pPAP (Shimizu et al., 2017a, 2017b): 1) starter substrate: three categories for the starter substrates based on their acyl group: ring (R), short chain (S, C2–C12), or long chain (L, up to C26). Additional characters are added to specify acylgroups (branched chain, b; carboxylate group, c; hydroxy group, h; nitrogen, n); 2) number of condensations: indicates the number of methylenecarbonyl units in the intermediates. Additional characters are added to specify other substrates than malonyl-CoA (methylmalonyl-CoA, m; ethylmalonyl-CoA, e; acetoacetyl-CoA, a; diketide-CoA, d); 3) mechanism of intramolecular cyclization: Claisen, C; aldol, A; lactone, L; no cyclization, X; nitrogen-carbon, n. The features 1) and 2) are interrelated since Claisen- or aldol-type cyclizations require typically at least four and lactonization at least three carbonyl units in the intermediate. The pPAP software will define four major categories for a classified reaction type (R-4-C for CHS and BPS; R-4-A for STS and BBS; R-2-X for BAS, DCS, and CURS; Other for ACS, VPS, OLS, and LAP, see colored boxes). (1) *p*-coumaroyl CoA, (2) malonyl CoA, (3) 3-hydroxybenzoyl CoA, (4) dihydro-*m*-coumaroyl CoA, (5) p-coumaroyl diketide CoA, (6) *N*-methylanthraniloyl CoA, (7) fatty acid acyl CoA, (8) isovaleroyl CoA, (9) hexanoyl CoA, (10) naringenin chalcone, (11) 2,3',4,6-tetrahydroxybenzophenone, (12) resveratrol, (13) 3,3',5-trihydroxybibenzyl, (14) benzalacetone, (15) bisdemethoxycurcumin, (16) 1,3-dihydroxy-*N*-methylacridone, (17) phloroisovalerophenone, (18) olivetolic acid, (19) triketide intermediate, (20) triketide pyrone, (21) tetraketide intermediate, (22) tetraketide pyrone. ACS, acridone synthase; BAS, benzalacetone synthase; BBS, bibenzyl synthase; BPS, benzophenone synthase; CURS, curcumin synthase; DCS, diketide-CoA synthase; LAP, hydroxyalkyl α-pyrone synthase/LESS ADHESIVE POLLEN; OLS, 3,5,7-trioxododecanoyl-CoA synthase/olivetol synthase; PKS, polyketide synthase; VPS, phloroisovalerophenone synthase.

Type I and type II PKSs are present in bacteria (Austin and Noel, 2003) and type I PKSs are found in fungi (Austin and Noel, 2003) and algae (Shelest et al., 2015), and ubiquitously present in plants (Austin and Noel, 2003). Type III PKSs are widely distributed in bacteria (Funa et al., 2002, 2007), fungi (Funa et al., 2007; Hashimoto et al., 2014), and ubiquitously present in plants (Austin and Noel, 2003). More than 20 functionally different plant type III PKSs have been described (Figure 1) which share 30%–95% sequence identity (Morita et al., 2019). Among the most prominent members are chalcone synthases (CHSs) and LESS ADHESIVE POLLEN 5/6 (LAP5/6) proteins (Morita et al., 2019). CHSs catalyze the entry point of flavonoid metabolism and are well characterized in a number of model species (Wienand et al., 1986; O’Neill et al., 1990; Shirley et al., 1995). LAP5/6 provide building blocks of the pollen exine layer (Dobritsa et al., 2010; Kim et al., 2010). The diverse functions of PKSs arose due to their differences in (1) substrate specificities, (2) the number of condensation rounds, and (3) cyclization reactions all of which are ultimately governed by the sequence of the genes which encode them (Austin and Noel, 2003; Morita et al., 2019).

To elucidate the evolution of type III PKSs, we here utilized a phylogenomic network approach (Zhao et al., 2017; Zhao and Schranz, 2017, 2019), to study the syntenic relationships between genomic regions of a myriad of species spanning the green lineage. Synteny, the conservation of gene content and order within or between genomes, infers a shared evolutionary history. Although, admittedly, synteny may also be lost very quickly by gene translocation, the use of many species comparisons including several members of a given clade severely reduces the chances of this process distorting the correct inference from such analyses. Synteny analysis, therefore, provides a means to examine the ancient history of gene evolution, since gene sequences can change their functionality by mutations, while synteny can be retained over a longer time scale. Such approaches allow the inference of the orthology, timing, and mode of duplication of pairs/groups of genes (Kurata et al., 1994). Here, we modified the approach in order to allow cross-kingdom analysis and combined it with the phylogenetic approach to investigate the relationship between functional divergence of various genes and their genomic location. These combined analyses revealed an early segmental duplication event that led to the emergence of the LAP and CHS clades. We also provide evidence that the evolution of the type III PKS superfamily is governed by whole-genome duplication (WGD) and triplication (WGT) events following the emergence of the LAP and CHS clades. We propose an evolutionary route for the CHSs governed by a WGT event and its subsequent diversification in a Fabales-specific clade. Our combined results are further discussed in the context of early land plant colonization and the maintenance of presence of type III *PKS* genes in their genomic context.

## Results

### PKS copy numbers widely vary among different plant and green algae species

Flavonoid and sporopollenin biosynthesis evolved on the terrestrialization of the green lineage. To study the diversification of the PKS superfamily, the fully sequenced genomes of 126 species spanning the green lineage (Supplemental Figure S1) were queried for the number of *PKS* copies they possessed. To provide a robust classification of protein families, we used OrthoFinder (Emms and Kelly, 2015) and MCL (Enright et al., 2002). We detected 63,344 and 61,643 groups containing more than one protein sequence for OrthoFinder and MCL clustering, respectively. Within this dataset, the type III PKS superfamily formed one protein group with 1,621 different protein sequences detected by either of the clustering methods, of which 1,551 protein signatures/sequences were jointly detected by both. Within these groups, all previously characterized and described PKS sequences were recovered (see Supplemental Dataset S1). Reassuringly, the protein family of β-ketoacyl ACP synthases, which exhibits sequence similarity to PKSs, formed a separate group in both the OrthoFinder and MCL output.

We have to note a bias in the selection of genomes toward angiosperm genomes since the selection of species was driven by availability of high-quality genomes and this is inherently biased toward angiosperms. *PKS* are present in all land plants albeit in varying copy numbers (Supplemental Figure S2 and Supplemental Table S1). However, *PKS* were not found, or only found in low copy numbers, in the Chlorophyta, and are absent in Chlorokybophyceae, Mesostigmaphyceae, and Klebsormidiophyceae of the Charophyta (see Supplemental File S1). By contrast, type III *PKS* were detected in *Penium margaritaceum* (Jiao et al., 2020), a member of the Zygnematophyceae, a more recent lineage of the green algae.

### Synteny network analysis detects clade-specific and reaction type-specific clusters

**Figure 2:**
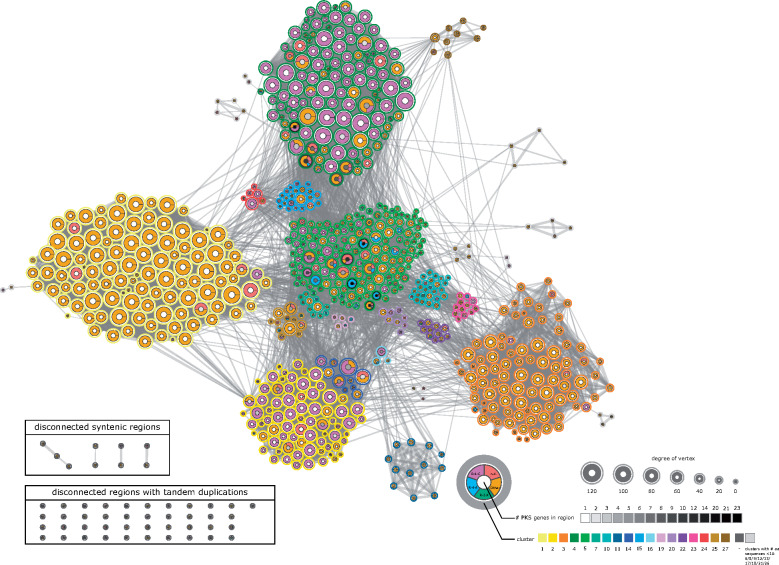
Network showing synteny between regions containing type III *PKS* genes. The network contains four “R-4-C”-enriched syntenic clusters corresponding to CHS function (2, 4, 5, and 14) and four syntenic clusters with *LAP5* and *LAP6* orthologs (1, 3, 11, and 27). The syntenic clusters showed species-specific distribution (Supplemental Figure S3). Each vertex depicts information on the pPAP-classification of *PKS* within the genomic region (refer to Figure 1 for further information), the number of *PKS* within the region (number of tandem duplicates) and the cluster membership. Clusters were detected by applying the community detection algorithms, “fastgreedy,” “walktrap,” “leading eigenvector,” and “multilevel” on the weighted network, followed by affinity propagation clustering. Vertex size reflects degree of the vertex (unweighted number of edges connecting to the vertex). LAP, LESS ADHESIVE POLLEN.

To study the diversification of the type III PKS superfamily, we followed a synteny network approach (Zhao and Schranz, 2017). Whole genomes of 126 species were compared in a pairwise manner, followed by robust block detection of regions containing type III *PKS* genes and network analysis to detect syntenic clusters within the network. Here, network nodes correspond to genomic regions containing one or multiple (tandem-duplicated) *PKS* genes, while edges correspond to detected synteny between these regions based on the results of two commonly employed algorithms for synteny detection, i-ADHoRe and MCScanX (Proost et al., 2012; Wang et al., 2012). The resultant network contained 706 vertices corresponding to syntenic regions containing single or multiple type III *PKS* genes, of which 166 vertices corresponded to regions with tandem-duplicated genes, from a total of 105 species (Supplemental Table S2). Tandem-duplicated genes may play important roles in providing genetic redundancy, gene dosage balance, genetic robustness, and to provide an additional means for divergence in transcriptional regulation and protein sequence (Semon and Wolfe, 2007; Hahn, 2009; Innan and Kondrashov, 2010; Liu et al., 2011). The highest number of tandem-duplicated *PKS* genes in one syntenic region was 23 (*Arachis duranensis*, containing mainly “R-4-A”-type stilbene synthase, STS, sequences, Figures 1, 2). *Arachis ipaensis* (21 genes) and grape vine (*Vitis vinifera*, 20 genes) had the second- and third-highest numbers of tandem-duplicated genes (also containing mostly “R-4-A”-type STS sequences), respectively.

For most of the possible syntenic relationships, the network was characterized by an absence of synteny between *PKS*-containing regions. The network was sparse with 4.7% of all possible edges (11,702 of 248,865 possible edges). We applied a robust cluster detection approach on the network using four different algorithms that resulted in the detection of 27 syntenic clusters (Figure 2 and interactive Figure 1 available at https://pksevolution.github.io/PKS_visualizations/).

The previously characterized dicot *CHS* genes, such as tomato (*Solanum lycopersicum*) *Solyc09g091510/SlCHS1* and *Solyc05g053550/SlCHS2* were located to the syntenic cluster 2, while *Arabidopsis thaliana AT5G13930/TT4* and maize (*Zea mays*) *Zm00001d052673/C2* were found in syntenic cluster 4 (Figure 2). Of the 27 clusters, some were shared between systematic groups, including the major clusters 1, 2, 3, and 5, while others were clade-specific. For instance, cluster 4 with the second highest number of *PKS* did not contain syntenic regions in the majority of Asterids species. This suggests that either Asterids never had *PKS* locating to cluster 4, that specific synteny was lost, or the regions/genes were deleted (see Supplemental Table S3).

Syntenic regions exclusively consisting of commelinid species were found in clusters 9, 21, and 27. Furthermore, the clusters 11 and 22 consisted mainly of syntenic regions of commelinids. BLAST analysis of PKS sequences revealed that the annotation of bisdemethoxycurcumin synthases was exclusive for commelinid species. Genes in cluster 9 were mainly annotated as “Other” by pPAP and bisdemethoxycurcumin synthase (BCURS) by BLAST analysis (BCURS are canonically classified as “R-2-X” by pPAP [Shimizu et al., 2017b]). Genes in cluster 22 were mainly annotated as “R-2-X” or “Other” via pPAP and as BCURS by BLAST (type “Other” by pPAP and acridone synthase by BLAST in the *Citrus* genus). These two clusters indicate a commelinid-specific invention of BCURS and PKSs of the “Other” type. Cluster 21 contains genes of unknown function annotated as “Other” by pPAP and CHS-like by BLAST (gray color in Figure 2).

PKSs in the clusters 3 and 11 were almost exclusively classified as “Other” by pPAP and annotated as type III PKS A or CHS-like based on BLAST analysis. PKSs in the clusters 1 and 27 were almost exclusively annotated as “Other” by pPAP and type III PKS B or CHS-like by BLAST (Figure 2). The syntenic cluster 3 showed depletion of syntenic regions of members of the commelinids with the exception of oil palm (*Elaeis guineensis*) and date (*Phoenix dactylera*). Cluster 11 was specific for members of the commelinids. By contrast, syntenic cluster 1 showed depletion of syntenic regions of members of the commelinids with the exception of banana (*Musa acuminata*), great millet (*Sorghum bicolor*), *Oropetium thomaeum*, barley (*Hordeum vulgare*), and *Leersia perrieri*, while the syntenic cluster 27 exclusively contained commelinid *PKS*. Clusters 1 and 27 contained a characterized *LAP5*, while 3 and 11 contain characterized *LAP6* genes (Dobritsa et al., 2010; Kim et al., 2010; e.g. *AT4G34850/LAP5* in cluster 1 and *AT1G02050/LAP6* in cluster 3) and homologs (Supplemental Table S4).

### Synteny network analysis detects four enriched clusters of “R-4-C”-type PKSs corresponding to CHS

pPAP classification revealed further that specific kinds of *PKS* genes showed certain distributions among different clusters. In particular, we found that “R-4-C”-type *PKS* corresponding to the CHS function are overrepresented in the syntenic clusters 2 (and tightly associated 14), 4, and 5 (Fisher’s Exact test, odds ratio: 13.33, *P*-value < 2.2e−16), and the “R-4-C”-classified *PKS* were only found to a minor extent in some other syntenic clusters (Supplemental Figures S3, S4). This observation led to the hypotheses that the major *CHS*-containing clusters 2/14, 4, and 5 either evolved independently three times, *or*, a scenario that is more likely, evolved by duplication of the gene regions following the loss of the synteny between these three *CHS*-enriched clusters.

### Phylogenetic analysis indicates timing of the appearance of “R-4-C”-enriched clusters

**Figure 3:**
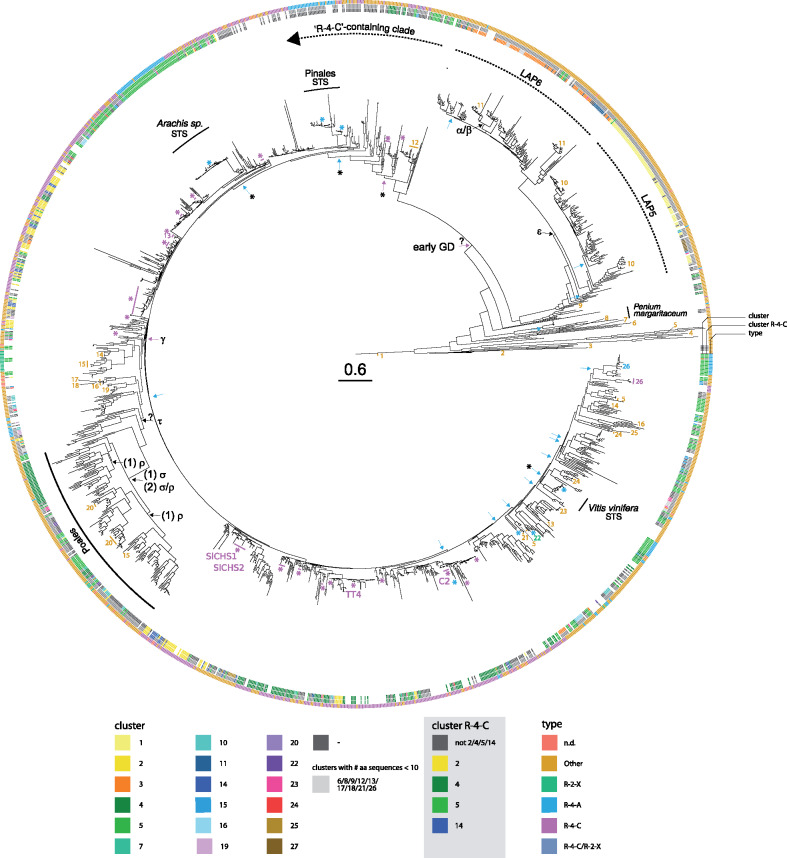
Phylogenetic tree of type III PKSs with information on syntenic cluster membership, and type of the sequence according to pPAP-classification. The phylogenetic tree, based on amino acid sequences, indicates that the LAP ortholog containing clade and the “R-4-C”-containing clade evolved by an early duplication event (early GD). The “R-4-C”-containing syntenic clusters 2/14, 4, and 5 containing clades form highly related but mostly distinct clades in the phylogenetic tree indicating that cluster 2/14, 4, and 5 evolved by duplication events. The LAP5/6 clade contains orthologs of LAP5 and 6 from *A. thaliana* (Supplemental Table S4). Blue arrows indicate “R-4-A” sequences that evolved independently several times. Blue arrows with star (*) indicate STS sequences of *V. vinifera*, *A. duranensis*, *A. ipaensis*, and from gymnosperms. Magenta arrows indicate duplication events involving (proto) “R-4-C”-type PKS sequences. Magenta arrows with a star indicate the origin of “R-4-C” sequences from these events. The gene tree containing 1,607 unique sequences was build using RAxML using 1,000 bootstrap replications. Supplemental Figures S5, S6 show information on the taxonomy. The tree shows high transfer bootstrap expectation values (Lemoine et al., 2018) for all major clades (cf. Supplemental Figure S7). For the pPAP classification of PKS, refer to the legend and Figure 1. Experimentally validated sequences are indicated by numbers. 1: triketide and tetraketide pyrone synthase, PKS18; 2: phloroglucinol synthase; 3: RppA; 4: quinolone synthase; 5: β-ketoacyl carrier protein synthase III; 6: 2'-oxoalkylresorcylic acid synthase, ORAS; 7: CsyB; 8: 2ʹ-oxoalkylresorcinol synthase, ORS; 9: hydroxyalkyl α-pyrone synthase, LAP; 10: hydroxyalkyl α-pyrone synthase, LAP5; 11: hydroxyalkyl α-pyrone synthase, LAP6; 12: stilbenecarboxylate synthase, SCS; 13: valerophenone synthase, VPS, VPS annotated with “R-4-C” also show prenylflavonoid synthase function; 14: diketide-CoA synthase, DCS; 15: curcuminoid synthase, CS/CURS; 16: octaketide synthase, OS; 17: chromone synthase; 18: aleosone synthase; 19: pyrrolidine ketide synthase; 20: alkylresorcylic acid synthase, ARS; 21: acridone synthase, ACS; 22: benzalacetone synthase, BAS; 23: olivetol synthase, OLS: 24: 2-pyrone synthase, 2-PS; 25: orcinol synthase; 26: benzophenone synthase, BPS. A high-resolution version of this figure is available at https://pksevolution.github.io/PKS_visualizations/.

To further evaluate these two hypotheses, we performed a phylogenetic analysis using PKS amino acid sequences to link their syntenic cluster membership with sequence divergence reflected in the phylogenetic tree. The analysis incorporated sequences from over 180 species thereby dramatically extending previous phylogenetic analyses on the type III PKSs (Xie et al., 2016). Here, we included 1,607 different amino acid sequences and obtained detailed phylogenetic relationships using the maximum-likelihood method (Figure 3 and Supplemental Figures S5, S6) having high transfer bootstrap expectation values (Lemoine et al., 2018) for all major clades (Supplemental Figure S7). The phylogenetic tree showed that “R-4-C”/CHSs are mainly present in one very large clade (Figure 3) that is dominated by sequences of the type “R-4-A,” “R-4-C,” and “Other.” This clade also contains the sequences of AT5G13930/TT4 from *A. thaliana*, Solyc09g091510/*Sl*CHS1 and Solyc05g053550/*Sl*CHS2 from *S. lycopersicum* and Zm00001d052673/C2 from *Z. mays*. By contrast, the other clade contains mainly sequences of the type “R-2-X” and “Other.”

Sequences of the type “R-4-C”/CHS corresponding to the syntenic clusters 2, 4, 5, and 14 are located in neighboring, yet mostly distinct subclades. Comparing the “R-4-C”-dominated clade (Figure 3) and the distribution of sequences present in the syntenic clusters (Supplemental Figure S3), it seems likely that the “R-4-C”-type genes in the syntenic clusters originated from the same evolutionary event by duplication/triplication events. From this result, we hypothesize that “R-4-C”-type *PKS* were initially contained in a protocluster that contained primordial “R-4-C”-type *PKS*. This was most likely followed by one or two early duplication events, depending if the LAP or CHS clade evolved first, creating the syntenic protoclusters 2/5/14 and 3, followed by loss of synteny between the protoclusters. Phylogenetic analysis further suggests that type III *PKS* from the type “R-4-C” found in syntenic cluster 5, mainly from the Fabales, originated from type III *PKS* sequences from syntenic cluster 2, since the “R-4-C”-type sequences are located closely in the phylogenetic tree (Figure 3 and Supplemental Figure S5).

Considering the situation that we found for syntenic cluster 5, it seems most probable that the primordial *CHS* genes were initially present in syntenic protocluster 2/14, followed by a loss of synteny between syntenic protocluster 2/14 and 5 (Supplemental Figure S3), leading to the present situation of having four major *CHS*-enriched clusters. Interestingly, “R-4-C”-type *PKS* sequences from *Physcomitrella patens* were detected in the syntenic clusters 5 and 15 while *PKS* from *Azolla filiculoides*, common liverwort (*Marchantia polymorpha*), *Salvinia cucullata*, and *Selaginella moellendorffii* were present in syntenic clusters 4 and/or 5. Within the phylogenetic tree, these sequences located both within the LAP5/6 and the “R-4-C”-containing clade. Furthermore, syntenic cluster 5 contains the duplicated regions of “R-4-A”-type *PKS* sequences of *A. duranensis*, *A. ipaensis*, and *V. vinifera* corresponding to the STS function.

### Independent and multiple evolution of “R-4-A”-type PKS with STS function

The clades containing the “R-4-A”-type STS of *V. vinifera* and of the *Arachis* genus are located within the “R-4-C”-type dominated clade (clusters 2/14, 4 and 5), which suggests that parallel evolution led to the emergence of type “R-4-A”-type PKSs after the emergence of the “R-4-C”-type protocluster. The synteny network and the phylogenetic analysis suggest that syntenic cluster 5 is a relic from syntenic regions that were separated by the emergence of the “R-4-C”-type dominated clade since sequences of syntenic cluster 5 disseminate along the phylogenetic tree (Figure 3). In addition to these early evolutionary events, phylogenetic analysis further highlights several independent evolutionary events underlying the “R-4-A”-type III PKSs. STSs from *V. vinifera* and the *Arachis* genus evolved within the same syntenic cluster 5 independently *or*, although less likely given their evolutionary distances, evolved once and diverged into their sequences while maintaining STS function (Figure 3 and Supplemental Figure S5). Other “R-4-A”-type PKS sequences evolved in several far-related taxa within the monocots, eudicots, and gymnosperms (Figure 3 and Supplemental Figure S5). The gymnosperm-specific “R-4-A”-type PKSs form a monophyletic clade within the phylogenetic tree suggesting that the appearance of “R-4-A” happened before their speciation. These “R-4-A”-type PKSs correspond mostly to pinosylvin-forming STSs and evolved most probably from CHSs or a protoform within the gymnosperms.

As in a previous study (Han et al., 2006), the “R-4-A”-type bibenzyl synthases from the species of the Orchidaceae form a distinct clade from their “R-4-C”-type orthologs, suggesting their divergence into subfamilies before the speciation of this family. Next to the evolution of “R-4-A”-type sequences, we further recapitulated the proposed evolutionary trajectory of VPS from common hop (*Humulus lupulus*) and OLS from *Cannabis sativa* and proposed a possible evolutionary route of aleosone synthase, chromone synthase, and OS in Krantz aloe (*Aloe arborescens*, see Supplemental File S1).

### Combining syntenic network and phylogenetic analysis reveals timing of type III PKS evolution

**Figure 4:**
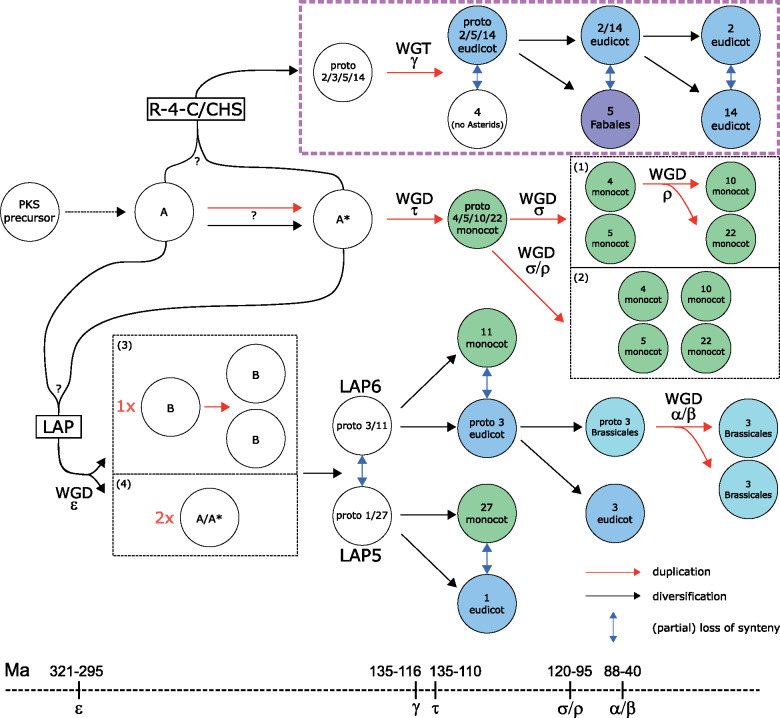
Possible route of evolution of the type III PKS superfamily in the plant kingdom. Given the data, it is unclear if the protocluster evolved into the “R-4-C”-containing clade or the LAP5/6 ortholog clade, first. Possible WGDs and WGTs according to Van de Peer et al. (2017) and Clark and Donoghue (2018) are indicated based on the species distribution, the synteny network, and phylogenetic analysis. Only major syntenic clusters are displayed. The location of clusters does approximately indicate the timing. Hypothesis for evolution of CHSs: from protocluster A or A* diversification into protocluster 2/3/5/14, duplication of protocluster and diversification resulting into cluster 3 and protocluster 2/5/14, diversification of protocluster 2/5/14, and loss of synteny between the three clusters. Hypotheses for evolution of monocot-specific cluster: duplication of A* and diversification into proto 4/5/10/22 cluster, (1) duplication of proto cluster into 4/10/22 and 5, followed by a duplication of 4 resulting in 10 and 22; (2) duplication of monocot-specific proto cluster into 4, 5, 10, and 22. Hypotheses for evolution of LAP5/6 ortholog clade: (3) 1× duplication of A or A* and diversification into protocluster B, subsequent duplication and divergence of this region and loss of synteny. (4) 2× duplication of protoclusters A or A* and loss of synteny between duplicated regions. LAP, LESS ADHESIVE POLLEN; Ma, million years ago; WGT, whole-genome triplication.

Having performed independent phylogenetic and synteny analysis, we next attempted to see what added value could be obtained by combining these analyses. Interestingly, the phylogenetic tree indicates a commelinid-specific clade that mainly contains members of the syntenic clusters 4, 5, 10, and 22. It seems most probable that the *PKS* genes located in syntenic clusters 10 and 22 originate either from a duplication event in clades 5 and 4, resulting in the formation of 5 and proto 4/10/22, followed by a duplication of protocluster 4/10/22 and loss of synteny resulting in 10 and 22 (see (1) in Figure 4) *or* by duplication of the clades 4 and 5 and diversification in the syntenic clusters 4, 5, 10, and 22 and loss of synteny between the syntenic clusters (see (2) in Figure 4). Furthermore, the phylogenetic analysis indicates that the Angiosperm-specific clade containing LAP homologs (Supplemental Table S4) and members of the syntenic clusters 1, 3, 11, and 27 evolved from protoclusters by a segmental duplication event (Figure 3) that we named A and A* in our model of PKS evolution (Figure 4). This clade contains *At*LAP5 or *At*LAP6 orthologs within the Bryophyta, Marchantiophyta, Lycopodiophyta, Pinophyta, Ginkgophyta, and Gnetophyta, instead of *At*CHS orthologs (Supplemental Figure S5). This finding suggests that the LAP5/6 orthologs in this clade originated before the divergence of Angiosperms from these aforementioned tax. The clade containing the LAP5/6 orthologs in angiosperms either originated from one duplication event of the protocluster A leading to the formation of a syntenic cluster B, followed by a second duplication event leading to two regions of B that correspond to the proto 3/11 and proto 1/27 cluster (see (3) in Figure 4) *or* by two duplication events of the regions A or A* leading to the proto 3/11 and proto 1/27 syntenic clusters followed by a loss of synteny between the two protoclusters (see (4) in Figure 4). This clade in the phylogenetic tree indicates that one subclade, either 3/11 or 1/27, originated from the other by a duplication event before the divergence of the monocots and eudicots, followed by loss of synteny between the monocot-specific and eudicot-specific clusters. Within the subclade corresponding to the syntenic clusters 3 and 11, cluster 3 indicates a duplication event within the Brassicales, leading to the emergence of two copies of LAP6 orthologs within the Brassicales, which still share synteny with other members of the syntenic cluster 3 (Figure 2), indicating a recent duplication event. However, a caveat regarding the quality of the gymnosperm genomes should be noted. Currently, no syntenic information is available on these species due to their large genome sized and technical difficulties in their assembly (for details, see Supplemental File S1).

### Drivers of genomic maintenance in syntenic regions

**Figure 5:**
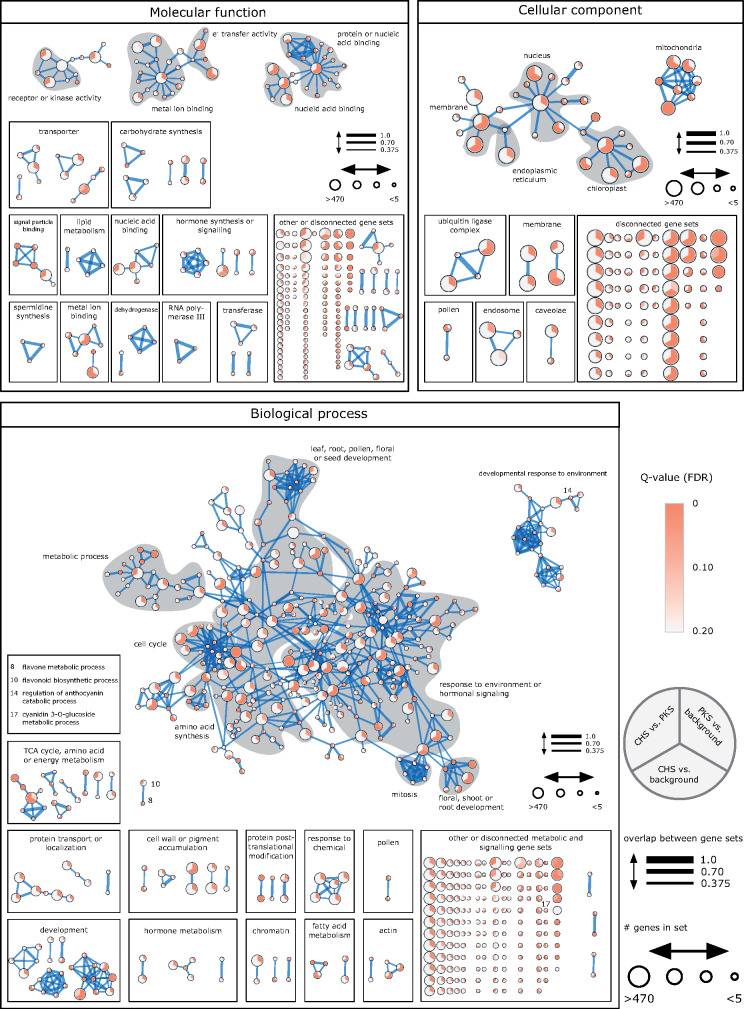
Gene ontology enrichment of syntenic regions (type III *PKS* excluded). *PKS* genes were removed prior to conducting enrichment analysis. Three enrichment sets were compared: A, Genes of *PKS*-containing syntenic regions were checked for enrichment against background (all genes in syntenic regions); B, Genes of syntenic regions in *CHS*-enriched clusters 2, 3, 5, and 14 against background; C, Genes of syntenic regions in *CHS*-enriched clusters 2, 3, 5, and 14 against syntenic genes of all species. The syntenic regions were enriched to a total of 509 (biological process), 230 (molecular function), and 108 (cellular compartment) significant terms (FDR-corrected *q*-value < 0.05). Enriched terms were categorized into higher categories. Many enriched terms in the category “Biological process” can be linked to flavonoid-related processes (“leaf, root, pollen, floral or seed development,” and “response to environment or hormonal signaling”). The size of the vertex corresponds to the number of genes with the same GO term. Terms with a FDR-corrected *q*-value of < 0.2 are displayed. Edges correspond to the similarity between terms based on their gene set overlap (50% Jaccard similarity and 50% overlap between terms with a cutoff of 0.375). FDR, false discovery rate.

The results above show that the genomic regions containing type III *PKS* remain syntenic over a long evolutionary timeframe. This raises the questions why the synteny between *PKS*-containing genomic regions was maintained, and if genes corresponding to specific biological processes contribute to their maintenance. We conducted a GO enrichment analysis on the genes that were reported as being syntenic and checked for enrichment against the background (all genes showing synteny for each species or all genes present in the data sets). We performed this analysis by using syntenic regions containing *PKS* genes (Supplemental Figures S8, S9) and regions where we excluded the *PKS* genes prior to analysis (Figure 5 and Supplemental Figure S10) to remove bias that originates from the presence of *PKS* genes. All comparisons showed similar results in their overrepresentations, with the exception of the comparisons where *PKS* genes were included, which showed specific enrichment of PKS-related terms. We also checked the overrepresentation for GO terms of the *CHS*-enriched syntenic clusters 2, 4, 5, and 14 by performing enrichment tests against the genes of syntenic regions containing *PKS* to test if the *CHS*-enriched syntenic clusters differ from the *PKS*-containing syntenic regions. The analysis did not show fundamentally different results from the other comparisons, rather the terms were specialized terms of those found in the other set comparisons (Supplemental Figure S11). This indicates that the *CHS*-enriched syntenic clusters share an evolutionary past with the *PKS*-containing background.

When excluding *PKS* genes, for the category “Biological process,” the syntenic regions did show few enriched terms that are involved in flavonoid biosynthesis, e.g. “cyanidin 3-*O*-glycoside metabolic process,” “flavonone metabolic process,” or “isoflavonoid metabolic process” (Supplemental Table S5, S6). These terms were found partly in syntenic regions of Brassicales showing large syntenic regions or in a smaller subset of species. The GO enrichment analysis revealed four (flavonoid) regulation-related processes and seven upstream biological processes (Supplemental Tables S5–S8).

Unexpectedly, statistically significant enrichment was detected for GO terms that are related to direct and indirect effects of flavonoids/polyketides on biological processes (genes in syntenic regions: 139 terms, all genes: 149 terms). These terms included processes that are linked to hormone metabolism, growth, development in response to hormonal signaling, and responses to biotic and abiotic stress (Supplemental Tables S5, S6).

## Discussion

During evolution, the biochemical repertoire of plants was expanded to synthesize a myriad of small molecules (Weng et al., 2012; Moghe and Last, 2015). This chemical diversity was achieved by the recruitment of enzymes involved in primary metabolism and duplication of biosynthetic genes (D'Auria, 2006; Falara et al., 2011; Weng et al., 2012; Chae et al., 2014). Here, we presented a large-scale synteny analysis for the type III PKS superfamily in the green lineage and outlined the most likely evolutionary route for biochemical diversification of this important entry point of specialized metabolism. The products of the enzymatic activity of type III PKSs, flavonoids, and building blocks for the pollen exine layer, are ubiquitously distributed in the plant kingdom and have been suggested to be pivotal prerequisites for colonizing the land. Next to the evolution of the lignin pathway, the metabolic innovation of CHS rendered the shikimate pathway, a pathway only found in microorganisms, fungi, and plants, considerably more important (Kubitzki, 1987), such that nowadays it can carry 25% of the total C assimilatory flux (Huang et al., 2010). These innovations render it a high-flux bearing pathway, which is capable of producing a wide spectrum of polyphenolic compounds that play vital roles in development and biotic and abiotic interactions (Fernie, 2019).

### Type III PKSs evolved when colonizing terrestrial ecosystems

For non-land plants (non-embryophytes), that were included in the study here, we found PKS signatures only for some species of the Chlorophyta and *Ectocarpus siliculosus* (Ochrophyta). Chlorophyta are located at the base of green algae, while *E. siliculosus* is from the Chromista kingdom (Cock et al., 2010; Jiao et al., 2020). The PKS signatures from the Chlorophyta do not fall into the generic length range of PKS protein sequences suggesting that these PKS sequences are unlikely complete and functional proteins. However, experimental evidence in support of this supposition is currently lacking. The PKSs from *E. siliculosus* fall within the range of generic PKS sequences found in vascular plants; however, all three sequences are located close to the outgroup of the phylogenetic tree. Previously, the *E. siliculosus* genome was predicted to encode three type III *PKS* that may be involved in phloroglucinol biosynthesis (Cock et al., 2010; Meslet-Cladiere et al., 2013) that correspond to the sequences we found here. The shikimate pathway is also fully encoded in the genome of *E. siliculosus*; however, some downstream pathways, including the pathways leading to phenylpropanoids and salicylic acid, are lacking (Cock et al., 2010; Meslet-Cladiere et al., 2013). Genome mining of other brown algae species detected homologs of type III PKSs (Wong et al., 2007; Pearson et al., 2010; Baharum et al., 2011). However, in the diatoms *Phaeodactylum tricornutum* and *Thalassiosira pseudonana* or in non-photosynthetic stramenopile oomycetes no type III PKSs were found (Meslet-Cladiere et al., 2013). Three putative type III PKSs have been detected in *Pseudochattonella farcimen*, a unicellular alga belonging to the Dictyochophyceae within the Ochrophyta (Dittami et al., 2012). These findings suggest that a lateral gene transfer of type III *PKS*, possibly by an ancestral actinobacterium, occurred following the separation of the diatoms from other members of the Ochrophyta, but before the brown algae diverged from the Pelagophytes and Dictyochophytes (Meslet-Cladiere et al., 2013). Similarly, genes of other pathways, including the genes for mannitol, alginate, and hemicellulose biosynthesis, are present in brown algae but absent in diatoms, suggesting a lateral gene transfer from an ancestral actinobacterium to *E. siliculosus* (Michel et al., 2010a, 2010b; Meslet-Cladiere et al., 2013).

It was previously stated that land plants originated from the Streptophytes of the Charophycean line (de Vries and Archibald, 2018), and that land plants and Zygnematophyceae form a sister relationship (Wodniok et al., 2011; Timme et al., 2012; Ruhfel et al., 2014; Wickett et al., 2014), rather than to the aquatic Chlorophycean group (Kubitzki, 1987). For the two Charophyta species studied where full genomes were available, *Klebsormidium nitens* and *Chara braunii*, no type III PKS sequences were detected. Previously, members of the Charophyceae including the Charales, Coloeochaetales, and Zygnematales, but not basal Charophyceae (Klebsormidiales and Chlorokybales) were shown to have cell walls similar to primary walls of embryophytes. Some of the analyzed Coleochaete have lignin or lignin-like containing cell walls, suggested to be derived from radical coupling of hydroxycinnamyl alcohols (Sorensen et al., 2011). The streptophyte *Klebsormidium flaccidum* harbored an ortholog of the *phenylalanine ammonia-lyase* (*PAL*) gene (Sorensen et al., 2011). de Vries et al. (2017) analyzed the genetic complement in streptophyte and chlorophyte algae with regard to the phenylpropanoid pathway and found cinnamyl/sinapyl alcohol dehydrogenase orthologs, coumarin, lignin, and flavonoid biosynthetic gene orthologs in these taxa, and 4-coumarate:CoA ligase (4CL) among Streptophyta, but not Chlorophyta. These findings indicate that these members of the Streptophyta acquired phenylpropanoid activity, but no activity of type III PKSs *or* that type III PKSs were lost again in the course of evolution, the latter becoming more probable if the hypothesis of Stebbins and Hill that Charophyceae have secondarily returned to an aquatic habitat, after adaptation to terrestrial or amphibian life (Stebbins and Hill, 1980), is correct. Recently, the genomes of *Mesostigma viride*, *Chlorokybus atmophyticus* (Wang et al., 2019), and *P. margaritaceum* (Jiao et al., 2020), members of the Mesostigmatophyceae, Chlorokybophyceae, and Zygnematophyceae, respectively, were published. *Mesostigma viride* and *C. atmophyticus* did not show any type III *PKS* signatures within their genomes. By contrast, *P. margaritaceum* was reported to contain 11 copies of type III *PKS*. This indicates that type III *PKS* appeared earliest with the Zygnematophyceae, but not with the earlier appearing Mesostigmatophyceae, Chlorokybophyceae, Klebsormidiophyceae, and Charophyceae. To our knowledge, no full genome is available yet for a member of the Coleochaetophyceae, the next basal class to the Zygnematophyceae. Such a genome and additional genomes of the Zygnematophyceae represent (an) intriguing model(s) to study further the emergence of type III *PKS* in the green lineage and will yield higher resolution as to which step in evolution type III *PKS* emerged.

### Independent evolution of STS function in different taxa

The evolution of “R-4-A”-type *PKS* was studied previously. Schroder and Schroder (1990) found that STS and CHS sequences differ in charged/uncharged amino acids in homosites across the whole sequences. Tropf et al. (1994) found that STS grouped with CHS sequences in their phylogenetic analysis and subsequently concluded, that STSs evolved from CHSs multiple times independently, while STSs and CHSs have a common evolutionary origin. Their mutagenesis studies showed that three amino acid changes in a CHS hybrid/chimera are sufficient to obtain STS activity. Austin et al. (2004) revealed the structure of functional STS in *Pinus sylvestris* (Scots pine) and identified the structural basis of STS sequences from CHS ancestors corresponding to a cryptic thioesterase activity in the active site, due to an alternative hydrogen bonding network (“aldol switch”). Guided by the STS protein structure, Austin et al. (2004) performed mutagenesis studies to convert CHS into STS. Here, we found similarly that the type “R-4-A” was associated to different clades in the phylogenetic trees supporting previous findings that STSs evolved independently. The clade of gymnosperm STS seemed to appear after the divergence of gymnosperms with angiosperms since the “R-4-A”-type containing clade of gymnosperms locates close to the “R-4-C”- and “Other”-type containing clade of gymnosperms. We found that “R-4-A”-type sequences of *V. vinifera* and *Arachis* sp. are located in the same syntenic cluster. The fact that the STS sequences of *V. vinifera* and *Arachis* sp. do not form a monophyletic clade, however, suggests that independent evolutionary events generated the “R-4-A” function in these species. In the *Arachis* genus, the “R-4-A” function most likely evolved before the speciation of *A. duranensis* and *A. ipaensis* since both “R-4-A”-containing genomic regions locate to the same syntenic cluster and the “R-4-A”-type sequences to the same clade in the phylogenetic tree. Generally, we found that “R-4-A” function is independent of the syntenic regions (Supplemental Figures S3, S4). The phylogenetic analysis suggests that also in gymnosperms the “R-4-A” function evolved before speciation of the gymnosperm species under study.

### Gene ontology indicates no formation of type III PKS-containing gene clusters across the green lineage

The type III *PKS*-containing gene regions maintain their synteny over a long time and across a wide range of taxa (Figure 2 and Supplemental Figures S3, S12, S13). By contrast, some type III *PKS*-containing regions show no synteny to other regions, which could be attributed to low assembly quality (Supplemental Figure S12, C), small scaffold size (Supplemental Figure S12, E, right plot), or to the high taxonomic distance from Angiosperms (Supplemental Figure S12, E).

Plant specialized metabolic pathways may be encoded as regulon-like gene clusters which consist of mostly non-homologous genes that are physically linked and functionally related via biosynthetic pathways and coregulated (Osbourn, 2010). Gene clusters for specialized metabolic pathways were previously shown for alkaloids (Itkin et al., 2013), diterpenoid phytoalexins (Shimura et al., 2007; Miyamoto et al., 2016), triterpenoids (Qi et al., 2004; Field and Osbourn, 2008), hydroxamic acids (Frey et al., 1997), and syringyl lignin (Weng et al., 2011). It was hypothesized that gene clustering provides a selective advantage due to more efficient inheritance since clustered genes are probably retained in the face of recombination. Furthermore, gene clusters allow for coordinate transcription via genomic and epigenetic mechanisms (Weng et al., 2012). In order to test whether this was the case for polyketide/flavonoid biosynthetic gene clusters, we conducted *PKS*-containing syntenic regions a GO enrichment and co-expression analysis for *PKS* genes of the syntenic clusters 2, 4, 5, and 14 using the STRING database (Supplemental File S1). The analyses showed no enriched terms for polyketide-related processes except some upstream-related processes (related to acetate metabolism, shikimate metabolism) and generally no co-expression between *PKS*/*CHS* and other genes.

In barley, a gene cluster consisting of *Cer-c*, a *CHS*-like diketone synthase, *Cer-q*, a lipase/carboxyl transferase, and *Cer-u*, a cytochrome P450 hydroxylase was recently predicted making it, to our knowledge, the only published example to report a gene cluster containing a *PKS* gene (Schneider et al., 2016). *Cer-c* in our study did not show synteny to other *PKS* genes. However, the results presented here render it unlikely that *PKS*s form gene clusters at least in the many species we studied.

Looking at other processes, we found enrichment of processes that showed biological involvement of polyketide products, especially in processes that are linked to hormonal processes and effects. It has been suggested that flavonoid metabolism initially evolved as an internal physiological regulator/chemical messenger (Stafford, 1991), rather than as a UV filter as proposed in Kubitzki (1987), since enzymatic capabilities and enzyme quantities should be low after recruitment from β-ketoacyl ACP synthases (Stafford, 1991). Interestingly, many signaling-related processes were enriched in the genomic context of *PKS* and *CHS* genes. It is possible that *PKS*-related genes governing such processes are an evolutionary remnant *or* provide a direct fitness effect, which maintains type III *PKS* genes in close genomic proximity to these genes. Alternatively or in addition, “hijacking” of genomic regions that contain pivotal genes, such as genes that are linked to transcription and translation, amino acid metabolism, cell development, and responses with the environment, could explain the presence of type III *PKS* in these genomic regions.

### Possible evolutionary route of the type III PKS superfamily, suggested by combined syntenic network and phylogenetic analyses

Type III PKSs catalyze the sequential condensation of acetate units to a starter molecule. The reaction sequence mirrors the biosynthetic pathway of fatty acid synthases in primary metabolism (Austin and Noel, 2003). It has been hypothesized that type III PKSs evolved from β-ketoacyl ACP synthases (Austin and Noel, 2003) due to their similar reaction mechanism and the presence of the αβαβα-fold. Using synteny information and information from a large-scale phylogenetic analyses, we outlined an evolutionary route for the type III PKS superfamily (Figure 4) after the emergence of the first version of a PKS protein. The evolution of type III PKSs is primarily governed by an early gene region or genome duplication that formed the two major “R-4-C”-containing clusters (2, 4, 5, and 14), and an LAP ortholog-containing clade (Figure 3). WGDs have neither been described for the liverwort *M. polymorpha* (Bowman et al., 2017) nor the Lycophyte *S. moellendorffii* (Banks et al., 2011). However, they were described for *P. patens* (Rensing et al., 2008) and the Tracheophyta after the divergence from the Bryophyta (Van de Peer et al., 2017; Clark and Donoghue, 2018), indicating that the “R-4-C”-specific and LAP5/6 clades evolved possibly by a segmental duplication event before the divergence of the Bryophyta from the Tracheophyta.

After the divergence from the Bryophyta and gymnosperms, the LAP5 and LAP6-containing clades (including the Angiosperm-specific clusters 1, 3, 11, and 27) formed by a gene region/genome duplication event, possibly the ε WGD event 321–295 million years ago (Clark and Donoghue, 2017, 2018; Van de Peer et al., 2017), followed by diversification into eudicot- (1 and 3) and monocot-specific (11 and 27) clusters. Within the Brassicales, possibly the α or β WGD event formed the syntenic clusters 14 and 15 88–40 million years ago (Edger et al., 2015; Hohmann et al., 2015; Van de Peer et al., 2017; Clark and Donoghue, 2018).

The members of the LAP5/6-specific clade are anther-specific and involved in sporopollenin biosynthesis (Dobritsa et al., 2010; Kim et al., 2010). Orthologs can also be found in members of the Bryophyta, Marchantiophyta, Lycopodiophyta, Pinophyta, Ginkgophyta, and Gnetophyta. This suggests that LAP5/6 orthologous sequences evolved before the divergence between the former and the Angiosperms by a segmental duplication event. The divergence between gymnosperm- and Angiosperm-forming clades was 365.0–330.9 million years ago in the Carboniferous (Morris et al., 2018; Li et al., 2019b), the divergence between the Bryophyte- and the Tracheophyta-forming clades occurred 506.4–460.3 million years ago (Morris et al., 2018). Since *PKS* can be found in all land plant lines, it can be concluded that the type III *PKS* superfamily is at least 460.3–506.4 million years old.

The two major “R-4-C”-containing regions proto 2/5/14 (containing no “R-4-C” sequences from Monocots) and four evolved possibly by the γ WGT event 135–116 million years ago after the divergence of the Eudicots and the Monocots (Jiao et al., 2012; Van de Peer et al., 2017; Clark and Donoghue, 2018). Intriguingly, we found that the CHSs of *Glycine max* interacting with chalcone reductase (Mameda et al., 2018) locate to syntenic cluster 5 suggesting that a duplication event in the Fabales leading to syntenic cluster 5 facilitated the biosynthesis of isoflavonoids (see Supplemental File S1 for details).

The monocot-specific clades of syntenic clusters 4, 5, 10, and 22 possibly evolved by the τ WGD event 135–110 million years ago (Ming et al., 2015), followed by σ and ρ WGD (see (1) in Figure 4) *or* by the σ or ρ WGD in the Monocot lineage (see (2) in Figure 4) 120–95 million years ago (D’Hont et al., 2012; Ming et al., 2015; Van de Peer et al., 2017; Clark and Donoghue, 2018).

The question remains, which clade evolved directly from the protocluster A, the LAP5/6 clade or the clade containing syntenic clusters 2, 4, 5, 14, and others.

It is interesting to note that sequences of *A. filiculoides* (syntenic cluster 4), *M. polymorpha* (5), and *P. patens* (5, 15), *S. cucullata* (4), and *S. moellendorffii* (4) are located in the “R-4-C”-containing clade *and* the clade containing the *LAP5*/*6* homologs (Figure 3). Most of the sequences in the syntenic cluster 4, 5, and 15 co-locate to the phylogenetic clade containing the syntenic clusters 2, 4, 5, and 14 and show strong synteny to “R-4-C”-containing syntenic clusters, and less to the *LAP*-containing syntenic clusters 1 and 3. This favors the hypothesis that the syntenic clusters 4 and 5 are primordial and existed before the emergence of *LAP5*/*6*-containing syntenic clusters, albeit it has to be kept in mind that not all sequences of the Bryophyta, Lycopodiophyta, Marchantiophyta, and Polypodipsida showed synteny to other sequences, and that the loss of synteny between clusters might differ between different clusters.

Studying the macroevolution of the type III PKS superfamily, the clade containing the syntenic clusters 2, 4, 5, and 14 *or* the clade containing *LAP5/6* evolved from protocluster A *and* the respective other clade evolved by a genome (region) duplication event from protocluster A or A*. Another possibility, although less favored, is that two genome (region) duplication events from A or A* occurred forming the clade containing *LAP5/6* and the syntenic clusters 2, 4, 5, and 14. The “R-4-C”-type enriched syntenic clusters 2, 5, and 14 do not contain monocot “R-4-C” sequences indicating that the diversification into eudicot “R-4-C” happened after the divergence from the monocots *or* that monocot *PKS* genes in the syntenic clusters 2, 5, and 14 lost their “R-4-C” function after diverging from the eudicots. Next to their macroscale evolution, we observed gene expression changes for *CHS* orthologs and conservation of expression pattern for *LAP5*/*6* orthologs (see Supplemental File S1 for details) indicating a diversification of *CHS* orthologs after duplication events.

Sporopollenin is a constituent of the spore and pollen grain outer walls of all known land plants. The average pine sporopollenin structure consists of two fatty acid-derived polyvinyl alcohol-like units, each flanked at one end by a α-pyrone at one end and cross-linked by an ester at the other end. Sporopollenin furthermore possesses supposedly covalently linked *p*-coumaric acid and naringenin as structural units (Li et al., 2019a). Tri- and tetraketide α-pyrones are formed by LAP5 and LAP6 from a broad range of potential acyl-CoA synthetase 5-synthesized fatty acyl-CoA starter substrates (Dobritsa et al., 2010; Kim et al., 2010). Sporopollenin might have equipped algal zygotes with a UV-protecting outer layer that promoted their movement onto the land (Morant et al., 2007; Weng and Chapple, 2010). As some of the most primitive organisms, some members of the freshwater algae Charophytes are believed to host the phenylpropanoid pathway (Kroken et al., 1996; Morant et al., 2007) and it has been suggested that the UV autofluorescent lignin-like material surrounding the zygotes of several charophytic algae species, including *Coleochaete*, is sporopollenin (Delwiche et al., 1989; Kroken et al., 1996; Weng and Chapple, 2010). By contrast, Zygnematophyceae zygospores contain algaenan that differs from pollen grains in its chemical position and the biochemical pathway (acetate–malate pathway) leading to its production (Versteegh and Blokker, 2004). Furthermore, to our knowledge, the presence of type III PKS-like enzymes in the genomes of Charophyta is not reported with the exception of *P. margaritaceum* (Jiao et al., 2020). The adaption of a sporopollenin-containing protecting spore wall is considered a synapomorphy of the embryophytes to colonize the land, but appears to be pre-adaptive given that it is present in the Charophyceae, the proposed sister group to the embryophytes (Delwiche et al., 1989; Kroken et al., 1996; Morant et al., 2007; Weng and Chapple, 2010; Wallace et al., 2011). If *LAP5/6* evolved first, the syntenic clusters 1/27 and/or 3/11 duplicated and formed the primordial cluster of “R-4-C”-type *PKS* genes, followed by a loss of synteny between the clusters 1/27 and 3/11. This sequence of events is consistent with the work of Weng and Chapple (2010) who postulated that the emergence of sporopollenin biosynthesis occurred earlier than that of phenylpropanoid metabolism and flavonoid biosynthesis. To our knowledge, it is not clear if sporopollenin biosynthesis required at this point of emergence the presence of α-pyrone. With the data currently at hand, the exact sequence of events can, however, not be elucidated fully.

In the coming years we expect that more genomes, especially from species of early-diverging lineages will be made available. Such information would allow us to refine the evolutionary sequence we presented here to a higher resolution than is currently possible. This fact notwithstanding, we feel that the study here has allowed us to carry out a comprehensive analysis, and one that is unprecedented in scope of the evolution of the type III PKS family in a manner which we believe is highly applicable to myriad of other specialized pathways of the plant kingdom and beyond.

## Materials and methods

### Retrieval of genomic data and processing of proteome files

Protein FASTA files and .gff/.gff3 files were downloaded for 126 species from the sources indicated in Supplemental Dataset S2. If available, functional annotation files (containing GO annotation and InterPro domains) were downloaded from the same sources. Splice variants, if any were annotated, were removed retaining only the variant with the longest coding sequence for each locus.

Annotated transposable elements in the genomes were removed. Additionally, to further remove TEs and remove TEs in genomes where they were not annotated, a local peptide library was built containing known *A. thaliana*, rice (*Oryza sativa*), tomato (*S. lycopersicum*), and maize (*Z. mays*) transposable elements. All species’ protein FASTA files were queried (using BLAST, default settings) against this database and hits were considered a transposable elements, and removed as well, when the protein identity was >70%, the *E*-value < 0.05 AND the length >50. The proteome files were checked for completeness using BUSCO (Seppey et al., 2019; v4.0.2_cv1, -m proteins, –l chlorophyta_odb10, lineage dataset from 20 November 2019, for the species *C. braunii*, *Chlamydomonas reinhardtii*, *Coccomyxa* sp. *C169*, *Cyanidioschyzon merolae*, *Cyanophora paradoxa*, *Dunaliella salina*, *K. nitens*, *Ostreococcus lucimarinus*, *Volvox carteri, -l stramenopiles_odb10*, lineage dataset from November 21, 2019, for the species *E. siliculosus* and *Aureococcus anophagefferens*, −l cyanobacteria_odb10, lineage dataset from April 24, 2019, *Synechocystis* sp. *PCC 6803*, or -l embryophyta_odb10, lineage dataset from November 20, 2019, for all other species) in the respective docker container.

### Inference of orthogroups, orthologs, and gene families using OrthoFinder and MCL

Orthogroups were inferred from protein FASTA files (proteome files) using OrthoFinder (Emms and Kelly, 2015; v2.2.7), Python (v2.7.10), diamond (v0.9.9), dlcpar (v1.0), fastme (v2.1.5), and mcl (v14.137) using the command orthofinder.py -f ./-S diamond. To obtain MCL groups, pairwise-species BLAST files were used as input for MCL(Enright et al., 2002) clustering using mcxload (–stream-mirror, –stream-neg-log10, -stream-tf “ceil (200),” abc file from BLAST results) and mcl (-I 2; https://micans.org/mcl/).

### Detection of syntenic regions using i-ADHoRe and MCScanX

For each species, information on the gene orientation (+/−) was extracted from the .gff/.gff3 and one file per scaffold/chromosome was created containing the gene (matching the identifier in the protein FASTA file) and its orientation according to the order in the genome. i-ADHoRe (v3.0.01) was used to detect collinear regions between two genomes using the following settings within the .ini file: table_type=family, cluster_type=collinear, alignment_method=gg2, gap_size=15, cluster_gap=20, max_gaps_in_alignment=20, q_value=0.9, prob_cutoff=0.001, anchor_points=5, level_2_only=true, write_stats=true, and number_of_threads=4. For blast_table, the output from OrthoFinder or MCL clustering was used, respectively, where each protein (in the first column) referred to an orthogroup/group (in the second column). MCScanX (mcscanx_h, version 3-28-2013) detected collinear regions between two genomes (using –b 0 option, MATCH_SCORE=50, MATCH_SIZE=5, GAP_PENALTY=−1, OVERLAP_WINDOW=5, E_VALUE=1e−05, MAX GAPS=25) using the homology relations from OrthoFinder or MCL clustering. In the following, all gene pairs were regarded as syntenic that were reported by i-ADHOoRe and MCScanX above the respective thresholds. A selection of sequences of model species was validated for syntenic relationships using the PLAZA database (https://bioinformatics.psb.ugent.be/plaza/).

#### Annotation of type III PKS genes

PKS protein sequences were blasted against the NCBI database and the fit with lowest *E*-value was reported for annotation (expect threshold: 10; word size: 6; matrix: BLOSUM62; Gap costs: existence: 11, Extension: 1; Compositional adjustments: Conditional compositional score matrix adjustment). The same parameters were used when blasting other sequences using blastp against the NCBI database. Classification of the type III PKS reaction type was predicted via pPAP (Shimizu et al., 2017a; v1.1) using the protein sequences as input (ruby v2.5.1p57, BioRuby 1.5.2, MAFFT v7.310, and HMMER v3.2.1). The number of exons was taken from the PLAZA database (Dicots PLAZA 4.0, Monocots PLAZA 4.0, Gymno PLAZA 1.0, pico-PLAZA 2.0).

To retrieve type III PKS sequences in the species *C. atmophyticus* (accession no. RHPI00000000) and *M. viride* (accession no. RHPH00000000; Wang et al., 2019), known CDS sequences of *P. margaritaceum* were blasted against the assemblies of the two species using the following options “Optimize for: Somewhat similar sequences (blastn),” “database: ASM910322v1 GenBank assembly GCA_009103225.1” (*C. atmophyticus*)/“database: ASM974604v1 GenBank assembly GCA_009746045.1” (*M. viride*), “Match score: 2,” “Mismatch score −3,” “Gap costs: Existence: 5, Extension: 2,” “Word size: 7,” “filter low complexity regions,” “mask for lookup table only.” To retrieve type III PKS sequences of members of the Coleochaetaphyceae, we queried the CDS sequences against the nucleotide collection of the Coleochaetaphyceae (taxid: 131209, other algorithm parameters identical as for *C. atmophyticus* and *M. viride*).

### Tandem gene identification and syntenic network construction and clustering

Analysis of synteny was done according to Zhao and Schranz (2017) following a network approach using a custom script. Tandem genes were defined as present when they were detected in one of the four methods. All tandem genes per region (genes that form a component) were treated as a tandem gene region in the following. In a next step and for each method, the value 0.25 was added to *a_i,j_* to adjacency matrix A, if syntenic link between (tandem) gene regions *i* and *j*, containing *PKS* genes, exists. Connections of type “i-ADHoRe+MCL and MCScanX+OrthoFinder” *and* “i-ADHoRe+OrthoFinder and MCScanX+MCL” were removed from the adjacency matrices. Vertices that do not link to others were removed. To determine clusters of the syntenic network, four community structure detection algorithms (all algorithms resulted in ≤20 clusters) were applied separately on the network to retrieve membership: based on greedy optimization of modularity (function fastgreedy.community, modularity=TRUE), via short random walks (function walktrap.community, modularity=TRUE, steps=15), based on the leading eigenvector of the community matrix (function leading.eigenvector.community, steps=15), and based on multi-level optimization of modularity (function multilevel.community, all functions from igraph package v1.2.4.1, R v3.5.0). After this step, distances were calculated for each cluster: if cluster assignment was identical, distance was set to 0, otherwise to 1. The final cluster membership was obtained by affinity propagation clustering for cluster detection using the information from all four cluster detection algorithms (apcluster from the apcluster package, v1.4.6, convits = 1,000, maxits = 10,000, lam = 0.9, nonoise=TRUE, Supplemental Dataset S3). The enrichment test for “R-4-C”-type *PKS* in clusters 2, 4, 5, and 14 (selection criteria: more than 15% of genes are of type “R-4-C” and at least 10 genes in cluster) was performed with the function fisher.test (alternative=“greater”) in the R environment (v3.5.0) after removing the terms for sequences that were not present in the syntenic network. The custom script for network construction can be accessed via www.github.com/tnaake/PKS_synteny.

### Phylogenetic analysis

A multiple sequence alignment was built from characterized PKS protein sequences using MUSCLE (v3.8.31). A HMM protein profile was built using hmmbuild (–fragthresh 0, hmmer v3.2.1). Using hmmsearch, the HMM protein profile was queried against the proteome files. Sequences from species, for which no proteome file is available, were added manually by NCBI database research. Hits were aligned with the protein profile using hmmalign and the alignment was manually checked. Columns with >20% missing values were excluded from further analysis, as well as frayed C- and N-terminal regions of the alignment (Supplemental Dataset S4). Tree building was done by raxmlHPC-AVX (−f a, −m PROTGAMMALGX, −c 25, −p 12345, −x 12345) using 1,000 bootstrap replicates, and the genes AAK45681, BAD97390, and BAA33495 as outgroups. Booster (Lemoine et al., 2018) calculated transfer bootstrap expectation values for branches (−a tbe).

Visualization of the tree was performed within the R environment (v3.5.0) and ggtree (v1.17.1). The phylogenetic species tree was obtained by OrthoFinder via species tree inference from All Genes (STAG; Emms and Kelly, 2019) using the processed proteome files and midpoint rooting in FigTree (v1.4.3).

### Enrichment analysis

Gene ontology terms were obtained for each species separately by using the processed proteome FASTA files and PANNZER2 (Koskinen et al., 2015; Toronen et al., 2018) entering the species name in the field “Scientific name of query species.” Subsequently, three types of enrichment analyses were run: (1) enrichment of genes of *PKS*-containing syntenic regions with no removal of *PKS* genes using the syntenic genes of syntenic regions as background, (2) enrichment of genes of *PKS*-containing syntenic regions with removal of *PKS* genes using the syntenic genes of syntenic regions as background, (3) enrichment of genes of *PKS*-containing syntenic regions with no removal of *PKS* genes using all genes as background (syntenic genes of syntenic regions and other genes), (4) enrichment of genes of *PKS*-containing syntenic regions and all genes as background. GO terms of genes in *PKS*-containing syntenic regions were tested against GO terms of the backgrounds (genes of all syntenic regions or all genes, PKS-BG), genes of “R-4-C”-enriched syntenic regions of clusters 2, 4, 5, and 14 were tested against background (CHS-BG) and genes of “R-4-C”-enriched syntenic regions of clusters 2, 4, 5, and 14 were tested against GO terms of genes within *PKS*-containing syntenic regions (CHS-PKS) using fisher.test (alternative=“greater”) within R (v3.5.0). The enrichment analyses were separately conducted for PKS-BG, CHS-BG, and CHS-PKS and *P*-values were adjusted by Benjamini–Hochberg using p.adjust within the R environment (v3.5.0). Enriched terms were visualized in Cytoscape (Shannon et al., 2003; v3.6.1) using Enrichment Map (Merico et al., 2010; FDR *q*-value cutoff = 0.2, *P*-value cutoff = 0.5, NES [GSEA only]=All, Data Set Edges=Combine edges across data sets [sparser], Cutoff = 0.375, Metric=Jaccard+Overlap combined, Jaccard = 50%, Overlap = 50%). The custom script for enrichment analysis can be accessed via www.github.com/tnaake/PKS_synteny.

### Co-expression analysis using STRING DB

Protein sequence FASTA files were obtained for the genes in *PKS*-containing syntenic regions from the union of all four methods (i-ADHoRe+OrthoFinder, i-ADHoRe+MCL, MCScanX+OrthoFinder, MCScanX+MCL) for all *PKS*-containing syntenic regions from *A. thaliana*, *S. lycopersicum*, *O. sativa*, *Z. mays*, and grape vine (*V. vinifera*). Query sequences with highest identity to STRING proteins were taken as the mapping candidate. Co-expression within syntenic regions were checked by using the STRING DB (Szklarczyk et al., 2015) with the following settings: meaning of network edges=confidence, active interactive sources=Co-expression, minimum required interaction score=medium (0.400), max number of interactors to show: first shell=none, second shell=none).

### Gene expression CoNekT database

Raw expression values were downloaded for *PKS* sequences from the CoNekT database (Proost and Mutwil, 2018; retrieved April 25, 2019) for the species *A. thaliana*, *O. sativa*, *S. moellendorffii*, *S. lycopersicum*, *V. vinifera*, and *Z. mays*. Sampling conditions were categorized into roots/rhizoids, leaves, stem/shoot, fruit/siliques/ear/strobilus/spores, seed, flower, and pollen. Mean values from raw expression values per category were calculated for each gene. Pearson correlation values were calculated between averaged gene expression values using the cor function within R (v3.5.0). Pearson correlation values were clustered by affinity propagation clustering using apcluster (apcluster package, convits = 1,000, maxits = 10,000, nonoise=TRUE seed = 1,000) in R (v3.5.0).

## Accession numbers

The names of all analyzed genes/proteins are mentioned in Supplemental Dataset S1. The names refer either to the identifiers in the NCBI GenBank or to the identifier of the respective genome sequence files (Supplemental Dataset S2).

## Supplemental data

The following materials are available in the online version of this article.

**Supplemental Figure S1.** Phylogenetic tree for 126 analyzed species.

**Supplemental Figure S2.** Number of type III PKS in analyzed species.

**Supplemental Figure S3.** Distribution of type III PKS in syntenic clusters.

**Supplemental Figure S4.** Number of regions and number of type III *PKS* per syntenic cluster.

**Supplemental Figure S5.** Phylogenetic tree of type III PKSs with information on syntenic cluster membership, type of the sequence according to pPAP-classification, and the taxonomic order.

**Supplemental Figure S6.** Phylogenetic tree of type III PKSs with additional information (syntenic cluster membership; type of sequence according to pPAP-classification; number of exons of the gene sequence; and taxonomic information on the family, order, unranked taxonomic information, and class).

**Supplemental Figure S7.** Transfer bootstrap expectation values for phylogenetic gene tree of type III PKSs.

**Supplemental Figure S8.** Gene ontology enrichment of syntenic regions (type III *PKS* included).

**Supplemental Figure S9.** Gene ontology enrichment of syntenic regions (type III PKS included).

**Supplemental Figure S10.** Gene ontology enrichment of syntenic regions (type III PKS excluded).

**Supplemental Figure S11.** Enriched terms for syntenic genes of CHS-enriched clusters versus syntenic genes of type III PKS-containing syntenic regions.

**Supplemental Figure S12.** Quality of synteny network.

**Supplemental Figure S13.** Number of genes in type III *PKS*-containing syntenic regions and size of syntenic regions in kb.

**Supplemental Figure S14.** Co-expression network of syntenic regions in *A. thaliana*, *S. lycopersicum*, *V. vinifera*, and *Z. mays*.

**Supplemental Figure S15.** Gene expression of type III PKS genes of *A. thaliana*, *S. moellendorffii*, *S. lycopersicum*, *V. vinifera*, and *Z. mays*.

**Supplemental Table S1.** Number of type III PKSs for selected species

**Supplemental Table S2.** Species not represented in the synteny network due to missing type III PKS genes, taxonomic distance, and/or low-quality genome assemblies.

**Supplemental Table S3.** Number of type III PKS genes in the network for species belonging to the asterids

**Supplemental Table S4.** Sequences in LAP5/LAP6 ortholog-specific clade

**Supplemental Table S5.** GO enrichment analysis of syntenic regions (genes of syntenic regions)

**Supplemental Table S6.** GO enrichment analysis of syntenic regions (all genes in data set)

**Supplemental Table S7.** Gene information for enriched GO terms of category “Biological process” directly linking to flavonoid metabolism (genes of syntenic regions).

**Supplemental Table S8.** Gene information for enriched GO terms of category “Biological process” directly linking to flavonoid metabolism (all genes in data set).

**Supplemental Dataset S1.** Information on *PKS* genes/amino acids used in phylogenetic and synteny analysis.

**Supplemental Dataset S2.** Data sources of genomes.

**Supplemental Dataset S3.** Information on *PKS* in synteny analysis (tandem and single genes, singletons).

**Supplemental Dataset S4.** Alignment of PKS amino acid sequences.

## Supplementary Material

kiaa086_Supplementary_Data

## Notes

T.N., T.T., H.A.M., and A.R.F. designed the research. T.N. performed and analyzed the transcriptome data and co-expression data. T.N. and A.R.F. performed the phylogenetic analysis. T.N. and S.P. performed the synteny network analysis. T.N. performed the enrichment analysis. T.N. and H.A.M. analyzed the enrichment results. T.N. and A.R.F. wrote the manuscript with input from all authors. A.R.F. agrees to serve as the author responsible for contact and ensures communication.

The author responsible for distribution of materials integral to the findings presented in this article in accordance with the policy described in the Instructions for Authors (https://academic.oup.com/plphys) is: Alisdair R. Fernie (fernie@mpimp-golm.mpg.de).
