# Supplementary material for: Kingdom-wide analysis of the evolution of the plant type III polyketide synthase superfamily
Source: Plant Physiol. 2020 Dec 30;185(3):857–75. doi: 10.1093/plphys/kiaa086 (PMC8133574; doi:10.1093/plphys/kiaa086)
Supplement: kiaa086_Supplementary_Data [file kiaa086_supplementary_data.zip › pp.01200.2020-s04.pdf]

Supplemental Figures

Supplemental Figure S1: Phylogenetic tree for 126 analyzed species. PKS signatures are present in all vascular plants. The species tree was inferred from all genes (STAG) according to Emms and Kelly (2019). Branch length represents the average number of substitution per sites across a gene families. Support value for each bipartition in the consensus STAG tree are the proportion of times that the bipartition is seen in each of the individual species tree estimates. Scale represents substitutions per site. The outgroup was defined by midgroup rooting in FigTree. *: PKS signature present in species as given per OrthoFinder and/or MCL analysis. PKS: polyketide synthase. A high-resolution version of this figure is available at https://pksevolution.github.io/PKS_visualizations/.

Supplemental Figure S2: Number of type III *PKS* in analyzed species. A: All analyzed vascular plants showed at least two copies of *PKS* signatures in their genome. Species with a high number of type III *PKS* belong to the clades Fabales, Poacea, gymnosperms, Marchantiales, Sphagnales, and Funariales. The species are sorted according to their phylogeny. The type of the *PKS* according to the pPAP-classification is displayed in the middle part of the panel on a relative scale (all signatures with length ≤ 200 amino acids removed, refer to Figure 1 for further explanation). The number of *PKS* signatures within the genome is displayed in the right panel for OrthoFinder and MCL separately. B: Number of *PKS* according to OrthoFinder (in total 1,554 signatures) and MCL (1,618 signatures). 1,551 genes are shared between the two detection algorithms. PKS: polyketide synthase.

Supplemental Figure S3: Distribution of type III *PKS* in syntenic clusters. The panel shows a binary matrix that displays if one or more *PKS* or at least one ‘R-4-C’-type *PKS* is present for a certain species given a syntenic cluster. The clusters 2, 4, 5, and 14 are ‘R-4-C’-enriched syntenic clusters. The clusters 1, 3, 11, and 27 contain *LAP5* and *LAP6* homologs. The clusters 11 and 27 show specificity for commelinid species. Species are ordered according to their phylogeny. The number of syntenic genes and the number of syntenic regions per species are shown on the

left. For the pPAP-classification of PKS refer to the legend and Figure 1. LAP: LESS ADHESIVE POLLEN; PKS: polyketide synthase.

Supplemental Figure S4: Number of regions and number of type III *PKS* per syntenic cluster. The clusters 2, 4, 5, and 14 are enriched for the ‘R-4-C’ function referring to CHS. The clusters 1, 3, 11, and 27 contain ‘Other’-type *PKS* genes corresponding to the LAP5 and LAP6 function. For the pPAP-classification of *PKS* refer to the legend and Figure 1.

Supplemental Figure S5: Phylogenetic tree of type III PKSs with information on syntenic cluster membership, type of the sequence according to pPAP-classification and the taxonomic order. The phylogenetic tree, based on amino acid sequences, indicates that the LAP ortholog containing clade and the ‘R-4-C’-containing clade evolved by an early duplication event (early GD). The ‘R-4-C’-containing syntenic clusters 2/14, 4 and 5 containing clades form highly related but mostly distinct clades in the phylogenetic tree indicating that cluster 2/14, 4 and 5 evolved by duplication events. The LAP5/6 clade contains orthologs of LAP5 and 6 from *Arabidopsis thaliana* (Supplemental Table S4). Blue arrows indicate ‘R-4-A’ sequences that evolved independently several times. Blue arrows with star (*) indicate STS sequences of *Vitis vinifera*, *Arachis duranensis*, *Arachis ipaensis* and from gymnosperms. Magenta arrows indicate duplication events involving (proto) ‘R-4-C’-type PKS sequences. Magenta arrows with a star indicate the origin of ‘R-4-C’ sequences from these events. The gene tree containing 1607 unique sequences was build using RAxML using 1000 bootstrap replications. Supplemental Figure S6 shows extended information on the taxonomy. Experimentally validated sequences are font-colored in purple for CHSs (indicated by purple star), blue for STSs (indicated by blue star), green for benzalacetone synthase and orange for other PKS sequences. 1: triketide and tetraketide pyrone synthase, PKS18; 2: phloroglucinol synthase; 3: RppA; 4: quinolone synthase; 5: β-ketoacyl carrier protein synthase III; 6: 2'-oxoalkylresorcylic acid synthase, ORAS; 7: CsyB; 8: 2'-oxoalkylresorcinol

synthase, ORS; 9: hydroxyalkyl α-pyrone synthase, LAP; 10: hydroxyalkyl α-pyrone synthase, LAP5; 11: hydroxyalkyl α-pyrone synthase, LAP6; 12: stilbenecarboxylate synthase, SCS; 13: valerophenone synthase, VPS, VPS annotated with ‘R-4-C’ also show prenylflavonoid synthase function; 14: diketide-CoA synthase, DCS; 15: curcuminoid synthase, CS/CURS; 16: octaketide synthase, OS; 17: chromone synthase; 18: aleosone synthase; 19: pyrrolidine ketide synthase; 20: alkylresorcylic acid synthase, ARS; 21: acridone synthase, ACS; 22: benzalacetone synthase, BAS; 23: olivetol synthase, OLS: 24: 2-pyrone synthase, 2-PS; 25: orcinol synthase; 26: benzophenone synthase, BPS. For the pPAP-classification of PKS refer to the legend and Figure 1. CHS: chalcone synthase; PKS: polyketide synthase; STS: stilbene synthase. A high-resolution version of this figure is available at https://pksevolution.github.io/PKS_visualizations/.

Supplemental Figure S6: Phylogenetic tree of type III PKSs with additional information (syntenic cluster membership, type of sequence according to pPAP-classification, number of exons of the gene sequence, and taxonomic information on the family, order, unranked taxonomic information, and class). The gene tree containing 1607 unique sequences was built using RAxML using 1000 bootstrap replications. The tree shows high transfer bootstrap expectation values (Lemoine et al., 2018) for all major clades. For the pPAP-classification of *PKS* refer to the legend and Figure 1. PKS: polyketide synthase. A high-resolution version of this figure is available at https://pksevolution.github.io/PKS_visualizations/.

Supplemental FigureS7: Transfer bootstrap expectation values for phylogenetic gene tree of type III PKSs. A: Phylogenetic tree with overlaid transfer bootstrap expectation values on nodes. The phylogenetic tree shows high bootstrap values on all major clades. B: Distribution of transfer bootstrap expectation values of the complete tree nodes. Transfer bootstrap expectation values were calculated according to Lemoine et al. (2018). PKS: polyketide synthase. A high-resolution version of this figure is available at https://pksevolution.github.io/PKS_visualizations/.

Supplemental FigureS8: Gene ontology enrichment of syntenic regions (type III *PKS* included). Three enrichment sets were compared: A) Genes of *PKS*-containing syntenic regions were checked for enrichment against background (all genes in syntenic regions), B) Genes of syntenic regions in *CHS*-enriched clusters 2, 4, 5, and 14 against background, C) Genes of syntenic regions in *CHS*-enriched clusters 2, 4, 5 and 14 against syntenic genes of *PKS*-containing syntenic regions of all species. The syntenic regions were enriched to a total of 517 (Biological process), 230 (Molecular function), and 105 (Cellular compartment) significant terms (FDR-corrected q-value < 0.05). Enriched terms were categorized into higher categories. Many enriched terms in the category ‘Biological process’ can be linked to flavonoid-related processes (‘leaf, root, pollen, floral or seed development’, ‘response to environment and hormonal signaling’). The size of the vertex corresponds to the number of genes with the same GO term. Terms with a FDR-corrected q-value of < 0.2 are displayed. Edges correspond to the similarity between terms based on their gene set overlap (50% Jaccard similarity and 50% overlap between terms with a cutoff of 0.375). CHS: chalcone synthase; FDR: false discovery rate; PKS: polyketide synthase.

Supplemental FigureS9: Gene ontology enrichment of syntenic regions (type III *PKS* included). Three enrichment sets were compared: A) Genes of *PKS*-containing syntenic regions were checked for enrichment against background (all genes in data set), B) Genes of syntenic regions in *CHS*-enriched clusters 2, 3, 5, and 14 against background, C) Genes of syntenic regions in *CHS*-enriched clusters 2, 3, 5, and 14 against syntenic genes of *PKS*-containing syntenic regions of all species. The syntenic regions were enriched to a total of 626 (Biological process), 232 (Molecular function), and 109 (Cellular compartment) significant terms (FDR-corrected q-value < 0.05). Enriched terms were categorized into higher categories. Many enriched terms in the category ‘Biological process’ can be linked to flavonoid-related processes (‘leaf, root, pollen, floral or seed development’, ‘response to environment or hormonal signaling’). The size of the vertex corresponds to the number of genes with the same GO term. Terms with a FDR-corrected q-value of < 0.2 are displayed. Edges correspond to the similarity between terms based on their gene set overlap (50% Jaccard similarity and 50% overlap between terms with a

cutoff of 0.375). CHS: chalcone synthase; FDR: false discovery rate; PKS: polyketide synthase.

Supplemental FigureS10: Gene ontology enrichment of syntenic regions (type III *PKS* excluded). *PKS* genes were removed prior to conducting enrichment analysis. Three enrichment sets were compared: A) Genes of *PKS*-containing syntenic regions were checked for enrichment against background (all genes in data set), B) Genes of syntenic regions in *CHS*-enriched clusters 2, 3, 5, and 14 against background, C) Genes of syntenic regions in *CHS*-enriched clusters 2, 3, 5, and 14 against syntenic genes of *PKS*-containing syntenic regions of all species. The syntenic regions were enriched to a total of 553 (Biological process), 224 (Molecular function), and 107 (Cellular compartment) significant terms (FDR-corrected q-value < 0.05). Enriched terms were categorized into higher categories. Many enriched terms in the category ‘Biological process’ can be linked to flavonoid-related processes (‘leaf, root, pollen, floral or seed development’, ‘response to environment or hormonal signaling’). The size of the vertex corresponds to the number of genes with the same GO term. Terms with a FDR-corrected q-value of < 0.2 are displayed. Edges correspond to the similarity between terms based on their gene set overlap (50% Jaccard similarity and 50% overlap between terms with a cutoff of 0.375). CHS: chalcone synthase; FDR: false discovery rate; PKS: polyketide synthase.

Supplemental Figure11: Enriched terms for syntenic genes of *CHS*-enriched clusters vs. syntenic genes of type III *PKS*-containing syntenic regions. Top 20 enriched terms with lowest q-value for ‘Biological process’, ‘Molecular function’, and ‘Cellular compartment’ are shown together with their frequencies in the data sets. A: Data set including *PKS* genes. In total 153 terms for ‘Biological process’, 84 terms for ‘Molecular function’, and 34 terms ‘Cellular compartment’ were found. B: Data set where *PKS* genes were excluded prior to the analysis. In total 144 terms for ‘Biological process’, 83 terms for ‘Molecular function’, and 36 terms for ‘Cellular compartment’ were found. Enrichment based on one-sided Fisher tests with FDR correction. CHS: chalcone synthase; FDR: false discovery rate; PKS: polyketide synthase.

Supplemental Figure S12: Quality of synteny network. A: Synteny network as shown in Figure 2 with information on number of genes of scaffold/chromosome. B: Barplot with information on identity of edges. By highest number, edges are supported by all four methods, followed by 'MCScanX+OrthoFinder' and 'MCScanX+MCL'. C: Barplot showing length distribution of scaffold/chromosome. Left: network, disconnected syntenic regions and disconnected regions with tandem duplications, Right: singletons (vertices that do not connect to other vertices and do not contain tandem duplications). D: Barplot with information on number of PKS types for singletons according to pPAP-classification (refer to Figure 1 for further expalantion). E: Barplot with information on number of singletons per species. OF: OrthoFinder; PKS: polyketide synthase.

Supplemental Figure S13: Number of genes in type III *PKS*-containing syntenic regions and size of syntenic regions in kb. The violin plots comprise the data for all pair-wise comparisons where synteny of *PKS*-containing regions was detected in the two species. For each pair-wise species comparison the sets of minimum and maximum number of genes and size (kb) are displayed separately.

Supplemental Figure14: Co-expression network of syntenic regions in *Arabidopsis thaliana*, *Solanum lycopersicum*, *Vitis vinifera,* and *Zea mays*. Co-expression and associated annotation was inferred from StringDB. Displayed are PKSs of the 'R-4-C' type from the above species in the cluster 2, 4, and 14. Region Os11g32540-Os11g32650 contains the *PKS* genes: LOC_Os11g32540, LOC_Os11g32550, LOC_Os11g32580, LOC_Os11g32610, LOC_Os11g32620, and LOC_Os11g32650. The co-expression of the syntenic region containing the tandem Zm00001d007400 and Zm00001d007403 and the region containing the tandem *PKS* genes Zm00001d052673, Zm00001d052675, and Zm00001d052676 from *Zea mays* is not displayed (no co-expression between *PKS* and other genes in the syntenic region). The analysis was done for all *PKS*-containing syntenic regions from *A. thaliana*, *S. lycopersium*, *O. sativa*, *V. vinifera*, and *Z. mays.* The analysis revealed

that there is no co-expression of *PKS* genes with other genes in *PKS*-containing syntenic regions except for *AT5G13930*. PKS: polyketide synthase.

Supplemental Figure15: Gene expression of type III *PKS* genes of *Arabidopsis thaliana*, *Selaginella moellendorffii*, *Solanum lycopersicum*, *Vitis vinifera,* and *Zea mays*. A: Expression values were taken from the CoNekT database and averaged for the respective tissue. Pearson correlation values were calculated and clusters defined by affinity propagation clustering (clusters I-IV). Heatmap shows Pearson correlation values between the different transcripts. B: Network syntenic cluster membership for expression clusters I-IV as defined in A. The color values correspond to the PKS classification by pPAP (refer to Figure 1 for further explanation). C: Expression profiles of clusters I-IV as defined in A. Displayed are the 25% and 75% quantile of expression values together with the exemplar given by affinity propagation clustering. The analysis showed that the syntenic cluster membership does not dictate the expression pattern of *PKS*, suggesting that the evolution of gene expression is uncoupled from the cluster membership. PKS: polyketide synthase.

Supplemental Tables

Supplemental Table S1: Number of type III PKSs for selected species. Shown are clades with species that have 15 or more copies of type III PKS sequences with length ≥ 200 amino acids together with all species from the same order (shown in brackets):

clade	species	# PKSs (Orthofinder/MCL)	# PKSs ≥ 200 amino acids (Orthofinder/MCL)
Asterales	*Artemisia annua*	16/15	16/15
Ericales	*Camellia sinensis*	18/18	17/17
Fabales	*Arachis duranensis*	33/33	33/33
	Arachis ipaensis	26/25	25/25
	(Cajanus cajan)	15/15	12/12
	Glycine max	24/24	21/21
	Glycyrrhiza uralensis	19/19	17/17
	Lotus japonicus	35/35	16/16
	Medicago truncatula	25/25	25/25
	Phaseolus vulgaris	21/21	13/13
	Trifolium pratense	23/24	16/17
Funariales	*Physcomitrella patens*	30/38	26/28
Ginkgoales	*Ginkgo biloba*	16/16	15/15
Gnetales	(*Gnetum montanum*)	20/22	11/11
Marchantiales	*Marchantia polymorpha*	25/26	24/24
Pinales	(*Picea abies*)	27/27	14/14
	Pinus taeda	42/44	42/44
	Pseudotsuga menziesii	18/18	15/15
Poales, BEP clade of Poaceae	(*Brachypodium distachyon*)	10/11	10/10
	Hordeum vulgare	23/24	20/20
	Leersia perrieri	21/21	20/20
	Oryza rufipogon	27/30	23/23
	Oryza sativa	30/35	28/29
	Triticum aestivum	82/99	50/54
Poales, PACMAD clade of Poaceae	*Setaria italica*	26/27	20/21
	Sorghum bicolor	31/37	30/36
	Zea mays	18/18	16/16
Sapindales	*Citrus clementina*	15/15	15/15
	Citrus sinensis	16/17	14/15

Vitales	*Vitis vinifera*	26/26	17/17
Zingiberales	*Musa acuminata*	21/21	19/19

Supplemental Table S2: Species not represented in the synteny network due to missing type III *PKS* genes, taxonomic distance, and/or low quality genome assemblies. The obtained synteny network (Figure 2) contained syntenic regions of 105 of the initial 126 species; nine species did not contain type III *PKS* sequences, six species did not show synteny/tandem duplications, and six showed only tandem duplications and no synteny due to lower quality genome assemblies.

(possible) cause	species	division	class
no type III *PKS* sequences	*Chara braunii*	Charophyta	Charophyceae
	Chlamydomonas reinhardtii	Chlorophyta	Chlorophyceae
	Cyanidioschyzon merolae	Rhodophyta	Cyanidiophyceae
	Cyanophora paradoxa	Glaucophyta	Glaucophyceae
	Dunaliella salina	Chlorophyta	Chlorophyceae
	Klebsormidium nitens	Charophyta	Klebsormidiophyceae
	Porphyridium purpureum	Rhodophyta	Porphyridiophyceae
	Synechocystis sp. PCC 6803	Cyanophyta	Cyanophyceae
	Volvox carteri	Chlorophyta	Chlorophyceae
no synteny/tandem duplications	*Aureococcus aneophagefferens*	Ochrophyta	Pelagophyceae
	Ectocarpus siliculosus	Ochrophyta	Phaeophyceae
	Coccomyxa sp. C169	Chlorophyta	Trebouxiophyceae
	Genlisea aurea	Magnoliophyta	Magnoliopsida
	Pinus taeda	Pinophyta	Pinopsida
	Ostreococcus lucimarinus	Chlorophyta	Mamiellophyceae
only tandem duplications	*Ginkgo biloba*	Ginkgophyta	Ginkgoopsida
	Gnetum montanum	Gnetophyta	Gnetopsida
	Humulus lupulus	Magnoliophyta	Magnoliopsida
	Picea abies	Pinophyta	Pinopsida
	Pseudotsuga menziessii	Pinophyta	Pinopsida
	Triticum aestivum	Magnoliophyta	Liliopsida

Supplemental Table S3: Number of type III *PKS* genes in the network for species belonging to the Asterids. Syntenic cluster 4 does not contain *PKS*-containing genomic regions of species belonging to Asterids (except *Daucus carota*, carot, and *Vaccinium corymbosum*, northern highbush blueberry). The table shows that for most species of the Asterids, the assembly quality is large (low number of non-connecting genes for most of the species), indicating that the depletion of Asterids in syntenic cluster 4 is most likely not an artefact of low assembly quality:

species	# of PKSs	# of genes in network	# of non-connecting single genes	# of non-connecting tandem-duplicated genes
Actinidia chinensis	10	9	1	0
Artemisia annua	16	1	15	2
Camellia sinensis	18	5	5	8
Capsicum annuum glabriusculum	10	10	-	-
Coffea arabica	7	7	-	-
Daucus carota	14	12	2	-
Erythranthe guttata/ Mimulus guttatus	8	7	1	-
Helianthus annuus	12	12	-	-
Ipomoea nil	7	7	-	-
Petunia axillaris	16	8	3	5
Petunia inflata	9	8	1	-
Nicotiana attenuata	7	3	4	-
Nicotiana benthamiana	14	10	4	-
Nicotiana sylvestris	7	1	6	-
Nicotiana tabacum	11	6	5	-
Olea europaea	7	7	-	-
Salvia miltiorrhiza	5	3	2	
Solanum lycopersicum	7	7	-	-
Solanum pennellii	8	8	-	-
Solanum tuberosum	12	12	-	-
Vaccinium corymbosum	9	8	1	-

Supplemental Table S4: Sequences in LAP5/LAP6 ortholog-specific clade. Given are representatives of Angiosperms sequences in the clade (sequences from *Arabidopsis thaliana*, *Solanum lycopersicum*, *Oryza sativa*, *Zea mays*) and all present sequences of gsperms, Bryophyta, Marchantiophyta, and Pteridophyta. Amino acid sequences from gymnosperms, Bryophyta, Marchantiophyta, and Pteridophyta were blasted against sequences of *A. thaliana* and query cover, identity and E-value was retrieved to the closest orthologue given by blastp:

Gene	cluster	type	ortholog	query cover [%]	identity [%]	E-value
arath_AT1G02050	15	Other	LAP6 (Dobritsa et al., 2010; Kim et al., 2010)			
arath_AT4G00040	14	Other				
solyc_Solyc01g090600.3.1	13		*At*LAP6 (Koenig et al., 2013)			
zeama_Zm00001d032662	18	Other	*At*LAP6 and *Os*LAP6 (Kim et al., 2010)			
zeama_Zm00001d013991	18	Other	*At*LAP6 and *Os*LAP6 (Kim et al., 2010)			
orysa_LOC_Os10g34360	18	Other	CHSL1 (linked to immature panicle) (Kim et al., 2010)			
arath_AT4G34850	9	Other	LAP5 (Dobritsa et al., 2010; Kim et al., 2010)			
solyc_Solyc01g111070.3.1	9	Other	*At*LAP5 (Koenig et al., 2013)			
zeama_Zm00001d019478	19	Other	*At*LAP5 and OsLAP5 (Kim et al., 2010)			
orysa_LOC_Os07g22850	19	Other	CHSL2 (linked to immature panicle) (Besseau et al., 2007)			
picab_MA_619670g0010	-	nd	*At*LAP5	95.0	64.94	5e-67
picab_MA_4931944g0010	-	nd	*At*LAP6	95.0	86.05	8e-45
picab_MA_1748261g0010	-	nd	*At*LAP6	98.0	63.73	9e-38
pseme_PSME_00004131-RA	-	Other	*At*LAP5	96.0	67.72	0.0
pinpi_PPI00030756	not incl.	Other	*At*LAP5	100	67.93	0.0
ginbi_Gb_02579	-	Other	*At*LAP5	96.0	71.84	0.0
gnemo_TnS000356079t05	-	Other	*At*LAP6	81	66.46	5e-152
equgi_cds.Locus_29021_ Transcript_1_1_m.39955	not incl.	Other	*At*LAP5	100	60.33	8e-161
equgi_cds.Locus_25846_Transcript_2_9_m.38839/ equgi_cds.Locus_25846_Transcript_9_9_m.38842	not incl.	nd/nd	*At*LAP5	94.0	60.24	2e-69
sphfa_Sphfalx0085s0032.1	4	Other	*At*LAP5	85.0	56.03	3e-153
sphfa_Sphfalx0160s0011.1	-	Other	*At*LAP5	90.0	58.75	7e-161
marpo_Mapoly0014s0122.1	-	Other	*At*LAP5	94.0	57.32	9e-162
marpo_Mapoly0020s0082.1	-	Other	*At*CHS=AT5G13930	82.0	46.42	3e-117

phypa_Pp3c18_21820V3.1.p	-	nd	-	-	-	-
phypa_Pp3c2_32960V3.1.p	-	Other	*At*LAP5	90.0	58.12	3e-160
selmo_Smo231846|PACid_15419824	4	Other	*At*CHS=AT5G13930	91.0	46.00	1e-65
selmo_Smo122361|PACid_15419808	-	Other	*At*LAP5	95.0	59.06	1e-163
equgi_cds.Locus_10049_Transcript_1_1_m.20667	not incl.	Other	*At*LAP6	94.0	47.18	2e-106
equgi_cds.Locus_462_Transcript_3_4_m.1092	not incl.	Other	*At*LAP5	83.0	44.5	4e-66
equgi_cds.Locus_5106_Transcript_1_2_m.11120/<br>equgi_cds.Locus_5106_Transcript_2_2_m.11121	not incl.	Other	*At*CHS=AT5G13930	73.0	50.00	9e-74
equgi_cds.Locus_1312_Transcript_1_1_m.2882	not incl.	Other	*At*CHS=AT5G13930	88.0	45.99	6e-123

Supplemental Text

Type III *polyketide synthase* copy numbers widely vary among different plant and green algae species

Looking at the species-specific distribution (Supplemental Figure S2), we found differences in the number of type III *PKS* copies among the analyzed species. While we detected PKS protein signatures in all land plant species and various algae [e.g. *Aureococcus aneophagefferens* (Pelagomonodales, Pelagophyta), *Ostreococcus lucimarinus* (Mamiellalles, Chlorophyta), and *Coccomyxa sp. C169* (Chlorococcales, Chlorophyta)], the protein sequences were either shorter (< 300 amino acids) or longer (> 550 amino acids) than the typical length of type III PKS proteins (380-430 amino acids; with the exception of the three *Ectocarpus siliculosus*, Ectocarpales, Phaeophyta, sequences, with 399, 414, and 419 amino acids). Too short sequences will probably result in non-functional proteins due to lacking folds/active sites, though we cannot rule out that a misassembly of the genome may contribute to some of them. Too long sequences are probably β-ketoacyl ACP synthases that show similarity to type III PKSs (the catalytic capability was not tested here). Genomes of species belonging to Chlorophyta, except for the afore-mentioned, and Charophyta, which were available at the time of conducting the analysis, did not have any type III *PKS* (Supplemental Table S2). All analyzed vascular plants showed at least two copies of type III *PKS*. One clade within the Brassicales showed with *Capsella rubella* (pink shepherd's-purse) the lowest number of type III PKSs in its proteome (*n*=2/2, OrthoFinder/MCL, *Arabidopsis thaliana*: *n*=4/4, *AT1G02050*/*LAP6*, *AT4G00040*, *AT4G34850*/*LAP5*, *AT5G13930*/*TT4*). Clades with a high number of type III PKSs (≥ 200 amino acids, Supplemental Table S1) were Fabales (12/12 to 33/33, Supplemental Figure S2), members of the BEP and PACMAD clade of the Poaceae (10/10 *to* 50/54, Supplemental Figure S2*)*, members of gymnosperms (11/11 to 42/44, Supplemental Figure S2), *Marchantia polymorpha* (Marchantiales), and *Physcomitrella patens* (Funariales).

Recently, the full genomes of two members of the Mesostigmaphyceae and Chlorokybophyceae of the Charophyta were released (Wang et al., 2019). To test for the presence of type III *PKS* in these species, we blasted the CDS sequences of the eleven type III *PKS* homologs of *Penium margaritaceum* (Jiao et al., 2020) against

the whole genome shotgun sequences of *Mesostigma viride* and *Chlorokybus atmophyticus*. This analysis resulted in few hits with a maximum query cover of 2% for *Mesostigma viride* and 16% for *Chlorokybus atmophyticus*. We also queried the CDS sequences of *Penium margaritaceum* against all known sequences of the Coleochaetaphyceae yielding hits with ≤ 13% query cover (at the current moment, no full genome of a member belonging to the Coleochaetaphyceae is available). This indicates that there are no type III *PKS* sequences in the reported genomes of the Chlorokybophyceae, Mesostigmaphyceae, or Coleochaetaphyceae. The software tool pPAP (Shimizu et al., 2017) was used to accurately classify unknown type III PKS sequences (for a more detailed description of reaction types refer to Figure 1 in the main text). The pPAP analysis of *Penium* PKS sequences yielded functional diversification (one of 'R-4-A', two of type 'R-4-C', eight of 'Other'); however, some of the *Penium* sequences did not fall within the range of generic type III PKS sequences and could represent non-functional genes or β-ketoacyl ACP synthases. All of the *Penium* sequences were located at the base of the tree before the divergence in the LAP and CHS clade (Figure 3), indicating that only with the conquest of the terrestrial space did type III PKS sequences diversify into the two major clades of the tree.

Other, more basal, algal taxa possess type I PKSs, type II KS proteins, and NRPs: Shelest et al. (2015) studied the distribution of type I PKSs, type II KS proteins, and NRPs in algae for *Chlamydomonas reinhardtii* ($n_{\text{type I PKS}}$=1, $n_{\text{type II KS}}$=4, n_{NRP}=0), *Coccomyxa subellipsoidea* (10, 3, 1), *Ostreococcus lucimarinus* (3, 3, 0), *Volvox carteri* (1, 3, 0; the four former from Chlorophyta), *Cyanophora paradoxa* (0, 0, 1; Glaucophyta), *Aureococcus anophagefferens* (1, 56, 3), *Ectocarpus siliculosus* (1, 5, 0; the two former from Heterokontophyta), *Porphyridium purpureum* (0, 0, 0), *Cyanidioschyzon merolae* (0, 2, 0; the two former from Rhodophyta), and *Klebsormidium flaccidum* (0, 3, 0; Streptophyta) (Shelest et al., 2015), suggesting that these algal taxa possess type I PKSs and type II KSs (with the exception of *Porhyridium purpureum*) instead of type III PKSs, which evolved with or after the conquest of terrestrial habitats.

Typically, the number of 'R-4-C'-type sequences is limited per species to few copies (e.g. one copy in *Arabidopsis thaliana*, two copies in *Solanum lycopersicum*, one

copy in *Oryza sativa*, two copies in *Zea mays*), although their genomes underwent multiple duplication and/or triplication events (Clark and Donoghue, 2018; Van de Peer et al., 2017). This indicates that deletion of duplicated genes or genomic segments containing *CHS* sequences limited the number of *CHS* sequences in a species. Deletion of genes after whole-genome multiplication events was reported previously (Cheng et al., 2012; Moghe et al., 2014; Renny-Byfield et al., 2015; Schnable et al., 2011; Thomas et al., 2006).

It is interesting to note that for 'R-4-A'-type *PKS* referring to the stilbene synthase function we find massive tandem gene duplications in the genome regions (*Vitis vinifera*, *Arachis sp.*, Figure 2), while other 'R-4-A'-type *PKS* sequences do not show gene duplication. Tandem duplication of 'R-4-A'-type *PKS* sequences of the type STS, as the main principle for the generation of *STS* genes, was also found for mulberry (*Morus spp.*) previously (Li et al., 2016).

Evolution of valerophenone synthase in *Humulus lupulus* and olivetol synthase in *Cannabis sativa*

Next to our analysis of the evolution of CHS, LAP, and STS, we analyzed the evolution of valerophenone synthase (VPS) from *Humulus lupulus* and olivetol synthase (OLS) from *Cannabis sativa* (both species from the Cannabaceae family). VPS and OLS are classified according to pPAP as 'Other'. The characterized VPS and OLS sequences (see the labels 13 and 23 in the Figure 3 and Supplemental Figure S5) are located in a separate clade from the 'R-4-C'-type sequences of *Humulus lupulus* and *Cannabis sativa* in the phylogenetic tree.
However, the CHS-type sequences that correspond to CHS_H1 in the study of Novak et al. (2006) (Humlu_CAC19808, humlu_HL.SW.v1.0.G018947.1) also utilize isovaleryl-CoA and isobutyryl-CoA as substrates, although primarily catalyzing the formation of naringenin chalcone. The two corresponding enzymes are annotated as 'R-4-C' by pPAP here. These two sequences are located in the clade containing 'R-4-C' sequences from *Cannabis sativa* and *Humulus lupulus*. The enzyme with the sequence corresponding to VPS (here: Humlu_BAB12012) catalyzes the formation of naringenin chalcone, albeit at lower rates than that encoded by *CHS_H1* indicating, that the sequences in the clade containing Humlu_BAB12012 are VPSs and not CHSs. This suggests that VPS and OLS evolved together with the other

sequences of the type ‘Other’ before the speciation of the two species, forming a monophyletic clade (Figure 3 and Supplemental Figure S5), since this clade is distinct from the clade containing the ‘R-4-C’-type sequences of the two species. Novak et al. (2003) studied the evolution of CHS-like homologues and found that three type III PKS sequences (VPS, CHS3, and CHS4) including VPS are located in the same genomic region. The sequences correspond in this study to humlu_BAB12102, humlu_HL_SW_v1.0_G013555.1, humlu_HL_SW_v1.0_G038363.1 (VPS, type ‘Other’), to humlu_BAB47196, humlu_HL_SW_v1.0_G005266.1 (CHS3, ‘Other’), and to humlu_CAD23044, humlu_ACM17226, humlu_HL_SW_v1.0_G013566.1 (CHS4, ‘Other’). This indicates that the clade containing *Humulus lupulus* and *Cannabis sativa* sequences of the type ‘Other’ originated by gene tandem duplication, and that the genomic region is distinct from the sequences of the ‘R-4-C’ type (CHS_H1 in Novak et al. (2003) corresponding to humlu_CAC19808, humlu_CAK19319, humlu_CAK19318, humlu_ACM17224, humlu_HL_SW_v1.0_G018947.1, see Figure 3 and Supplemental Figure S5).

Evolution of aleosone, chromone, and octaketide synthases in *Aloe arborescens*

Aloe arborescens has the enzymatic capability to synthesize (i) 5,7-dihydroxy-2-methylchromone, from five molecules of malonyl-CoA, by a chromone synthase (Abe et al., 2005b), (ii) octaketides SEK4 and SEK4b, from seven molecules of malonyl-CoA, by an octaketide synthase (Abe et al., 2005a; Mizuuchi et al., 2009), and (iii) aleosone, from seven molecules of malonyl-CoA, by an aleosone synthase (Mizuuchi et al., 2009). The corresponding sequences (Aloar_AAT48709, Aloar_AAX35541, Aloar_ABS72373, Aloar_ACR19997, Aloar_ACR19998, see the labels 16, 17, and 18 in Figure 3 and Supplemental Figure S5) form a monophyletic clade within the phylogenetic tree, indicating that they evolved from the same sequence, possibly by recent tandem duplication or segmental duplication (no full genome is currently available for *Aloe arborescens* to further test these hypotheses). Furthermore, the sequences locate to a clade that contains mainly sequences of the ‘Other’ type from monocots, indicating that these sequences are distinct from the ‘R-4-C’ type, and that they share a similar macroevolutionary trajectory compared to

monocot type III *PKS* sequences belonging to the syntenic clusters 4, 5, 10, and 22 (Figure 3 and Supplemental Figure S5).

Chalcone synthase-containing syntenic regions lack flavonoid biosynthetic gene cluster

To further scrutinize the possibility of flavonoid biosynthetic gene cluster formation within the syntenic regions containing *CHS* genes, we analyzed the co-expression of *CHS* genes with genes in the syntenic regions. To this end, we employed the STRING database to detect functional protein association networks and used the syntenic regions from the model species *Arabidopsis thaliana*, *Oryza sativa*, *Solanum lycopersicum*, and *Vitis vinifera* as input. Specifically, co-expression analysis was conducted using previously characterized or annotated type III *PKS* (*AT5G13930/TT4*, *Os11g32650*, *Solyc05g053550/SlCHS2*, *Solyc09g091510/SlCHS1*, *Solyc12g098100*, *GSVIVT01032968001*; Supplemental Figure S14). Although co-expression networks were detected for all analyzed genes within the *PKS*-containing syntenic regions, they did not show any co-expression with *CHS* except for *AT5G13930/TT4*, which is located in a very large syntenic region on chromosome 5 in *Arabidopsis thaliana* (799 genes were used for STRING database analysis). However, the two co-expressed genes, glutathione S-transferase phi 12 (*AT5G17220*) and UDP-glycosyl transferase 78D2 (*AT5G17050*), were not located in close vicinity to *AT5G13930/TT4*. Generally, we detected functional links using the co-expression that was in agreement with the GO enrichment analysis (e.g. genes that were related to translation and transcription), but the analysis was alleviated by poor annotation of the genes within the syntenic regions for the studied species. Taken together with the GO enrichment analysis, where we found only few terms linked to direct flavonoid biosynthetic process that were enriched for mainly Brassicales species that have long syntenic regions or only present in a subset of species, we conclude that there are no flavonoid-specific biosynthetic gene clusters in the *CHS*-containing regions.

It is important to note that for all gymnosperm species, no information on the syntenic relationship could be obtained due to low scaffold quality of their assembled genomes. From the phylogenetic analysis it seems most probable that gymnosperms

should be located in the syntenic cluster 5 (containing members of Bryophyta, Marchantiophyta, and Polypodiopsida) or syntenic cluster 4 (containing members of Lycopodiophyta and Pteridophyta).

Interaction of chalcone synthase with chalcone reductase might drive macroevolution of chalcone synthases in the Fabales

Intriguingly for the Fabales clade, the concerted biosynthesis of CHS and chalcone reductase (CHR) was reported to be involved in the biosynthesis of isoflavones that are required for the establishment of the symbiosis between root and associated bacteria (Dakora and Phillips, 1996; Graham et al., 2007; Gupta et al., 2018; Mameda et al., 2018; Sepiol et al., 2017; Subramanian et al., 2006; Wasson et al., 2006; Yi et al., 2010). Hereby, CHS interacts with CHR indirectly via 2-hydroxyisoflavonoe synthase isozymes in an isoflavone metabolon (Mameda et al., 2018).

To our knowledge, specific candidate *CHS* genes involved in isoflavone biosynthesis interacting with CHR were proposed only in *Glycine max* (*CHS1* and *CHS7* (Mameda et al., 2018) *and CHS7* and *CHS8* (Sepiol et al., 2017; Yi et al., 2010)). *CHS1*, *CHS7*, and *CHS8* of *Glycine max* located in our study to syntenic cluster 5, the cluster showing ‘R-4-C’-type sequences for all Fabales species (Supplemental Figure S3). It is an interesting hypothesis if other ‘R-4-C’-type sequences in this syntenic cluster are also involved in the biosynthesis of isoflavones, and if syntenic clusters contain specifically those ‘R-4-C’-type sequences required for isoflavone biosynthesis indicating that a duplication event facilitated the biosynthesis of isoflavones in the Fabales.

Multi-tier evolution of type III polyketide synthases: relaxed conservation of gene expression on *chalcone synthase* orthologs and conservation of expression on *LAP5/6* orthologs within syntenic clusters

Besides the evolution changes in a genome-wide context and the divergence of gene sequences, gene expression might also alter, especially after duplication events (Chaudhary et al., 2009; Liu et al., 2011). To test the relationship between gene expression divergence and gene duplication, we evaluated if genes in the

same syntenic network cluster entail similar expression patterns across tissues (Supplemental Figure S15), and if the expression of type III *PKS* genes tend to alter after duplication events. To this end, we obtained expression data of type III *PKS* from the model species *Arabidopsis thaliana*, *Oryza sativa*, *Selaginella moellendorffii*, *Solanum lycopersicum*, *Vitis vinifera,* and *Zea mays* from the CoNekT database (Proost and Mutwil, 2018), and categorized the expression data by tissue across the six species. Based on their Pearson-correlation values (Supplemental Figure S15 A) and affinity propagation clustering, the expression data was partitioned into four expression clusters (I to IV) that are specific to different tissues (Supplemental Figure S15 C). To some extent, gene expression was conserved for the 'R-4-C'-type *PKS* (corresponding to *CHS*) as exemplified by the genes *AT5G13930*/*TT4* from *Arabidopsis thaliana*, *LOC_Os11g32650* from *Oryza sativa*, *Zm00001d007403*/*Whp1,* and *Zm00001d052673*/*C2* from *Zea mays* that were members of expression cluster I showing high expression in flowers and members of the syntenic cluster 4 (Supplemental Figure S15 B). However, other 'R-4-C'-type *PKS* detected in syntenic cluster 2 and 4 (Figure 2) were found in expression cluster III having high expression in reproductive tissues and stem/shoot tissue (*Solyc05g053550.3.1/SlCHS2* and *Solyc09g091510.3.1/SlCHS1* from *Solanum lycopersicum*, *GSVIVT01032968001* from *Vitis vinifera*). Similarly, 'R-4-A'-type *PKS*, assigned to the syntenic cluster 5, were found in both the expression clusters II and III (Supplemental Figure S15 B). These results suggest that even 'R-4-C' and 'R-4-A' type *PKS* from the same syntenic cluster(s) can be expressed differently.

LAP5 and LAP6 homologues (*AT1G02050/LAP6*, *AT4G00040*, *AT4G34850/LAP5* from *Arabidopsis thaliana*, *Smo122361|PACid_15419808,* and *Smo231846|PACid_15419824* from *Selaginella moellendorffii*, *Solyc01g090600.3.1,* and *Solyc01g111070.3.1* from *Solanum lycopersicum*, *GSVIVT01018219001*, *GSVIVT01024107001* from *Vitis vinifera*, and *Zm00001d013991*, *Zm00001d019478*, *Zm00001d032662* from *Zea mays*) showed high expression in flower, fruit/siliques/ear/strobilus/spores, or pollen (Supplemental Figure S15, for *Selaginella moellendorffii* expression information is only available for fruit/siliques/ear/strobilus/spores and leaves; for *Vitis vinifera* no expression information is available for pollen and roots/rhizoids). These transcripts were found in the expression clusters I (*Arabidopsis thaliana*, *Solanum lycopersicum*, and *Zea mays*), III (*Solanum lycopersicum* and *Vitis vinifera*), and IV (*Selaginella*

moellendorffii) (Supplemental Figure S15). Expression values for flower and fruit/siliques/ear/strobilus/spores were not available for *LOC_Os07g22850* and *LOC_Os10g34360*; the two transcripts from *Oryza sativa* showed highest expression in stem/shoot.

The syntenic cluster membership does not dictate the gene expression pattern for the CHS family members analyzed here. Given the occurrence of flavonoids in diverse plant tissues, this observation is likely because the genomic maintenance of syntenic region is overlaid by evolution of transcriptional regulation, and that transcriptional adaptation is happening faster than the loss of synteny of genomic regions. Similarly in *Nicotiana tabacum* (tobacco), in nicotine biosynthesis the non-syntenic *quinolate phosphoribosyltransferase* (*QPT*) *1* and *QPT2*, which evolved by gene duplication in a *Nicotiana* ancestor and have a sequence identity of 94%, exhibit different expression profiles and different response to stress stimuli due to *cis*-regulatory divergence (Moghe and Last, 2015; Shoji and Hashimoto, 2011). The expression of *LAP5* and *LAP6* orthologs coincided with the expected gene expression pattern in all species where expression data was available for all necessary tissues. *LAP5* and *LAP6* orthologs showed expression in flower, fruit/siliques/ear/strobilus/spores, or pollen that overlaps with the localization of sporopollenin biosynthesis. *LAP5* homologs locate to the syntenic clusters 1 and 27, while *LAP6* homologs locate to the syntenic clusters 3 and 11 (cf. Figure 2 and Supplemental Table S3). LAP5 and LAP6 form two distinct clades in the phylogenetic tree (Figure 3); however, the clades do not show inter-cluster synteny. *LAP5*/*6* confer a tissue-specific role in the biosynthesis of sporopollenin, conserving its expression over different syntenic clusters.

References

Abe, I., Oguro, S., Utsumi, Y., Sano, Y., and Noguchi, H. (2005a). Engineered biosynthesis of plant polyketides: chain length control in an octaketide-producing plant type III polyketide synthase. J Am Chem Soc *127*, 12709-12716.

Abe, I., Utsumi, Y., Oguro, S., Morita, H., Sano, Y., and Noguchi, H. (2005b). A plant type III polyketide synthase that produces pentaketide chromone. J Am Chem Soc *127*, 1362-1363.

Adato, A., Mandel, T., Mintz-Oron, S., Venger, I., Levy, D., Yativ, M., Dominguez, E., Wang, Z.H., De Vos, R.C.H., Jetter, R.*, et al.* (2009). Fruit-Surface Flavonoid Accumulation in Tomato Is Controlled by a SlMYB12-Regulated Transcriptional Network. Plos Genetics *5*.

Besseau, S., Hoffmann, L., Geoffroy, P., Lapierre, C., Pollet, B., and Legrand, M. (2007). Flavonoid accumulation in Arabidopsis repressed in lignin synthesis affects auxin transport and plant growth. Plant Cell *19*, 148-162.

Blount, J.W., Dixon, R.A., and Paiva, N.L. (1992). Stress Responses in Alfalfa (Medicago-Sativa L) .16. Antifungal Activity of Medicarpin and Its Biosynthetic Precursors - Implications for the Genetic Manipulation of Stress Metabolites. Physiol Mol Plant P *41*, 333-349.

Bohl, M., Czupalla, C., Tokalov, S.V., Hoflack, B., and Gutzeit, H.O. (2005). Identification of actin as quercetin-binding protein: An approach to identify target molecules for specific ligands. Anal Biochem *346*, 295-299.

Bohl, M., Tietze, S., Sokoll, A., Madathil, S., Pfennig, F., Apostolakis, J., Fahmy, K., and Gutzeit, H.O. (2007). Flavonoids affect actin functions in cytoplasm and nucleus. Biophys J *93*, 2767-2780.

Bottomle.W, Smith, H., and Galston, A.W. (1965). A Phytochrome Mediated Effect of Light on Hydroxylation Pattern of Flavonoids in Pisum Sativum Var Alaska. Nature *207*, 1211-&.

Brown, D.E., Rashotte, A.M., Murphy, A.S., Normanly, J., Tague, B.W., Peer, W.A., Taiz, L., and Muday, G.K. (2001). Flavonoids act as negative regulators of auxin transport in vivo in Arabidopsis. Plant Physiology *126*, 524-535.

Buer, C.S., and Muday, G.K. (2004). The transparent testa4 mutation prevents flavonoid synthesis and alters auxin transport and the response of Arabidopsis roots to gravity and light. Plant Cell *16*, 1191-1205.

Buer, C.S., Sukumar, P., and Muday, G.K. (2006). Ethylene modulates flavonoid accumulation and gravitropic responses in roots of Arabidopsis. Plant Physiology *140*, 1384-1396.

Chaudhary, B., Flagel, L., Stupar, R.M., Udall, J.A., Verma, N., Springer, N.M., and Wendel, J.F. (2009). Reciprocal silencing, transcriptional bias and functional divergence of homeologs in polyploid cotton (gossypium). Genetics *182*, 503-517.

Chen, M.J., Mooney, B.P., Hajduch, M., Joshi, T., Zhou, M.Y., Xu, D., and Thelen, J.J. (2009). System Analysis of an Arabidopsis Mutant Altered in de Novo Fatty Acid Synthesis Reveals Diverse Changes in Seed Composition and Metabolism. Plant Physiology *150*, 27-41.

Chen, M.X., Wang, Z., Zhu, Y.N., Li, Z.L., Hussain, N., Xuan, L.J., Guo, W.L., Zhang, G.P., and Jiang, L.X. (2012). The Effect of TRANSPARENT TESTA2 on Seed Fatty Acid Biosynthesis and Tolerance to Environmental Stresses during Young Seedling Establishment in Arabidopsis. Plant Physiology *160*, 1023-1036.

Cheng, F., Wu, J., Fang, L., Sun, S.L., Liu, B., Lin, K., Bonnema, G., and Wang, X.W. (2012). Biased Gene Fractionation and Dominant Gene Expression among the Subgenomes of Brassica rapa. Plos One *7*.

Clark, J.W., and Donoghue, P.C.J. (2018). Whole-Genome Duplication and Plant Macroevolution. Trends Plant Sci *23*, 933-945.

Coberly, L.C., and Rausher, M.D. (2003). Analysis of a chalcone synthase mutant in Ipomoea purpurea reveals a novel function for flavonoids: amelioration of heat stress. Mol Ecol *12*, 1113-1124.

Coe, E.H., Mccormick, S.M., and Modena, S.A. (1981). White Pollen in Maize. J Hered *72*, 318-320.

Dakora, F.D., and Phillips, D.A. (1996). Diverse functions of isoflavonoids in legumes transcend anti-microbial definitions of phytoalexins. Physiol Mol Plant P *49*, 1-20.

Davies, K.M., Bradley, J.M., Schwinn, K.E., Markham, K.R., and Podivinsky, E. (1993). Flavonoid Biosynthesis in Flower Petals of 5 Lines of Lisianthus (Eustoma-Grandiflorum Grise). Plant Sci *95*, 67-77.

Dehghan, S., Sadeghi, M., Poppel, A., Fischer, R., Lakes-Harlan, R., Kavousi, H.R., Vilcinskas, A., and Rahnamaeian, M. (2014). Differential inductions of phenylalanine ammonia-lyase and chalcone synthase during wounding, salicylic acid treatment, and salinity stress in safflower, Carthamus tinctorius. Bioscience Rep *34*, 273-282.

Dobritsa, A.A., Lei, Z.T., Nishikawa, S., Urbanczyk-Wochniak, E., Huhman, D.V., Preuss, D., and Sumner, L.W. (2010). LAP5 and LAP6 Encode Anther-Specific Proteins with Similarity to Chalcone Synthase Essential for Pollen Exine Development in Arabidopsis. Plant Physiology *153*, 937-955.

Duellpfaff, N., and Wellmann, E. (1982). Involvement of Phytochrome and a Blue-Light Photoreceptor in Uv-B Induced Flavonoid Synthesis in Parsley (Petroselinum-Hortense Hoffm) Cell-Suspension Cultures. Planta *156*, 213-217.

Emms, D.M., and Kelly, S. (2019). OrthoFinder: phylogenetic orthology inference for comparative genomics. Genome Biol *20*, 238.

Espana, L., Heredia-Guerrero, J.A., Reina-Pinto, J.J., Fernandez-Munoz, R., Heredia, A., and Dominguez, E. (2014). Transient Silencing of CHALCONE SYNTHASE during Fruit Ripening Modifies Tomato Epidermal Cells and Cuticle Properties. Plant Physiology *166*, 1371-+.

Fasano, R., Gonzalez, N., Tosco, A., Dal Piaz, F., Docimo, T., Serrano, R., Grillo, S., Leone, A., and Inze, D. (2014). Role of Arabidopsis UV RESISTANCE LOCUS 8 in Plant Growth Reduction under Osmotic Stress and Low Levels of UV-B. Molecular Plant *7*, 773-791.

Fernie, A.R. (2019). Evolution: An Early Role for Flavonoids in Defense against Oomycete Infection. Curr Biol *29*, R688-R690.

French, C.J., and Towers, G.H.N. (1992). Inhibition of Infectivity of Potato-Virus X by Flavonoids. Phytochemistry *31*, 3017-3020.

Gao, Y.F., Liu, J.K., Chen, Y.F., Tang, H., Wang, Y., He, Y.M., Ou, Y.B., Sun, X.C., Wang, S.H., and Yao, Y.A. (2018). Tomato SlAN11 regulates flavonoid biosynthesis and seed dormancy by interaction with bHLH proteins but not with MYB proteins. Hortic Res-England *5*.

Gondor, O.K., Janda, T., Soos, V., Pal, M., Majlath, I., Adak, M.K., Balazs, E., and Szalai, G. (2016). Salicylic Acid Induction of Flavonoid Biosynthesis Pathways in Wheat Varies by Treatment. Frontiers in Plant Science *7*.

Graham, T.L., Graham, M.Y., Subramanian, S., and Yu, O. (2007). RNAi silencing of genes for elicitation or biosynthesis of 5-deoxyisoflavonoids suppresses race-specific resistance and hypersensitive cell death in Phytophthora sojae infected tissues. Plant Physiol *144*, 728-740.

GrantPetersson, J., and Renwick, J.A.A. (1996). Effects of ultraviolet-B exposure of Arabidopsis thaliana on herbivory by two crucifer-feeding insects (Lepidoptera). Environ Entomol *25*, 135-142.

Gu, X.Y., Foley, M.E., Horvath, D.P., Anderson, J.V., Feng, J.H., Zhang, L.H., Mowry, C.R., Ye, H., Suttle, J.C., Kadowaki, K., *et al.* (2011). Association Between Seed Dormancy and Pericarp Color Is Controlled by a Pleiotropic Gene That Regulates Abscisic Acid and Flavonoid Synthesis in Weedy Red Rice. Genetics *189*, 1515-+.

Gupta, R., Min, C.W., Kramer, K., Agrawal, G.K., Rakwal, R., Park, K.H., Wang, Y., Finkemeier, I., and Kim, S.T. (2018). A Multi-Omics Analysis of Glycine max Leaves Reveals Alteration in Flavonoid and Isoflavonoid Metabolism Upon Ethylene and Abscisic Acid Treatment. Proteomics *18*.

Hain, R., Reif, H.J., Krause, E., Langebartels, R., Kindl, H., Vornam, B., Wiese, W., Schmelzer, E., Schreier, P.H., Stocker, R.H., *et al.* (1993). Disease Resistance Results from Foreign Phytoalexin Expression in a Novel Plant. Nature *361*, 153-156.

Hajrah, N.H., Obaid, A.Y., Atef, A., Ramadan, A.M., Arasappan, D., Nelson, C.A., Edris, S., Mutwakil, M.Z., Alhebshi, A., Gadalla, N.O., *et al.* (2017). Transcriptomic analysis of salt stress responsive genes in Rhazya stricta. Plos One *12*.

Heredia, A., Heredia-Guerrero, J.A., and Dominguez, E. (2015). CHS silencing suggests a negative cross-talk between wax and flavonoid pathways in tomato fruit cuticle. Plant Signaling & Behavior *10*.

Hinderer, W., Petersen, M., and Seitz, H.U. (1984). Inhibition of Flavonoid Biosynthesis by Gibberellic-Acid in Cell-Suspension Cultures of Daucus-Carota L. Planta *160*, 544-549.

Hipskind, J.D., and Paiva, N.L. (2000). Constitutive accumulation of a resveratrol-glucoside in transgenic alfalfa increases resistance to Phoma medicaginis. Mol Plant Microbe In *13*, 551-562.

Hsieh, K., and Huang, A.H.C. (2007). Tapetosomes in Brassica tapetum accumulate endoplasmic reticulum-derived flavonoids and alkanes for delivery to the pollen surface. Plant Cell *19*, 582-596.

Jackson, T.L., Baker, G.W., Wilks, F.R., Popov, V.A., Mathur, J., and Benfey, P.N. (2015). Large Cellular Inclusions Accumulate in Arabidopsis Roots Exposed to Low-Sulfur Conditions. Plant Physiology *168*, 1573-U1857.

Jiao, C., Sorensen, I., Sun, X., Sun, H., Behar, H., Alseekh, S., Philippe, G., Palacio Lopez, K., Sun, L., Reed, R., *et al.* (2020). The Penium margaritaceum Genome: Hallmarks of the Origins of Land Plants. Cell *181*, 1097-1111 e1012.

Jiao, Y.T., Xu, W.R., Duan, D., Wang, Y.J., and Nick, P. (2016). A stilbene synthase allele from a Chinese wild grapevine confers resistance to powdery mildew by recruiting salicylic acid signalling for efficient defence. Journal of Experimental Botany *67*, 5841-5856.

Kazuma, K., Noda, N., and Suzuki, M. (2003). Flavonoid composition related to petal color in different lines of Clitoria ternatea. Phytochemistry *64*, 1133-1139.

Kim, S.S., Grienenberger, E., Lallemand, B., Colpitts, C.C., Kim, S.Y., Souza, C.D., Geoffroy, P., Heintz, D., Krahn, D., Kaiser, M., *et al.* (2010). LAP6/POLYKETIDE SYNTHASE A and LAP5/POLYKETIDE SYNTHASE B Encode Hydroxyalkyl alpha-Pyrone Synthases Required for Pollen Development and Sporopollenin Biosynthesis in Arabidopsis thaliana. Plant Cell *22*, 4045-4066.

Koenig, D., Jimenez-Gomez, J.M., Kimura, S., Fulop, D., Chitwood, D.H., Headland, L.R., Kumar, R., Covington, M.F., Devisetty, U.K., Tat, A.V., *et al.* (2013). Comparative transcriptomics reveals patterns of selection in domesticated and wild tomato. P Natl Acad Sci USA *110*, E2655-E2662.

Koyama, R., Roberto, S.R., de Souza, R.T., Borges, W.F.S., Anderson, M., Waterhouse, A.L., Cantu, D., Fidelibus, M.W., and Blanco-Ulate, B. (2018). Exogenous Abscisic Acid Promotes Anthocyanin Biosynthesis and Increased Expression of Flavonoid Synthesis Genes in Vitis vinifera x Vitis labrusca Table Grapes in a Subtropical Region. Frontiers in Plant Science *9*.

Kubasek, W.L., Shirley, B.W., Mckillop, A., Goodman, H.M., Briggs, W., and Ausubel, F.M. (1992). Regulation of Flavonoid Biosynthetic Genes in Germinating Arabidopsis Seedlings. Plant Cell *4*, 1229-1236.

Kuhn, B.M., Errafi, S., Bucher, R., Dobrev, P., Geisler, M., Bigler, L., Zazimalova, E., and Ringli, C. (2016). 7-Rhamnosylated Flavonols Modulate Homeostasis of the Plant Hormone Auxin and Affect Plant Development. Journal of Biological Chemistry *291*, 5385-5395.

Kurepa, J., Shull, T.E., Karunadasa, S.S., and Smalle, J.A. (2018). Modulation of auxin and cytokinin responses by early steps of the phenylpropanoid pathway. Bmc Plant Biol *18*.

Leckband, G., and Lorz, H. (1998). Transformation and expression of a stilbene synthase gene of Vitis vinifera L. in barley and wheat for increased fungal resistance. Theor Appl Genet *96*, 1004-1012.

Lemoine, F., Entfellner, J.B.D., Wilkinson, E., Correia, D., Felipe, M.D., De Oliveira, T., and Gascuel, O. (2018). Renewing Felsenstein's phylogenetic bootstrap in the era of big data. Nature *556*, 452-+.

Lepikson-Neto, J., Nascimento, L.C., Salazar, M.M., Camargo, E.L., Cairo, J.P., Teixeira, P.J., Marques, W.L., Squina, F.M., Mieczkowski, P., Deckmann, A.C., *et al.* (2014).

Flavonoid supplementation affects the expression of genes involved in cell wall formation and lignification metabolism and increases sugar content and saccharification in the fast-growing eucalyptus hybrid E. urophylla x E. grandis. Bmc Plant Biol *14*, 301.

Li, H., Liang, J.B., Chen, H., Ding, G.Y., Ma, B., and He, N.J. (2016). Evolutionary and functional analysis of mulberry type III polyketide synthases. Bmc Genomics *17*.

Li, J.Y., Oulee, T.M., Raba, R., Amundson, R.G., and Last, R.L. (1993). Arabidopsis Flavonoid Mutants Are Hypersensitive to Uv-B Irradiation. Plant Cell *5*, 171-179.

Li, W.Q., Nguyen, K.H., Chu, H.D., Ha, C.V., Watanabe, Y., Osakabe, Y., Leyva-Gonzalez, M.A., Sato, M., Toyooka, K., Voges, L.*, et al.* (2017a). The karrikin receptor KAI2 promotes drought resistance in Arabidopsis thaliana. Plos Genetics *13*.

Li, X., Zhang, L., Ahammed, G.J., Li, Z.X., Wei, J.P., Shen, C., Yan, P., Zhang, L.P., and Han, W.Y. (2017b). Nitric oxide mediates brassinosteroid-induced flavonoid biosynthesis in Camellia sinensis L. J Plant Physiol *214*, 145-151.

Liang, D., Shen, Y.Q., Ni, Z.Y., Wang, Q., Lei, Z., Xu, N.Q., Deng, Q.X., Lin, L.J., Wang, J., Lv, X.L.*, et al.* (2018). Exogenous Melatonin Application Delays Senescence of Kiwifruit Leaves by Regulating the Antioxidant Capacity and Biosynthesis of Flavonoids. Frontiers in Plant Science *9*.

Liu, S.L., Baute, G.J., and Adams, K.L. (2011). Organ and Cell Type-Specific Complementary Expression Patterns and Regulatory Neofunctionalization between Duplicated Genes in Arabidopsis thaliana. Genome Biol Evol *3*, 1419-1436.

Loreti, E., Povero, G., Novi, G., Solfanelli, C., Alpi, A., and Perata, P. (2008). Gibberellins, jasmonate and abscisic acid modulate the sucrose-induced expression of anthocyanin biosynthetic genes in Arabidopsis. New Phytologist *179*, 1004-1016.

Lu, Y.F., Chen, Q., Bu, Y.F., Luo, R., Hao, S.X., Zhang, J., Tian, J., and Yao, Y.C. (2017). Flavonoid Accumulation Plays an Important Role in the Rust Resistance of Malus Plant Leaves. Frontiers in Plant Science *8*.

Lucho-Constantino, G.G., Zaragoza-Martinez, F., Ponce-Noyola, T., Cerda-Garcia-Rojas, C.M., Trejo-Tapia, G., Esparza-Garcia, F., and Ramos-Valdivia, A.C. (2017). Antioxidant responses under jasmonic acid elicitation comprise enhanced production of flavonoids and anthocyanins in Jatropha curcas leaves. Acta Physiol Plant *39*.

Maloney, G.S., DiNapoli, K.T., and Muday, G.K. (2014). The anthocyanin reduced Tomato Mutant Demonstrates the Role of Flavonols in Tomato Lateral Root and Root Hair Development. Plant Physiology *166*, 614-U254.

Mameda, R., Waki, T., Kawai, Y., Takahashi, S., and Nakayama, T. (2018). Involvement of chalcone reductase in the soybean isoflavone metabolon: identification of GmCHR5, which interacts with 2-hydroxyisoflavanone synthase. Plant J *96*, 56-74.

McLusky, S.R., Bennett, M.H., Beale, M.H., Lewis, M.J., Gaskin, P., and Mansfield, J.W. (1999). Cell wall alterations and localized accumulation of feruloyl-3 '-methoxytyramine in onion epidermis at sites of attempted penetration by Botrytis allii are associated with actin polarisation, peroxidase activity and suppression of flavonoid biosynthesis. Plant Journal *17*, 523-534.

Misra, P., Pandey, A., Tiwari, M., Chandrashekar, K., Sidhu, O.P., Asif, M.H., Chakrabarty, D., Singh, P.K., Trivedi, P.K., Nath, P.*, et al.* (2010). Modulation of Transcriptome and Metabolome of Tobacco by Arabidopsis Transcription Factor, AtMYB12, Leads to Insect Resistance. Plant Physiology *152*, 2258-2268.

Mizuuchi, Y., Shi, S.P., Wanibuchi, K., Kojima, A., Morita, H., Noguchi, H., and Abe, I. (2009). Novel type III polyketide synthases from Aloe arborescens. FEBS J *276*, 2391-2401.

Mo, Y.Y., Nagel, C., and Taylor, L.P. (1992). Biochemical Complementation of Chalcone Synthase Mutants Defines a Role for Flavonols in Functional Pollen. P Natl Acad Sci USA *89*, 7213-7217.

Moghe, G.D., Hufnagel, D.E., Tang, H.B., Xiao, Y.L., Dworkin, I., Town, C.D., Conner, J.K., and Shiu, S.H. (2014). Consequences of Whole-Genome Triplication as Revealed by Comparative Genomic Analyses of the Wild Radish Raphanus raphanistrum and Three Other Brassicaceae Species. Plant Cell *26*, 1925-1937.

Moghe, G.D., and Last, R.L. (2015). Something Old, Something New: Conserved Enzymes and the Evolution of Novelty in Plant Specialized Metabolism. Plant Physiol *169*, 1512-1523.

Muhlemann, J.K., Younts, T.L.B., and Muday, G.K. (2018). Flavonols control pollen tube growth and integrity by regulating ROS homeostasis during high-temperature stress. P Natl Acad Sci USA *115*, E11188-E11197.

Nakabayashi, R., Mori, T., and Saito, K. (2014a). Alternation of flavonoid accumulation under drought stress in Arabidopsis thaliana. Plant Signal Behav *9*, e29518.

Nakabayashi, R., Yonekura-Sakakibara, K., Urano, K., Suzuki, M., Yamada, Y., Nishizawa, T., Matsuda, F., Kojima, M., Sakakibara, H., Shinozaki, K.*, et al.* (2014b). Enhancement of oxidative and drought tolerance in Arabidopsis by overaccumulation of antioxidant flavonoids. Plant Journal *77*, 367-379.

Nelson, D.C., Flematti, G.R., Riseborough, J.A., Ghisalberti, E.L., Dixon, K.W., and Smith, S.M. (2010). Karrikins enhance light responses during germination and seedling development in Arabidopsis thaliana. Proc Natl Acad Sci U S A *107*, 7095-7100.

Ng, J.L.P., Hassan, S., Truong, T.T., Hocart, C.H., Laffont, C., Frugier, F., and Mathesius, U. (2015). Flavonoids and Auxin Transport Inhibitors Rescue Symbiotic Nodulation in the Medicago truncatula Cytokinin Perception Mutant cre1. Plant Cell *27*, 2210-2226.

Nikiforova, V., Freitag, J., Kempa, S., Adamik, M., Hesse, H., and Hoefgen, R. (2003). Transcriptome analysis of sulfur depletion in Arabidopsis thaliana: interlacing of biosynthetic pathways provides response specificity. Plant Journal *33*, 633-650.

Novak, P., Krofta, K., and Matousek, J. (2006). Chalcone synthase homologues from Humulus lupulus: some enzymatic properties and expression. Biol Plantarum *50*, 48-54.

Novak, P., Matousek, J., and Briza, J. (2003). Valerophenone synthase-like chalcone synthase homologues in Humulus lupulus. Biol Plantarum *46*, 375-381.

Onkokesung, N., Reichelt, M., van Doorn, A., Schuurink, R.C., van Loon, J.J.A., and Dicke, M. (2014). Modulation of flavonoid metabolites in Arabidopsis thaliana through overexpression of the MYB75 transcription factor: role of kaempferol-3,7-dirhamnoside in resistance to the specialist insect herbivore Pieris brassicae. Journal of Experimental Botany *65*, 2203-2217.

Park, S.S., Bae, I., and Lee, Y.J. (2008). Flavonoids-induced accumulation of hypoxia-inducible factor (HIF)-1 alpha/2 alpha is mediated through chelation of iron. J Cell Biochem *103*, 1989-1998.

Pearce, S., Ferguson, A., King, J., and Wilson, Z.A. (2015). FlowerNet: A Gene Expression Correlation Network for Anther and Pollen Development. Plant Physiology *167*, 1717-U1923.

Peer, W.A., and Murphy, A.S. (2007). Flavonoids and auxin transport: modulators or regulators? Trends Plant Sci *12*, 556-563.

Petridis, A., Doll, S., Nichelmann, L., Bilger, W., and Mock, H.P. (2016). Arabidopsis thaliana G2-LIKE FLAVONOID REGULATOR and BRASSINOSTEROID ENHANCED EXPRESSION1 are low-temperature regulators of flavonoid accumulation. New Phytologist *211*, 912-925.

Pi, E.X., Zhu, C.M., Fan, W., Huang, Y.Y., Qu, L.Q., Li, Y.Y., Zhao, Q.Y., Ding, F., Qiu, L.J., Wang, H.Z.*, et al.* (2018). Quantitative Phosphoproteomic and Metabolomic Analyses Reveal GmMYB173 Optimizes Flavonoid Metabolism in Soybean under Salt Stress. Mol Cell Proteomics *17*, 1209-1224.

Proost, S., and Mutwil, M. (2018). CoNekT: an open-source framework for comparative genomic and transcriptomic network analyses. Nucleic Acids Res *46*, W133-W140.

Quilichini, T.D., Samuels, A.L., and Douglas, C.J. (2014). ABCG26-Mediated Polyketide Trafficking and Hydroxycinnamoyl Spermidines Contribute to Pollen Wall Exine Formation in Arabidopsis. Plant Cell *26*, 4483-4498.

Renny-Byfield, S., Gong, L., Gallagher, J.P., and Wendel, J.F. (2015). Persistence of Subgenomes in Paleopolyploid Cotton after 60 My of Evolution. Mol Biol Evol *32*, 1063-1071.

Ribeiro, D.M., Araujo, W.L., Fernie, A.R., Schippers, J.H.M., and Mueller-Roeber, B. (2012). Translatome and metabolome effects triggered by gibberellins during rosette growth in Arabidopsis. Journal of Experimental Botany *63*, 2769-2786.

Ringli, C., Bigler, L., Kuhn, B.M., Leiber, R.M., Diet, A., Santelia, D., Frey, B., Pollmann, S., and Klein, M. (2008). The modified flavonol glycosylation profile in the Arabidopsis rol1 mutants results in alterations in plant growth and cell shape formation. Plant Cell *20*, 1470-1481.

Santelia, D., Henrichs, S., Vincenzetti, V., Sauer, M., Bigler, L., Klein, M., Bailly, A., Lee, Y., Friml, J., Geisler, M.*, et al.* (2008). Flavonoids redirect PIN-mediated polar auxin fluxes during root gravitropic responses. J Biol Chem *283*, 31218-31226.

Sarker, U., and Oba, S. (2019). Salinity stress enhances color parameters, bioactive leaf pigments, vitamins, polyphenols, flavonoids and antioxidant activity in selected Amaranthus leafy vegetables. J Sci Food Agr *99*, 2275-2284.

Schnable, J.C., Springer, N.M., and Freeling, M. (2011). Differentiation of the maize subgenomes by genome dominance and both ancient and ongoing gene loss. P Natl Acad Sci USA *108*, 4069-4074.

Schulz, E., Tohge, T., Zuther, E., Fernie, A.R., and Hincha, D.K. (2015). Natural variation in flavonol and anthocyanin metabolism during cold acclimation in Arabidopsis thaliana accessions. Plant Cell Environ *38*, 1658-1672.

Schulz, E., Tohge, T., Zuther, E., Fernie, A.R., and Hincha, D.K. (2016). Flavonoids are determinants of freezing tolerance and cold acclimation in Arabidopsis thaliana. Sci Rep *6*, 34027.

Sepiol, C.J., Yu, J.J., and Dhaubhadel, S. (2017). Genome-Wide Identification of Chalcone Reductase Gene Family in Soybean: Insight into Root-Specific GmCHRs and Phytophthora sojae Resistance. Frontiers in Plant Science *8*.

Shelest, E., Heimerl, N., Fichtner, M., and Sasso, S. (2015). Multimodular type I polyketide synthases in algae evolve by module duplications and displacement of AT domains in trans. Bmc Genomics *16*.

Shimizu, Y., Ogata, H., and Goto, S. (2017). Discriminating the reaction types of plant type III polyketide synthases. Bioinformatics *33*, 1937-1943.

Shoji, T., and Hashimoto, T. (2011). Recruitment of a duplicated primary metabolism gene into the nicotine biosynthesis regulon in tobacco. Plant J *67*, 949-959.

Silva-Navas, J., Moreno-Risueno, M.A., Manzano, C., Tellez-Robledo, B., Navarro-Neila, S., Carrasco, V., Pollmann, S., Gallego, F.J., and del Pozo, J.C. (2016). Flavonols Mediate Root Phototropism and Growth through Regulation of Proliferation-to-Differentiation Transition. Plant Cell *28*, 1372-1387.

Sotelo-Silveira, M., Cucinotta, M., Chauvin, A.L., Montes, R.A.C., Colombo, L., Marsch-Martinez, N., and de Folter, S. (2013). Cytochrome P450 CYP78A9 Is Involved in Arabidopsis Reproductive Development. Plant Physiology *162*, 779-799.

Subramanian, S., Stacey, G., and Yu, O. (2006). Endogenous isoflavones are essential for the establishment of symbiosis between soybean and Bradyrhizobium japonicum. Plant Journal *48*, 261-273.

Tan, J.F., Tu, L.L., Deng, F.L., Hu, H.Y., Nie, Y.C., and Zhang, X.L. (2013). A Genetic and Metabolic Analysis Revealed that Cotton Fiber Cell Development Was Retarded by Flavonoid Naringenin. Plant Physiology *162*, 86-95.

Taulavuori, K., Hyoky, V., Oksanen, J., Taulavuori, E., and Julkunen-Tiitto, R. (2016). Species-specific differences in synthesis of flavonoids and phenolic acids under increasing periods of enhanced blue light. Environ Exp Bot *121*, 145-150.

Thomas, B.C., Pedersen, B., and Freeling, M. (2006). Following tetraploidy in an Arabidopsis ancestor, genes were removed preferentially from one homeolog leaving clusters enriched in dose-sensitive genes. Genome Research *16*, 934-946.

Thomas, H., Huang, L., Young, M., and Ougham, H. (2009). Evolution of plant senescence. Bmc Evol Biol *9*.

Thompson, E.P., Wilkins, C., Demidchik, V., Davies, J.M., and Glover, B.J. (2010). An Arabidopsis flavonoid transporter is required for anther dehiscence and pollen development. Journal of Experimental Botany *61*, 439-451.

Toffolatti, S.L., Venturini, G., Maffi, D., and Vercesi, A. (2012). Phenotypic and histochemical traits of the interaction between Plasmopara viticola and resistant or susceptible grapevine varieties. Bmc Plant Biol *12*.

Van de Peer, Y., Mizrachi, E., and Marchal, K. (2017). The evolutionary significance of polyploidy. Nat Rev Genet *18*, 411-424.

Vanhaelewyn, L., Viczian, A., Prinsen, E., Bernula, P., Serrano, A.M., Arana, M.V., Ballare, C.L., Nagy, F., Van Der Straeten, D., and Vandenbussche, F. (2019). Differential UVR8 Signal across the Stem Controls UV-B-Induced Inflorescence Phototropism. Plant Cell *31*, 2070-2088.

Wan, J.P., Zhang, P., Wang, R.L., Sun, L.L., Wang, W.Y., Zhou, H.K., and Xu, J. (2018). UV-B Radiation Induces Root Bending Through the Flavonoid-Mediated Auxin Pathway in Arabidopsis. Frontiers in Plant Science *9*.

Wang, S., Li, L., Li, H., Sahu, S.K., Wang, H., Xu, Y., Xian, W., Song, B., Liang, H., Cheng, S.*, et al.* (2019). Genomes of early-diverging streptophyte algae shed light on plant terrestrialization. Nat Plants.

Wasson, A.P., Pellerone, F.I., and Mathesius, U. (2006). Silencing the flavonoid pathway in Medicago truncatula inhibits root nodule formation and prevents auxin transport regulation by rhizobia. Plant Cell *18*, 1617-1629.

Watkins, J.M., Chapman, J.M., and Muday, G.K. (2017). Abscisic Acid-Induced Reactive Oxygen Species Are Modulated by Flavonols to Control Stomata Aperture. Plant Physiology *175*, 1807-1825.

Yamasaki, H., Sakihama, Y., and Ikehara, N. (1997). Flavonoid-peroxidase reaction as a detoxification mechanism of plant cells against H2O2. Plant Physiology *115*, 1405-1412.

Yang, H.X., Liu, H., Li, G., Feng, J.J., Qin, H.J., Liu, X., Xue, H.W., and Wang, D.W. (2009). Reduction of root flavonoid level and its potential involvement in lateral root emergence in Arabidopsis thaliana grown under low phosphate supply. Funct Plant Biol *36*, 564-573.

Yi, J., Derynck, M.R., Chen, L., and Dhaubhadel, S. (2010). Differential expression of CHS7 and CHS8 genes in soybean. Planta *231*, 741-753.

Ylstra, B., Touraev, A., Moreno, R.M.B., Stoger, E., Vantunen, A.J., Vicente, O., Mol, J.N.M., and Heberlebors, E. (1992). Flavonols Stimulate Development, Germination, and Tube Growth of Tobacco Pollen. Plant Physiology *100*, 902-907.

Zuk, M., Dzialo, M., Richter, D., Dyminska, L., Matula, J., Kotecki, A., Hanuza, J., and Szopa, J. (2016). Chalcone Synthase (CHS) Gene Suppression in Flax Leads to Changes in Wall Synthesis and Sensing Genes, Cell Wall Chemistry and Stem Morphology Parameters. Frontiers in Plant Science *7*.

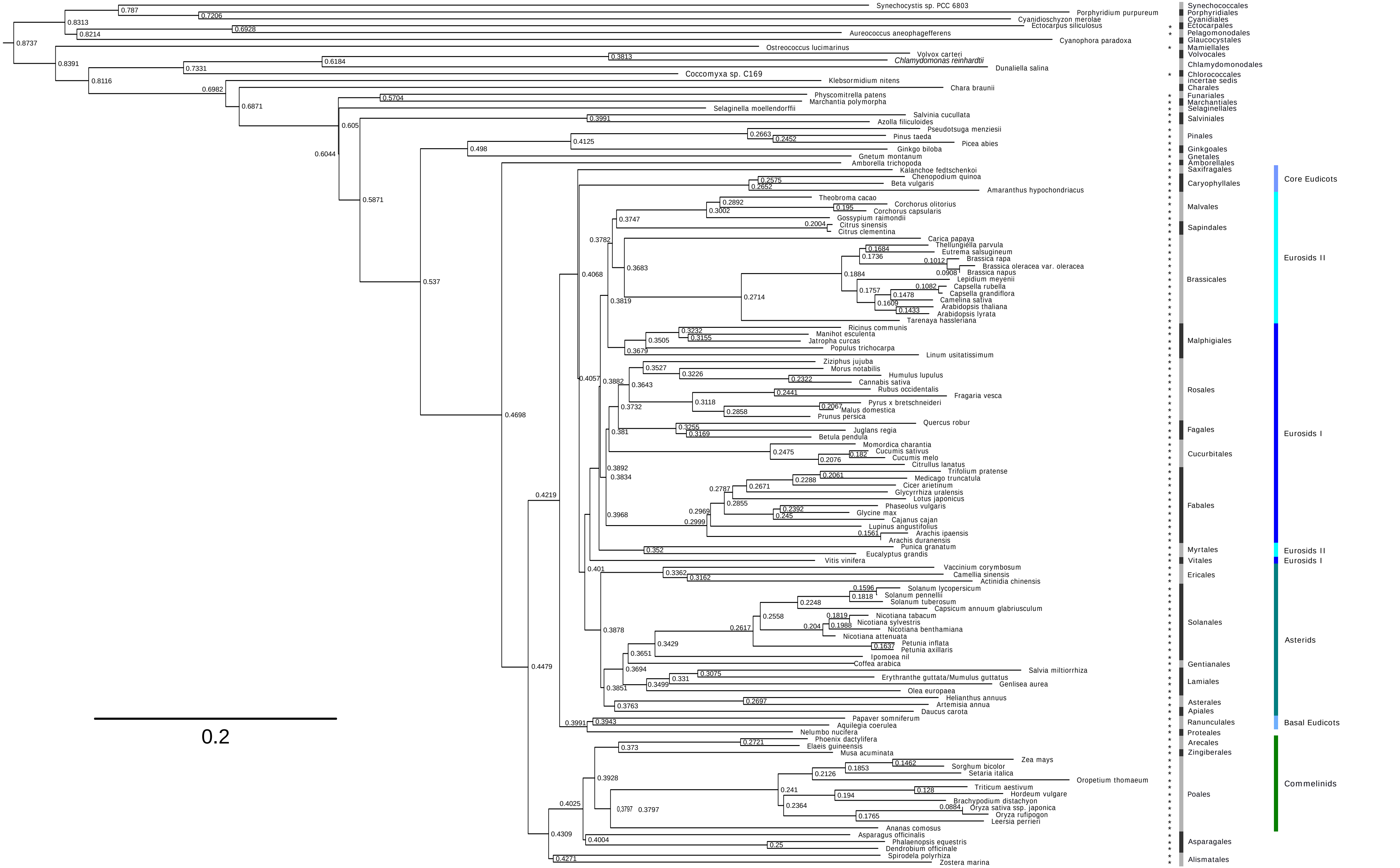
Synechocystis sp. PCC 6803
Porphyridium purpureum
Cyanidioschyzon merolae
Ectocarpus siliculosus
Aureococcus aneophagefferens
Cyanophora paradoxa
Ostreococcus lucimarinus
Volvox carteri
Chlamydomonas reinhardtii
Dunaliella salina
Coccomyxa sp. C169
Klebsormidium nitens
Chara braunii
Physcomitrella patens
Marchantia polymorpha
Selaginella moellendorffii
Salvinia cucullata
Azolla filiculoides
Pseudotsuga menziesii
Pinus taeda
Picea abies
Ginkgo biloba
Gnetum montanum
Amborella trichopoda
Kalanchoe fedtschenkoi
Chenopodium quinoa
Beta vulgaris
Amaranthus hypochondriacus
Theobroma cacao
Corchorus olitorius
Corchorus capsularis
Gossypium raimondii
Citrus sinensis
Citrus clementina
Carica papaya
Thellungiella parvula
Eutrema salsugineum
Brassica rapa
Brassica oleracea var. oleracea
Brassica napus
Lepidium meyenii
Capsella rubella
Capsella grandiflora
Camelina sativa
Arabidopsis thaliana
Arabidopsis lyrata
Tarenaya hassleriana
Ricinus communis
Manihot esculenta
Jatropha curcas
Populus trichocarpa
Linum usitatissimum
Ziziphus jujuba
Morus notabilis
Humulus lupulus
Cannabis sativa
Rubus occidentalis
Fragaria vesca
Pyrus x bretschneideri
Malus domestica
Prunus persica
Quercus robur
Juglans regia
Betula pendula
Momordica charantia
Cucumis sativus
Cucumis melo
Citrullus lanatus
Trifolium pratense
Medicago truncatula
Cicer arietinum
Glycyrrhiza uralensis
Lotus japonicus
Phaseolus vulgaris
Glycine max
Cajanus cajan
Lupinus angustifolius
Arachis ipaensis
Arachis duranensis
Punica granatum
Eucalyptus grandis
Vitis vinifera
Vaccinium corymbosum
Camellia sinensis
Actinidia chinensis
Solanum lycopersicum
Solanum pennellii
Solanum tuberosum
Capsicum annuum glabriusculum
Nicotiana tabacum
Nicotiana sylvestris
Nicotiana benthamiana
Nicotiana attenuata
Petunia inflata
Petunia axillaris
Ipomoea nil
Coffea arabica
Salvia miltiorrhiza
Erythranthe guttata/Mumulus guttatus
Genlisea aurea
Olea europaea
Helianthus annuus
Artemisia annua
Daucus carota
Papaver somniferum
Aquilegia coerulea
Nelumbo nucifera
Phoenix dactylifera
Elaeis guineensis
Musa acuminata
Zea mays
Sorghum bicolor
Setaria italica
Oropetium thomaeum
Triticum aestivum
Hordeum vulgare
Brachypodium distachyon
Oryza sativa ssp. japonica
Oryza rufipogon
Leersia perrieri
Ananas comosus
Asparagus officinalis
Phalaenopsis equestris
Dendrobium officinale
Spirodela polyrhiza
Zostera marina
Synechococcales
Porphyridiales
Cyanidiales
Ectocarpales
Pelagomonodales
Glaucocystales
Mamiellales
Volvocales
Chlamydomonodales
Chlorococcales
incertae sedis
Charales
Funariales
Marchantiales
Selaginellales
Salviniales
Pinales
Ginkgoales
Gnetales
Amborellales
Saxifragales
Caryophyllales
Malvales
Sapindales
Brassicales
Malphigiales
Rosales
Fagales
Cucurbitales
Fabales
Myrtales
Vitales
Ericales
Solanales
Gentianales
Lamiales
Asterales
Apiales
Ranunculales
Proteales
Arecales
Zingiberales
Poales
Asparagales
Alismatales
Core Eudicots
Eurosids II
Eurosids I
Eurosids II
Eurosids I
Asterids
Basal Eudicots
Commelinids
0.2

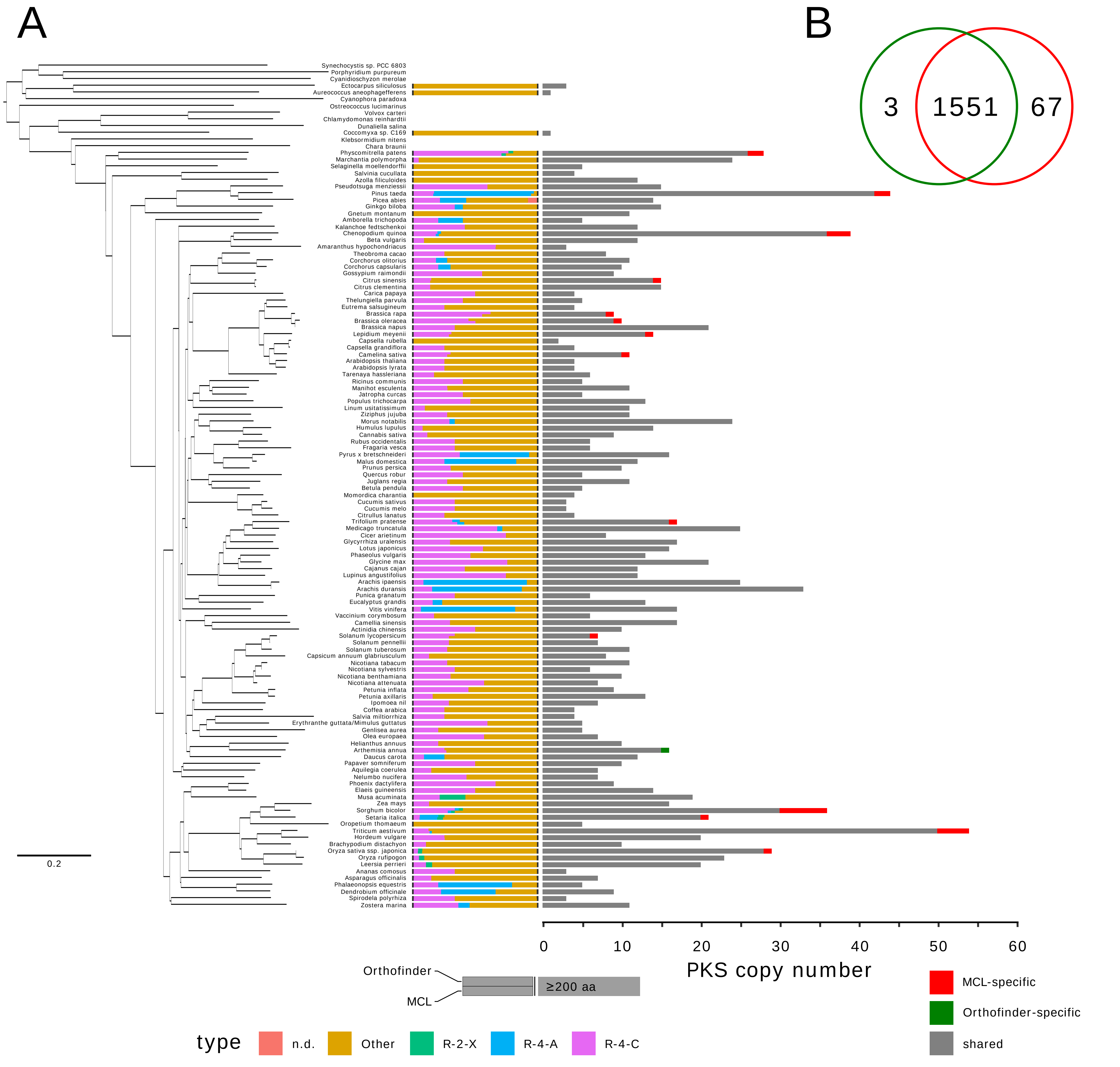
A
B
3
1551
67
0.2
Synechocystis sp. PCC 6803
Porphyridium purpureum
Cyanidioschyzon merolae
Ectocarpus siliculosus
Aureococcus aneophagefferens
Cyanophora paradoxa
Ostreococcus lucimarinus
Volvox carteri
Chlamydomonas reinhardtii
Dunaliella salina
Coccomyxa sp. C169
Klebsormidium nitens
Chara braunii
Physcomitrella patens
Marchantia polymorpha
Selaginella moellendorffii
Salvinia cucullata
Azolla filiculoides
Pseudotsuga menziessii
Pinus taeda
Picea abies
Ginkgo biloba
Gnetum montanum
Amborella trichopoda
Kalanchoe fedtschenkoi
Chenopodium quinoa
Beta vulgaris
Amaranthus hypochondriacus
Theobroma cacao
Corchorus olitorius
Corchorus capsularis
Gossypium raimondii
Citrus sinensis
Citrus clementina
Carica papaya
Thelungiella parvula
Eutrema salsugineum
Brassica rapa
Brassica oleracea
Brassica napus
Lepidium meyenii
Capsella rubella
Capsella grandiflora
Camelina sativa
Arabidopsis thaliana
Arabidopsis lyrata
Tarenaya hassleriana
Ricinus communis
Manihot esculenta
Jatropha curcas
Populus trichocarpa
Linum usitatissimum
Ziziphus jujuba
Morus notabilis
Humulus lupulus
Cannabis sativa
Rubus occidentalis
Fragaria vesca
Pyrus x bretschneideri
Malus domestica
Prunus persica
Quercus robur
Juglans regia
Betula pendula
Momordica charantia
Cucumis sativus
Cucumis melo
Citrullus lanatus
Trifolium pratense
Medicago truncatula
Cicer arietinum
Glycyrrhiza uralensis
Lotus japonicus
Phaseolus vulgaris
Glycine max
Cajanus cajan
Lupinus angustifolius
Arachis ipaensis
Arachis duranensis
Punica granatum
Eucalyptus grandis
Vitis vinifera
Vaccinium corymbosum
Camellia sinensis
Actinidia chinensis
Solanum lycopersicum
Solanum pennellii
Solanum tuberosum
Capsicum annuum glabriusculum
Nicotiana tabacum
Nicotiana sylvestris
Nicotiana benthamiana
Nicotiana attenuata
Petunia inflata
Petunia axillaris
Ipomoea nil
Coffea arabica
Salvia miltiorrhiza
Erythranthe guttata/Mimulus guttatus
Genlisea aurea
Olea europaea
Helianthus annuus
Arthemisia annua
Daucus carota
Papaver somniferum
Aquilegia coerulea
Nelumbo nucifera
Phoenix dactylifera
Elaeis guineensis
Musa acuminata
Zea mays
Sorghum bicolor
Setaria italica
Oropetium thomaeum
Triticum aestivum
Hordeum vulgare
Brachypodium distachyon
Oryza sativa ssp. japonica
Oryza rufipogon
Leersia perrieri
Ananas comosus
Asparagus officinalis
Phalaenopsis equestris
Dendrobium officinale
Spirodela polyrhiza
Zostera marina
0
10
20
30
40
50
60
PKS copy number
Orthofinder
MCL
≥200 aa
type
n.d.
Other
R-2-X
R-4-A
R-4-C
MCL-specific
Orthofinder-specific
shared

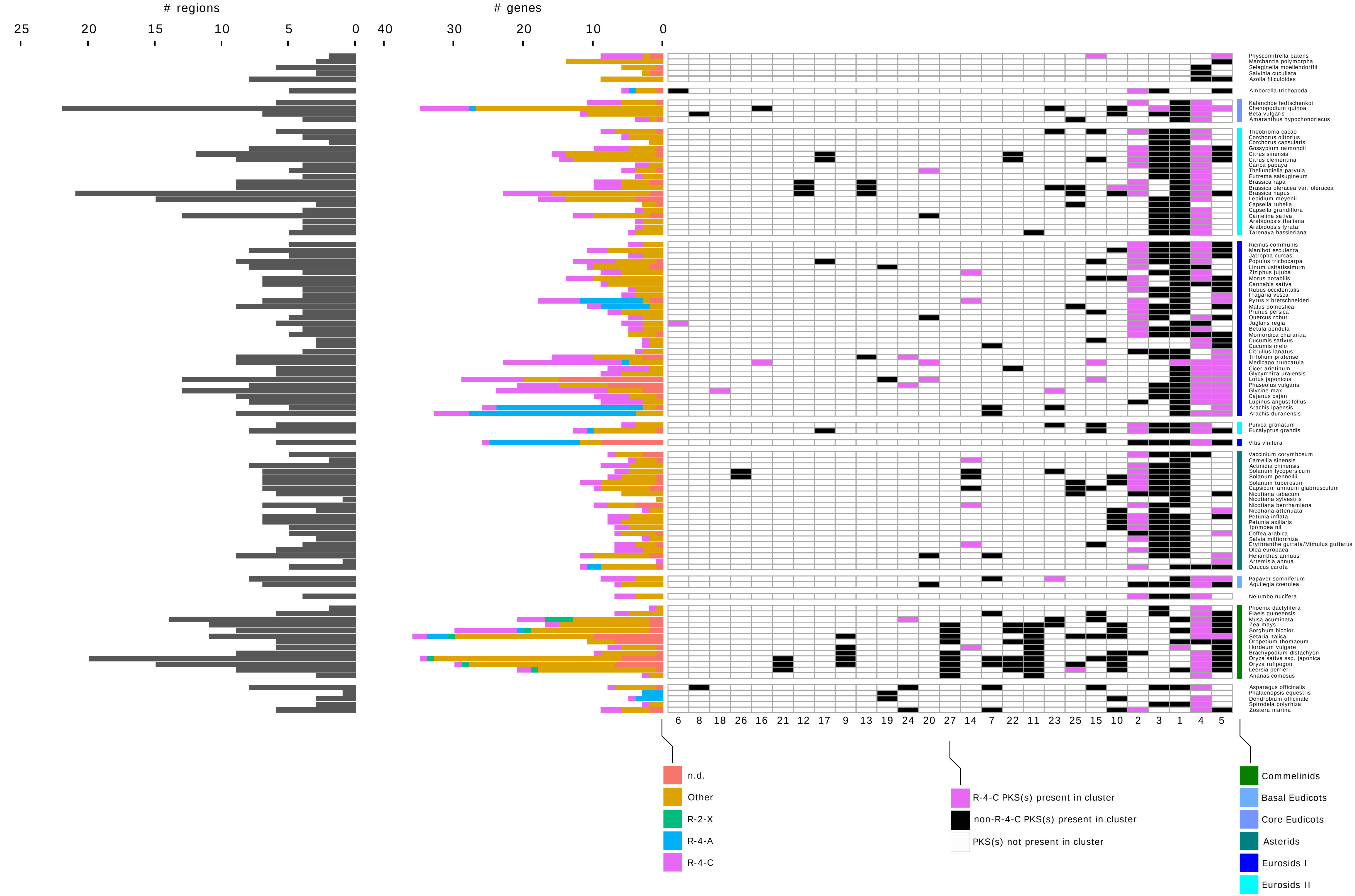

regions
25 20 15 10 5 0
genes
40 30 20 10 0
6 8 18 26 16 21 12 17 9 13 19 24 20 27 14 7 22 11 23 25 15 10 2 3 1 4 5
Physcomitrella patens
Marchantia polymorpha
Selaginella moellendorffii
Salvinia cucullata
Azolla filiculoides
Amborella trichopoda
Kalanchoe fedtschenkoi
Chenopodium quinoa
Beta vulgaris
Amaranthus hypochondriacus
Theobroma cacao
Corchorus olitorius
Corchorus capsularis
Gossypium raimondii
Citrus sinensis
Citrus clementina
Carica papaya
Thellungiella parvula
Eutrema salsugineum
Brassica rapa
Brassica oleracea var. oleracea
Brassica napus
Lepidium meyenii
Capsella rubella
Capsella grandiflora
Camelina sativa
Arabidopsis thaliana
Arabidopsis lyrata
Tarenaya hassleriana
Ricinus communis
Manihot esculenta
Jatropha curcas
Populus trichocarpa
Linum usitatissimum
Ziziphus jujuba
Morus notabilis
Cannabis sativa
Rubus occidentalis
Fragaria vesca
Pyrus x bretschneideri
Malus domestica
Prunus persica
Quercus robur
Juglans regia
Betula pendula
Momordica charantia
Cucumis sativus
Cucumis melo
Citrullus lanatus
Trifolium pratense
Medicago truncatula
Cicer arietinum
Glycyrrhiza uralensis
Lotus japonicus
Phaseolus vulgaris
Glycine max
Cajanus cajan
Lupinus angustifolius
Arachis ipaensis
Arachis duranensis
Punica granatum
Eucalyptus grandis
Vitis vinifera
Vaccinium corymbosum
Camellia sinensis
Actinidia chinensis
Solanum lycopersicum
Solanum pennellii
Solanum tuberosum
Capsicum annuum glabriusculum
Nicotiana tabacum
Nicotiana sylvestris
Nicotiana benthamiana
Nicotiana attenuata
Petunia inflata
Petunia axillaris
Ipomoea nil
Coffea arabica
Salvia miltiorrhiza
Erythranthe guttata/Mimulus guttatus
Olea europaea
Helianthus annuus
Artemisia annua
Daucus carota
Papaver somniferum
Aquilegia coerulea
Nelumbo nucifera
Phoenix dactylifera
Elaeis guineensis
Musa acuminata
Zea mays
Sorghum bicolor
Setaria italica
Oropetium thomaeum
Hordeum vulgare
Brachypodium distachyon
Oryza sativa ssp. japonica
Oryza rufipogon
Leersia perrieri
Ananas comosus
Asparagus officinalis
Phalaenopsis equestris
Dendrobium officinale
Spirodela polyrhiza
Zostera marina
n.d.
Other
R-2-X
R-4-A
R-4-C
R-4-C PKS(s) present in cluster
non-R-4-C PKS(s) present in cluster
PKS(s) not present in cluster
Commelinids
Basal Eudicots
Core Eudicots
Asterids
Eurosids I
Eurosids II

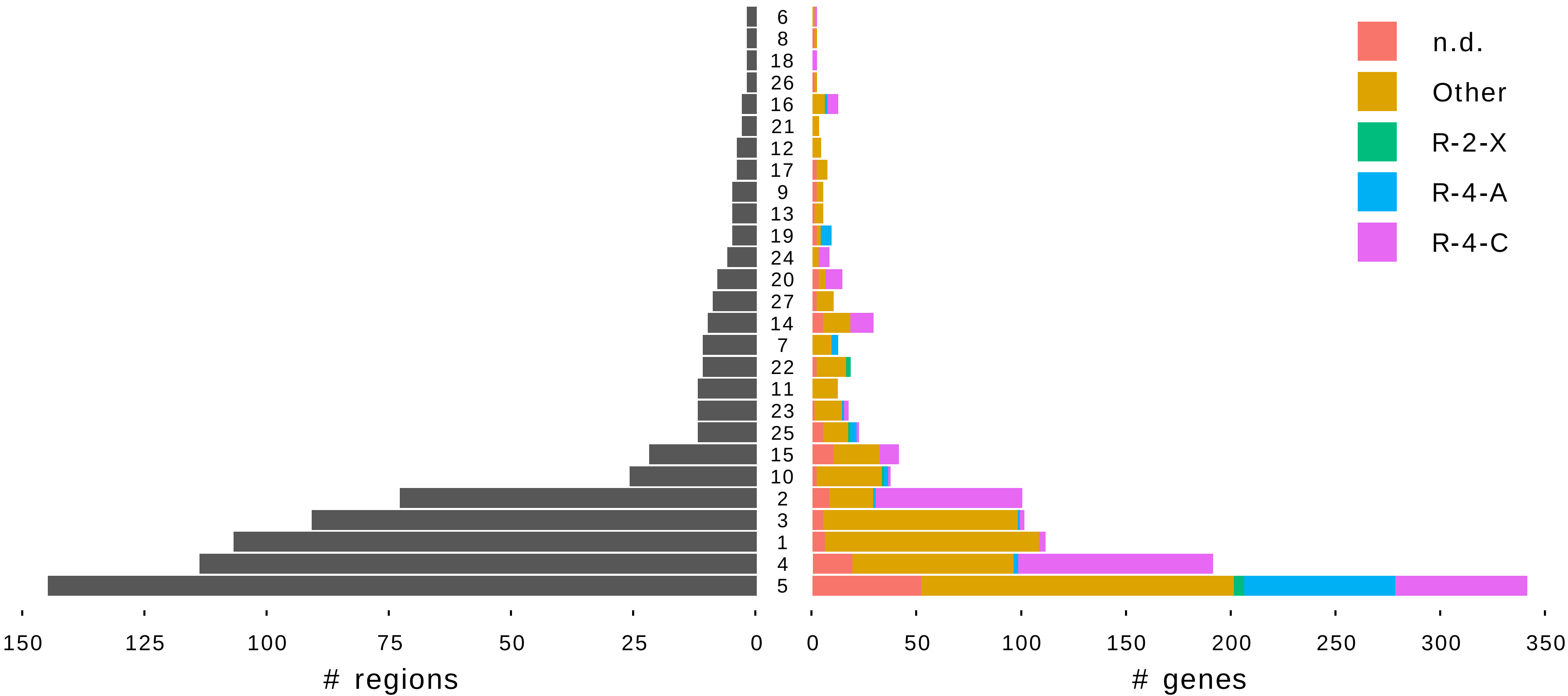
n.d.
Other
R-2-X
R-4-A
R-4-C
6
8
18
26
16
21
12
17
9
13
19
24
20
27
14
7
22
11
23
25
15
10
2
3
1
4
5
150
125
100
75
50
25
0
0
50
100
150
200
250
300
350
regions
genes

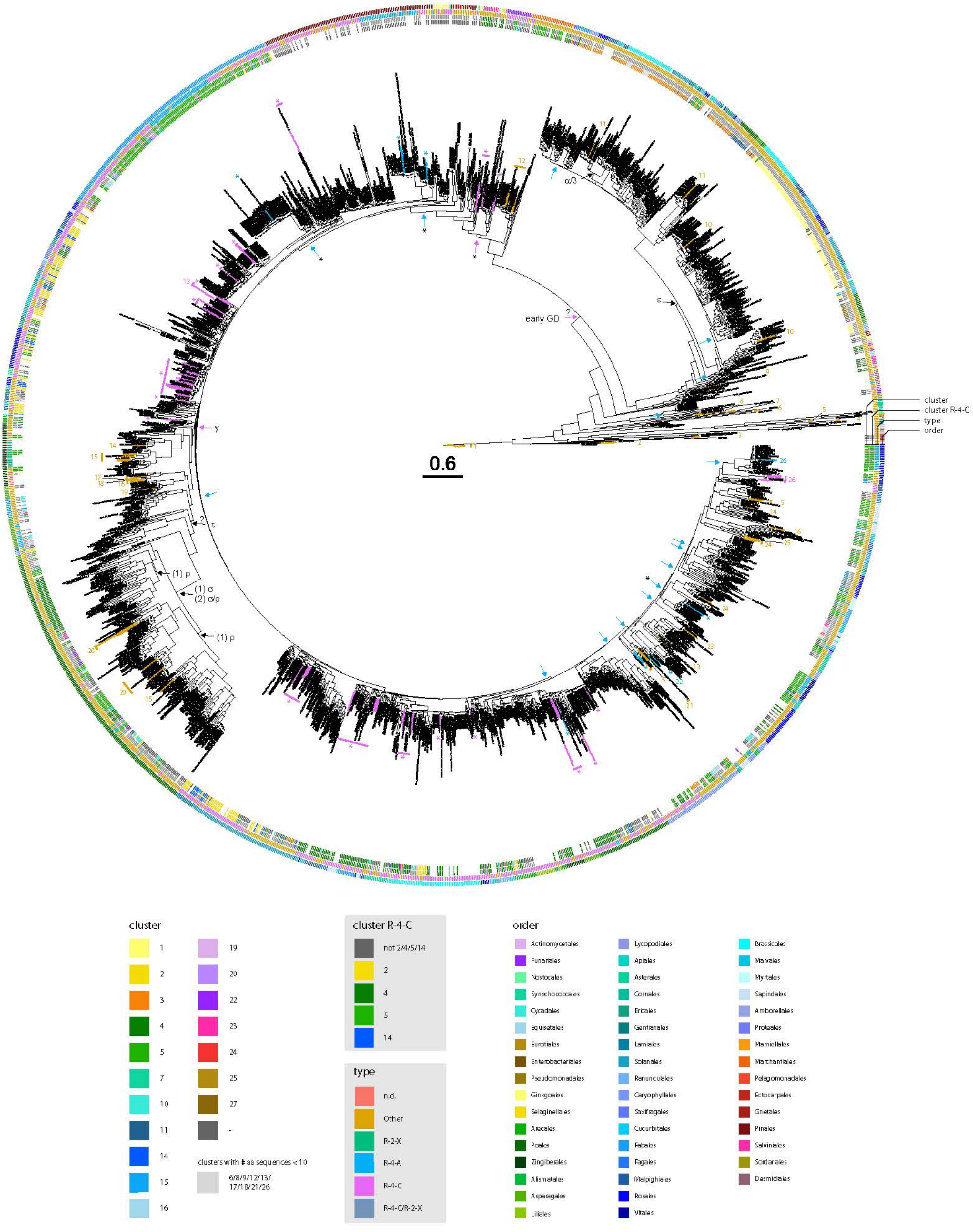

0.6
early GD
α/β
ε
γ
τ
(1) ρ
(1) σ
(2) σ/ρ
(1) ρ
cluster
cluster R-4-C
type
order
cluster
1
2
3
4
5
7
10
11
14
15
16
19
20
22
23
24
25
27
-
clusters with # aa sequences < 10
6/8/9/12/13/17/18/21/26
cluster R-4-C
not 2/4/5/14
2
4
5
14
type
n.d.
Other
R-2-X
R-4-A
R-4-C
R-4-C/R-2-X
order
Actinomycetales
Funariales
Nostocales
Synechococcales
Cycadales
Equisetales
Eurotiales
Enterobacteriales
Pseudomonadales
Ginkgoales
Selaginellales
Arecales
Poales
Zingiberales
Alismatales
Asparagales
Liliales
Lycopodiales
Apiales
Asterales
Cornales
Ericales
Gentianales
Lamiales
Solanales
Ranunculales
Caryophyllales
Saxifragales
Cucurbitales
Fabales
Fagales
Malpighiales
Rosales
Vitales
Brassicales
Malvales
Myrtales
Sapindales
Amborellales
Proteales
Mamiellales
Marchantiales
Pelagomonadales
Ectocarpales
Gnetales
Pinales
Salviniales
Sordariales
Desmidiales

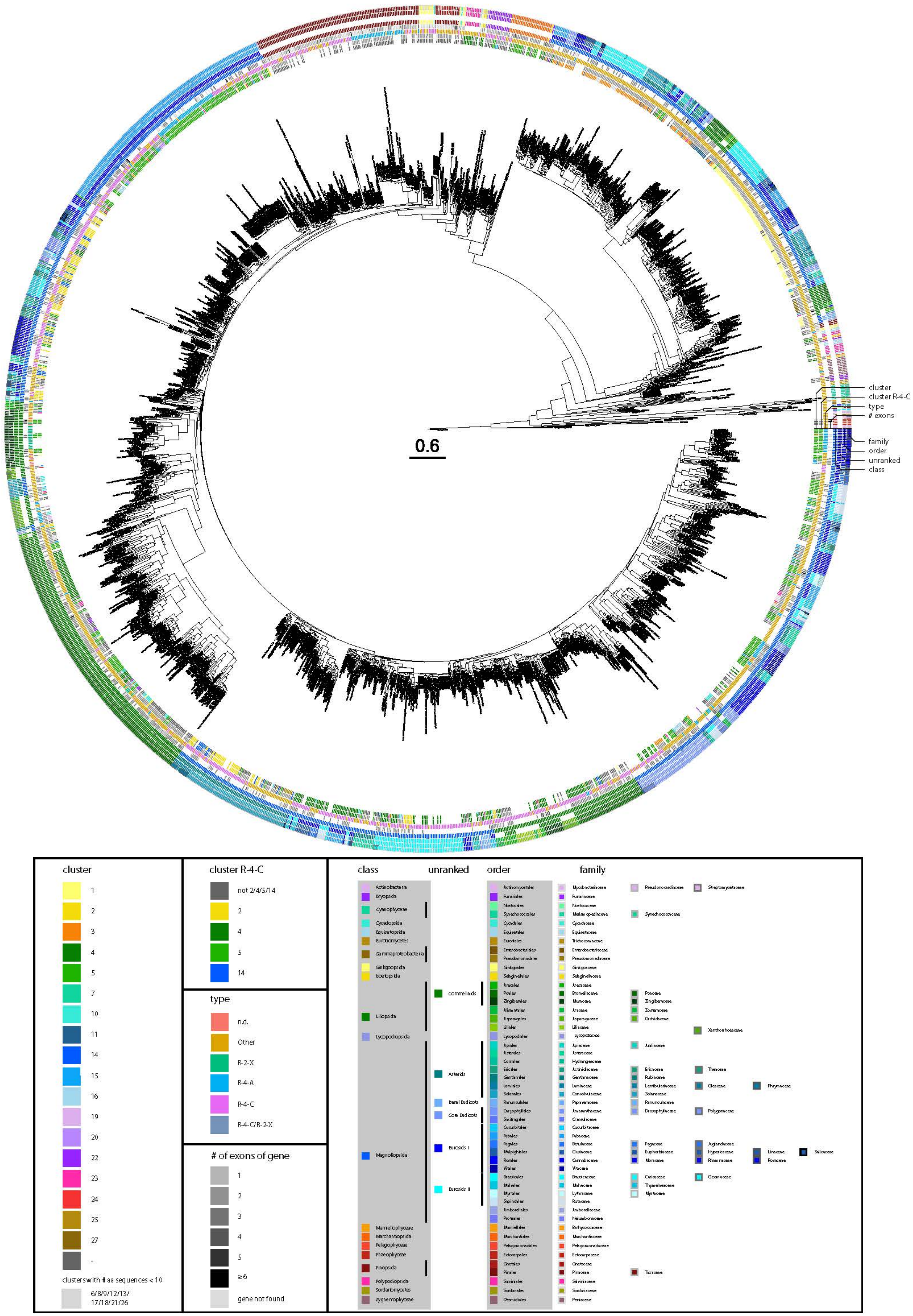
cluster
cluster R-4-C
type
exons
family
order
unranked
class
0.6
cluster
1
2
3
4
5
7
10
11
14
15
16
19
20
22
23
24
25
27
-
clusters with # aa sequences < 10
6/8/9/12/13/17/18/21/26
cluster R-4-C
not 2/4/5/14
2
4
5
14
type
n.d.
Other
R-2-X
R-4-A
R-4-C
R-4-C/R-2-X
# of exons of gene
1
2
3
4
5
≥6
gene not found
class
Actinobacteria
Bryopsida
Cyanophyceae
Cycadopsida
Equisetopsida
Eurotiomycetes
Gammaproteobacteria
Ginkgoopsida
Isoetopsida
Liliopsida
Lycopodiopsida
Magnoliopsida
Mamiellophyceae
Marchantiopsida
Pelagophyceae
Phaeophyceae
Pinopsida
Polypodiopsida
Sordariomycetes
Zygnemophyceae
unranked
Commelinids
Asterids
Basal Eudicots
Core Eudicots
Eurosids I
Eurosids II
order
Actinomycetales
Funariales
Nostocales
Synechococcales
Cycadales
Equisetales
Eurotiales
Enterobacteriales
Pseudomonadales
Ginkgoales
Selaginellales
Arecales
Poales
Zingiberales
Alismatales
Asparagales
Liliales
Lycopodiales
Apiales
Asterales
Cornales
Ericales
Gentianales
Lamiales
Solanales
Ranunculales
Caryophyllales
Saxifragales
Cucurbitales
Fabales
Fagales
Malpighiales
Rosales
Vitales
Brassicales
Malvales
Myrtales
Sapindales
Amborellales
Proteales
Mamiellales
Marchantiales
Pelagomonadales
Ectocarpales
Gnetales
Pinales
Salviniales
Sordariales
Desmidiales
family
Mycobacteriaceae
Pseudonocardiaceae
Streptomycetaceae
Funariaceae
Nostocaceae
Merismopediaceae
Synechococcaceae
Cycadaceae
Equisetaceae
Trichocomaceae
Enterobacteriaceae
Pseudomonadaceae
Ginkgoaceae
Selaginellaceae
Arecaceae
Bromeliaceae
Poaceae
Musaceae
Zingiberaceae
Araceae
Zosteraceae
Asparagaceae
Orchidaceae
Liliaceae
Xanthorrhoeaceae
Lycopodiaceae
Apiaceae
Araliaceae
Asteraceae
Hydrangeaceae
Actinidiaceae
Ericaceae
Theaceae
Gentianaceae
Rubiaceae
Lamiaceae
Lentibulariaceae
Oleaceae
Phrymaceae
Convolvulaceae
Solanaceae
Papaveraceae
Ranunculaceae
Amaranthaceae
Drosophyllaceae
Polygonaceae
Crassulaceae
Cucurbitaceae
Fabaceae
Betulaceae
Fagaceae
Juglandaceae
Clusiaceae
Euphorbiaceae
Hypericaceae
Linaceae
Salicaceae
Cannabaceae
Moraceae
Rhamnaceae
Rosaceae
Vitaceae
Brassicaceae
Caricaceae
Cleomaceae
Malvaceae
Thymelaeaceae
Lythraceae
Myrtaceae
Rutaceae
Amborellaceae
Nelumbonaceae
Bathycoccaceae
Marchantiaceae
Pelagomonadaceae
Ectocarpaceae
Gnetaceae
Pinaceae
Taxaceae
Salviniaceae
Sordariaceae
Peniaceae

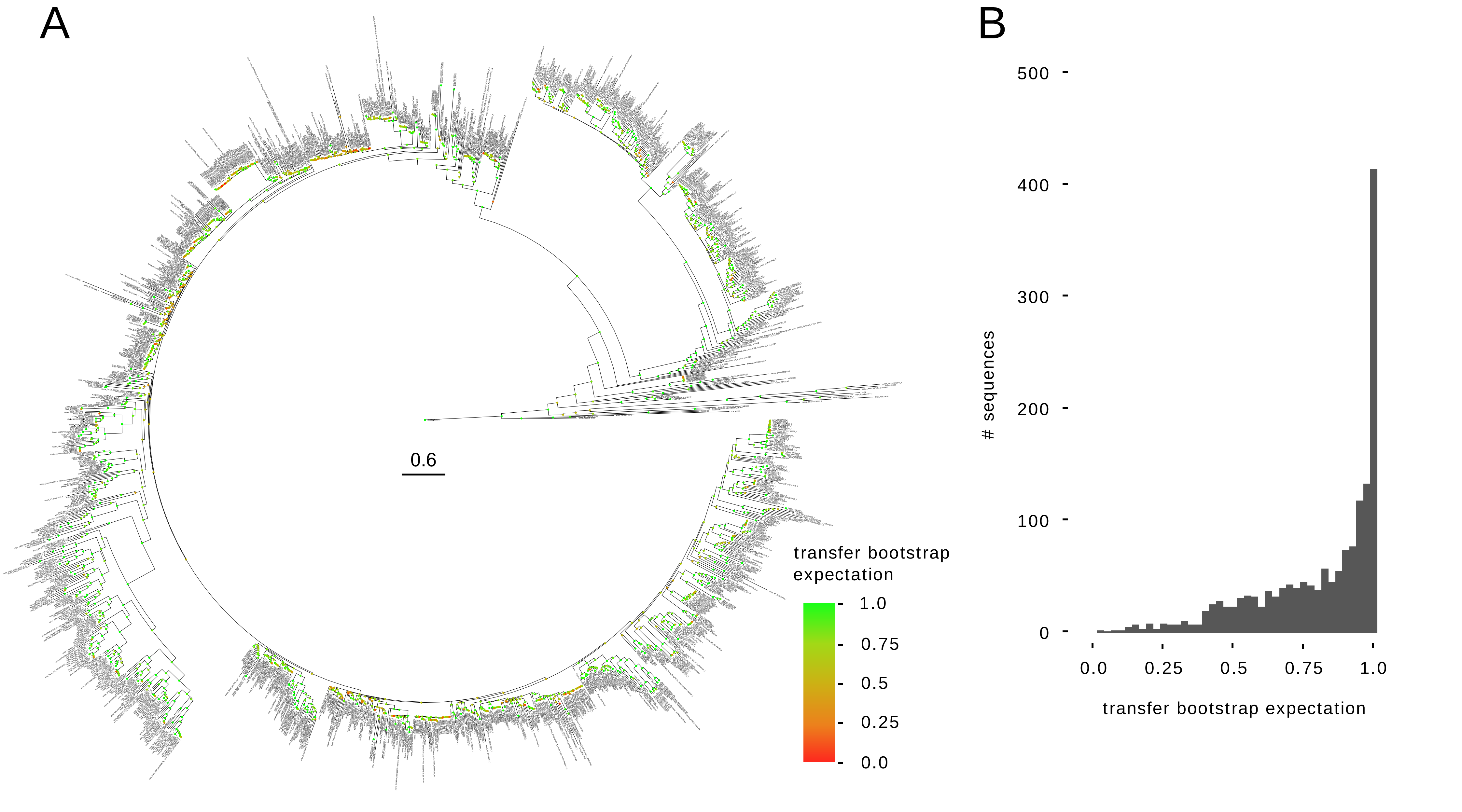

A
B
0.6
transfer bootstrap expectation
1.0
0.75
0.5
0.25
0.0
sequences
500
400
300
200
100
0
0.0
0.25
0.5
0.75
1.0
transfer bootstrap expectation

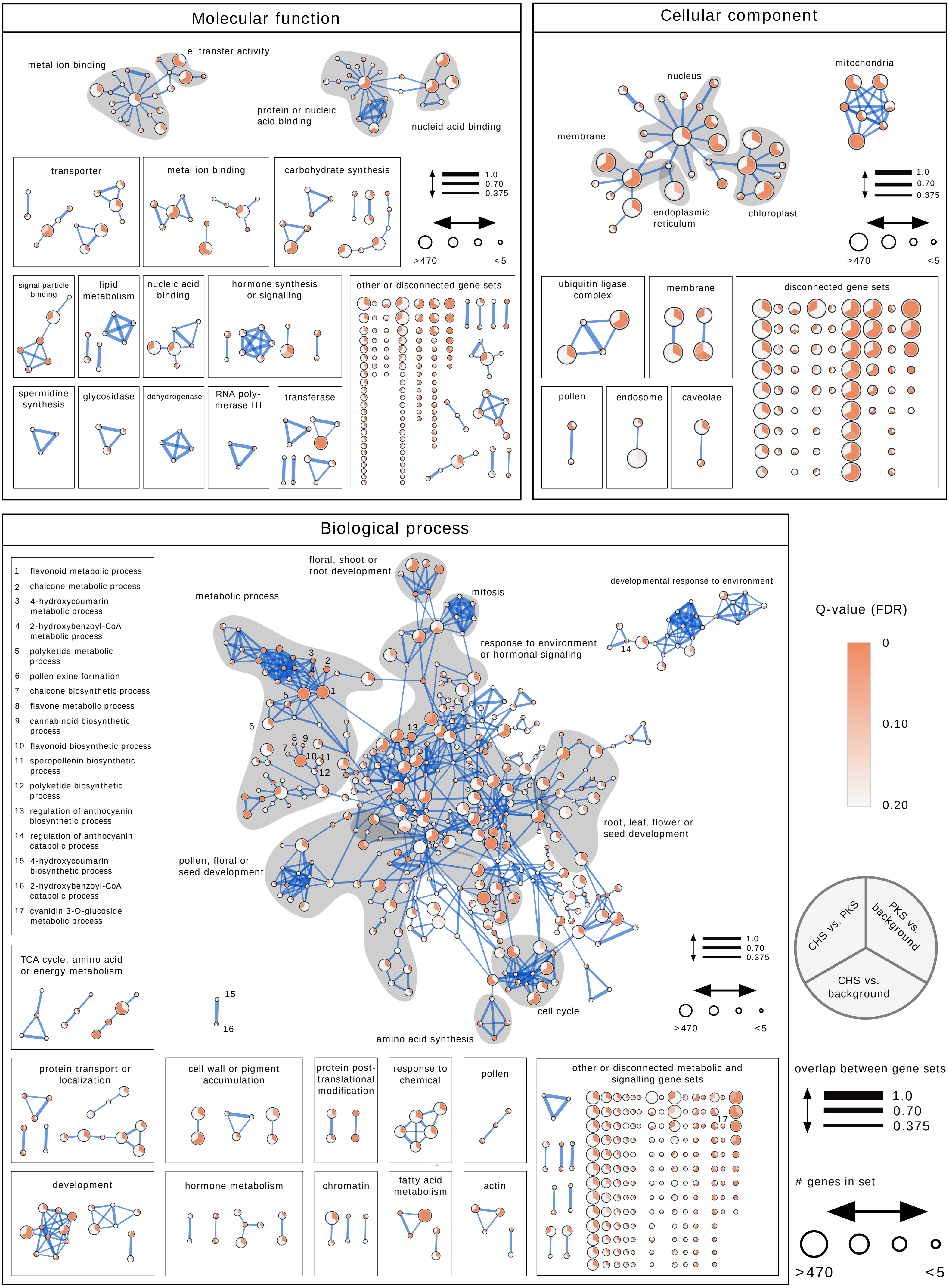

Molecular function
metal ion binding
e- transfer activity
protein or nucleic acid binding
nucleid acid binding
transporter
metal ion binding
carbohydrate synthesis
1.0
0.70
0.375
>470
<5
signal particle binding
lipid metabolism
nucleic acid binding
hormone synthesis or signalling
other or disconnected gene sets
spermidine synthesis
glycosidase
dehydrogenase
RNA polymerase III
transferase
Cellular component
nucleus
mitochondria
membrane
endoplasmic reticulum
chloroplast
1.0
0.70
0.375
>470
<5
ubiquitin ligase complex
membrane
disconnected gene sets
pollen
endosome
caveolae
Biological process
1 flavonoid metabolic process
2 chalcone metabolic process
3 4-hydroxycoumarin metabolic process
4 2-hydroxybenzoyl-CoA metabolic process
5 polyketide metabolic process
6 pollen exine formation
7 chalcone biosynthetic process
8 flavone metabolic process
9 cannabinoid biosynthetic process
10 flavonoid biosynthetic process
11 sporopollenin biosynthetic process
12 polyketide biosynthetic process
13 regulation of anthocyanin biosynthetic process
14 regulation of anthocyanin catabolic process
15 4-hydroxycoumarin biosynthetic process
16 2-hydroxybenzoyl-CoA catabolic process
17 cyanidin 3-O-glucoside metabolic process
floral, shoot or root development
metabolic process
mitosis
developmental response to environment
response to environment or hormonal signaling
root, leaf, flower or seed development
pollen, floral or seed development
cell cycle
amino acid synthesis
TCA cycle, amino acid or energy metabolism
1.0
0.70
0.375
>470
<5
protein transport or localization
cell wall or pigment accumulation
protein post-translational modification
response to chemical
pollen
other or disconnected metabolic and signalling gene sets
development
hormone metabolism
chromatin
fatty acid metabolism
actin
Q-value (FDR)
0
0.10
0.20
CHS vs. PKS
PKS vs. background
CHS vs. background
overlap between gene sets
1.0
0.70
0.375
genes in set
>470
<5

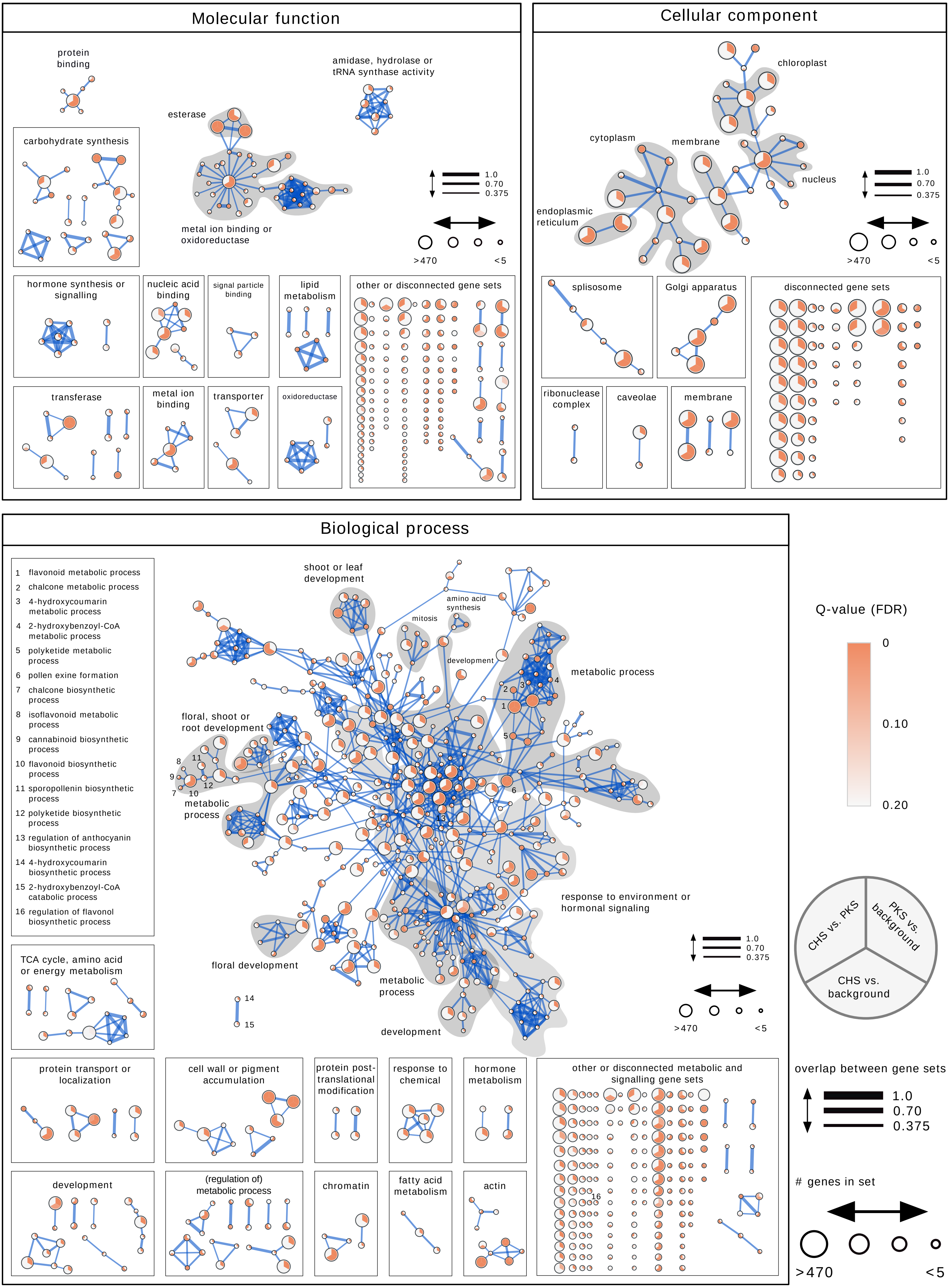

Molecular function
protein binding
amidase, hydrolase or tRNA synthase activity
esterase
carbohydrate synthesis
metal ion binding or oxidoreductase
1.0
0.70
0.375
>470
<5
hormone synthesis or signalling
nucleic acid binding
signal particle binding
lipid metabolism
other or disconnected gene sets
transferase
metal ion binding
transporter
oxidoreductase
Cellular component
chloroplast
cytoplasm
membrane
nucleus
endoplasmic reticulum
1.0
0.70
0.375
>470
<5
splisosome
Golgi apparatus
disconnected gene sets
ribonuclease complex
caveolae
membrane
Biological process
1 flavonoid metabolic process
2 chalcone metabolic process
3 4-hydroxycoumarin metabolic process
4 2-hydroxybenzoyl-CoA metabolic process
5 polyketide metabolic process
6 pollen exine formation
7 chalcone biosynthetic process
8 isoflavonoid metabolic process
9 cannabinoid biosynthetic process
10 flavonoid biosynthetic process
11 sporopollenin biosynthetic process
12 polyketide biosynthetic process
13 regulation of anthocyanin biosynthetic process
14 4-hydroxycoumarin biosynthetic process
15 2-hydroxybenzoyl-CoA catabolic process
16 regulation of flavonol biosynthetic process
shoot or leaf development
amino acid synthesis
mitosis
development
metabolic process
floral, shoot or root development
metabolic process
response to environment or hormonal signaling
floral development
metabolic process
development
1.0
0.70
0.375
>470
<5
TCA cycle, amino acid or energy metabolism
protein transport or localization
cell wall or pigment accumulation
protein post-translational modification
response to chemical
hormone metabolism
other or disconnected metabolic and signalling gene sets
development
(regulation of) metabolic process
chromatin
fatty acid metabolism
actin
Q-value (FDR)
0
0.10
0.20
CHS vs. PKS
PKS vs. background
CHS vs. background
overlap between gene sets
1.0
0.70
0.375
genes in set
>470
<5

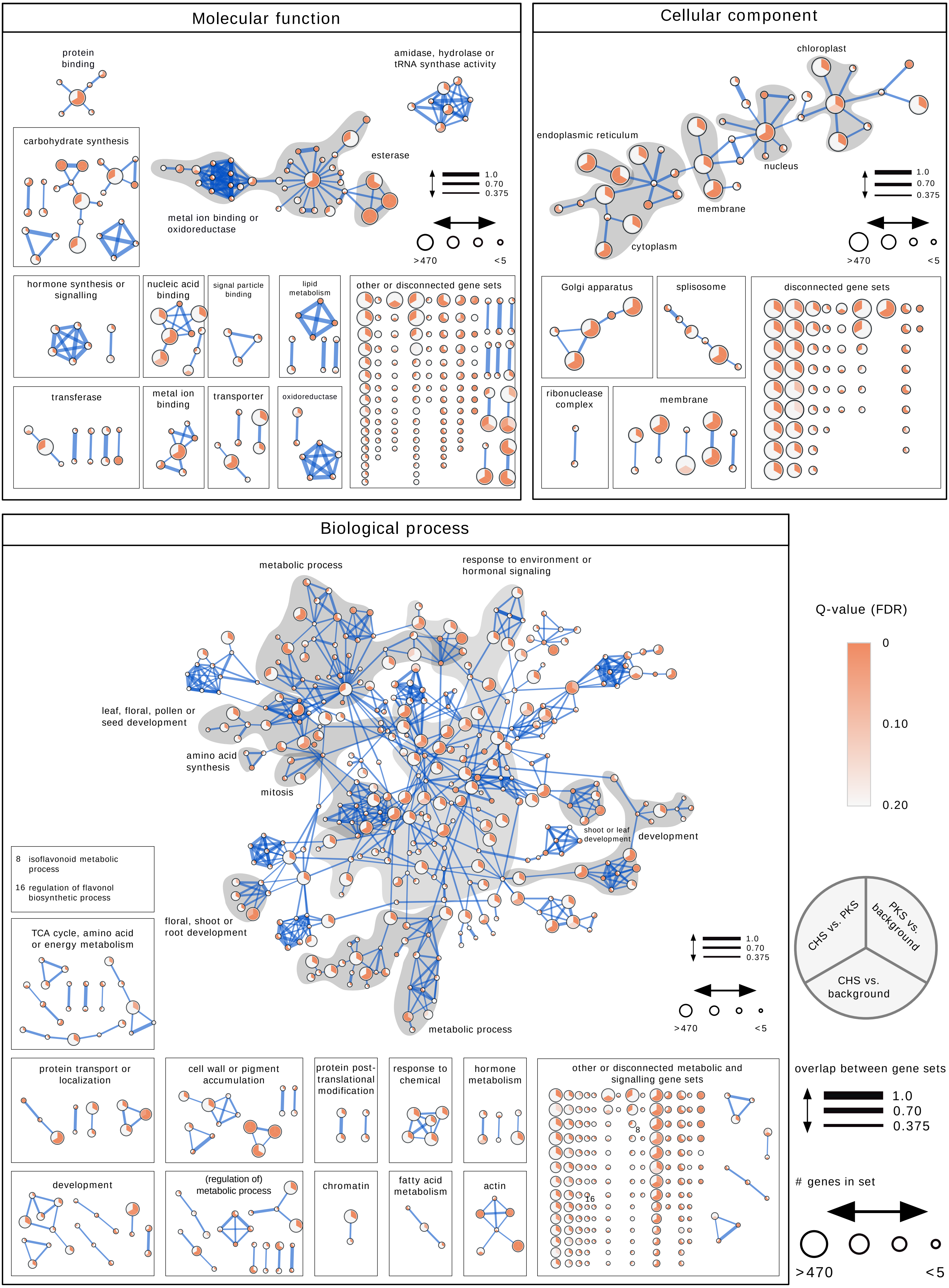
Molecular function
protein binding
amidase, hydrolase or tRNA synthase activity
carbohydrate synthesis
esterase
metal ion binding or oxidoreductase
1.0
0.70
0.375
>470
<5
hormone synthesis or signalling
nucleic acid binding
signal particle binding
lipid metabolism
other or disconnected gene sets
transferase
metal ion binding
transporter
oxidoreductase
Cellular component
chloroplast
endoplasmic reticulum
nucleus
membrane
cytoplasm
Golgi apparatus
splisosome
disconnected gene sets
ribonuclease complex
membrane
Biological process
metabolic process
response to environment or hormonal signaling
leaf, floral, pollen or seed development
amino acid synthesis
mitosis
shoot or leaf development
development
8 isoflavonoid metabolic process
16 regulation of flavonol biosynthetic process
floral, shoot or root development
TCA cycle, amino acid or energy metabolism
metabolic process
protein transport or localization
cell wall or pigment accumulation
protein post-translational modification
response to chemical
hormone metabolism
other or disconnected metabolic and signalling gene sets
development
(regulation of) metabolic process
chromatin
fatty acid metabolism
actin
Q-value (FDR)
0
0.10
0.20
CHS vs. PKS
PKS vs. background
CHS vs. background
overlap between gene sets
genes in set

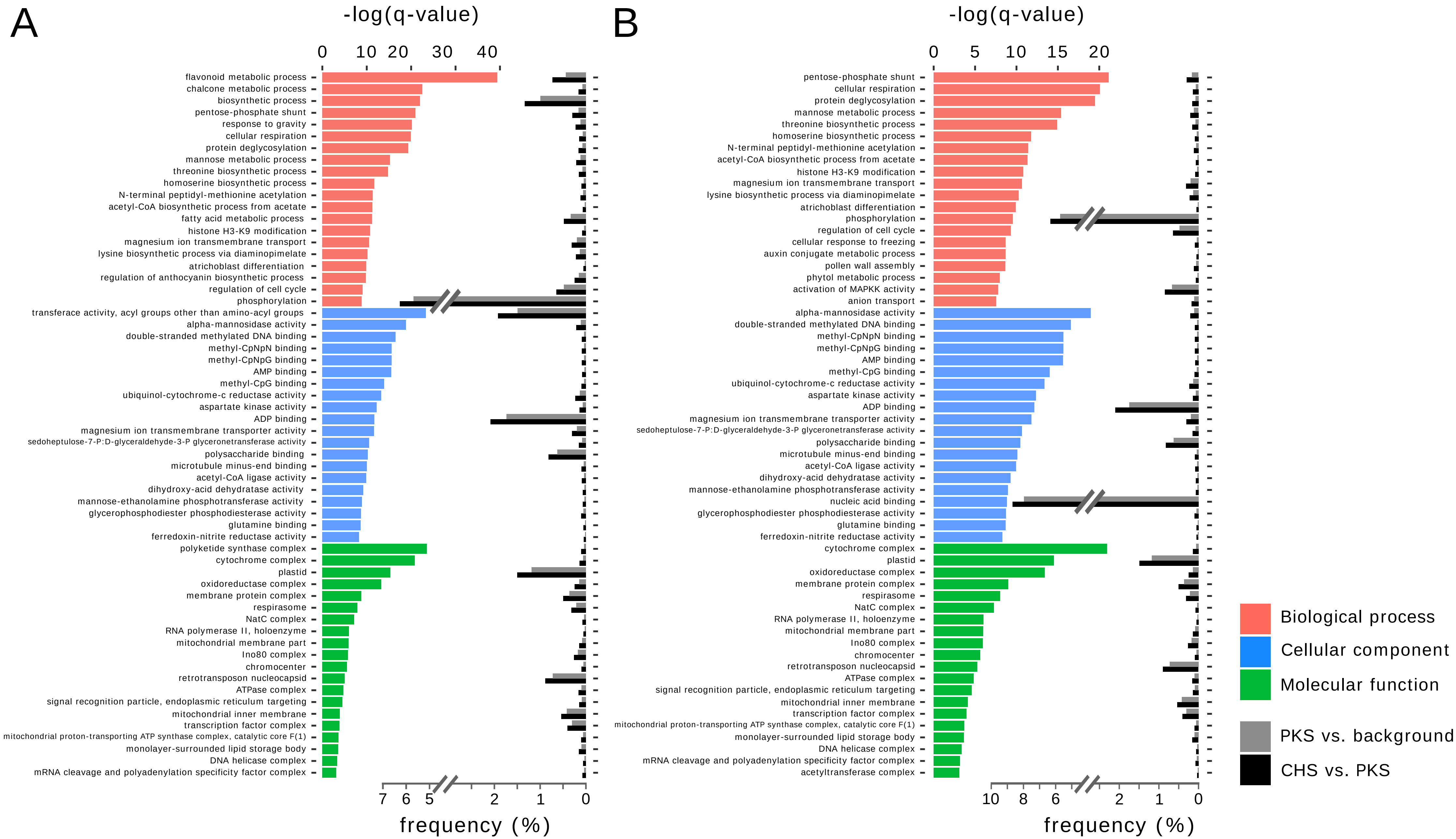
A
-log(q-value)
0
10
20
30
40
flavonoid metabolic process
chalcone metabolic process
biosynthetic process
pentose-phosphate shunt
response to gravity
cellular respiration
protein deglycosylation
mannose metabolic process
threonine biosynthetic process
homoserine biosynthetic process
N-terminal peptidyl-methionine acetylation
acetyl-CoA biosynthetic process from acetate
fatty acid metabolic process
histone H3-K9 modification
magnesium ion transmembrane transport
lysine biosynthetic process via diaminopimelate
atrichoblast differentiation
regulation of anthocyanin biosynthetic process
regulation of cell cycle
phosphorylation
transferace activity, acyl groups other than amino-acyl groups
alpha-mannosidase activity
double-stranded methylated DNA binding
methyl-CpNpN binding
methyl-CpNpG binding
AMP binding
methyl-CpG binding
ubiquinol-cytochrome-c reductase activity
aspartate kinase activity
ADP binding
magnesium ion transmembrane transporter activity
sedoheptulose-7-P:D-glyceraldehyde-3-P glyceronetransferase activity
polysaccharide binding
microtubule minus-end binding
acetyl-CoA ligase activity
dihydroxy-acid dehydratase activity
mannose-ethanolamine phosphotransferase activity
glycerophosphodiester phosphodiesterase activity
glutamine binding
ferredoxin-nitrite reductase activity
polyketide synthase complex
cytochrome complex
plastid
oxidoreductase complex
membrane protein complex
respirasome
NatC complex
RNA polymerase II, holoenzyme
mitochondrial membrane part
Ino80 complex
chromocenter
retrotransposon nucleocapsid
ATPase complex
signal recognition particle, endoplasmic reticulum targeting
mitochondrial inner membrane
transcription factor complex
mitochondrial proton-transporting ATP synthase complex, catalytic core F(1)
monolayer-surrounded lipid storage body
DNA helicase complex
mRNA cleavage and polyadenylation specificity factor complex
7
6
5
2
1
0
frequency (%)
B
-log(q-value)
0
5
10
15
20
pentose-phosphate shunt
cellular respiration
protein deglycosylation
mannose metabolic process
threonine biosynthetic process
homoserine biosynthetic process
N-terminal peptidyl-methionine acetylation
acetyl-CoA biosynthetic process from acetate
histone H3-K9 modification
magnesium ion transmembrane transport
lysine biosynthetic process via diaminopimelate
atrichoblast differentiation
phosphorylation
regulation of cell cycle
cellular response to freezing
auxin conjugate metabolic process
pollen wall assembly
phytol metabolic process
activation of MAPKK activity
anion transport
alpha-mannosidase activity
double-stranded methylated DNA binding
methyl-CpNpN binding
methyl-CpNpG binding
AMP binding
methyl-CpG binding
ubiquinol-cytochrome-c reductase activity
aspartate kinase activity
ADP binding
magnesium ion transmembrane transporter activity
sedoheptulose-7-P:D-glyceraldehyde-3-P glyceronetransferase activity
polysaccharide binding
microtubule minus-end binding
acetyl-CoA ligase activity
dihydroxy-acid dehydratase activity
mannose-ethanolamine phosphotransferase activity
nucleic acid binding
glycerophosphodiester phosphodiesterase activity
glutamine binding
ferredoxin-nitrite reductase activity
cytochrome complex
plastid
oxidoreductase complex
membrane protein complex
respirasome
NatC complex
RNA polymerase II, holoenzyme
mitochondrial membrane part
Ino80 complex
chromocenter
retrotransposon nucleocapsid
ATPase complex
signal recognition particle, endoplasmic reticulum targeting
mitochondrial inner membrane
transcription factor complex
mitochondrial proton-transporting ATP synthase complex, catalytic core F(1)
monolayer-surrounded lipid storage body
DNA helicase complex
mRNA cleavage and polyadenylation specificity factor complex
acetyltransferase complex
10
8
6
2
1
0
frequency (%)
Biological process
Cellular component
Molecular function
PKS vs. background
CHS vs. PKS

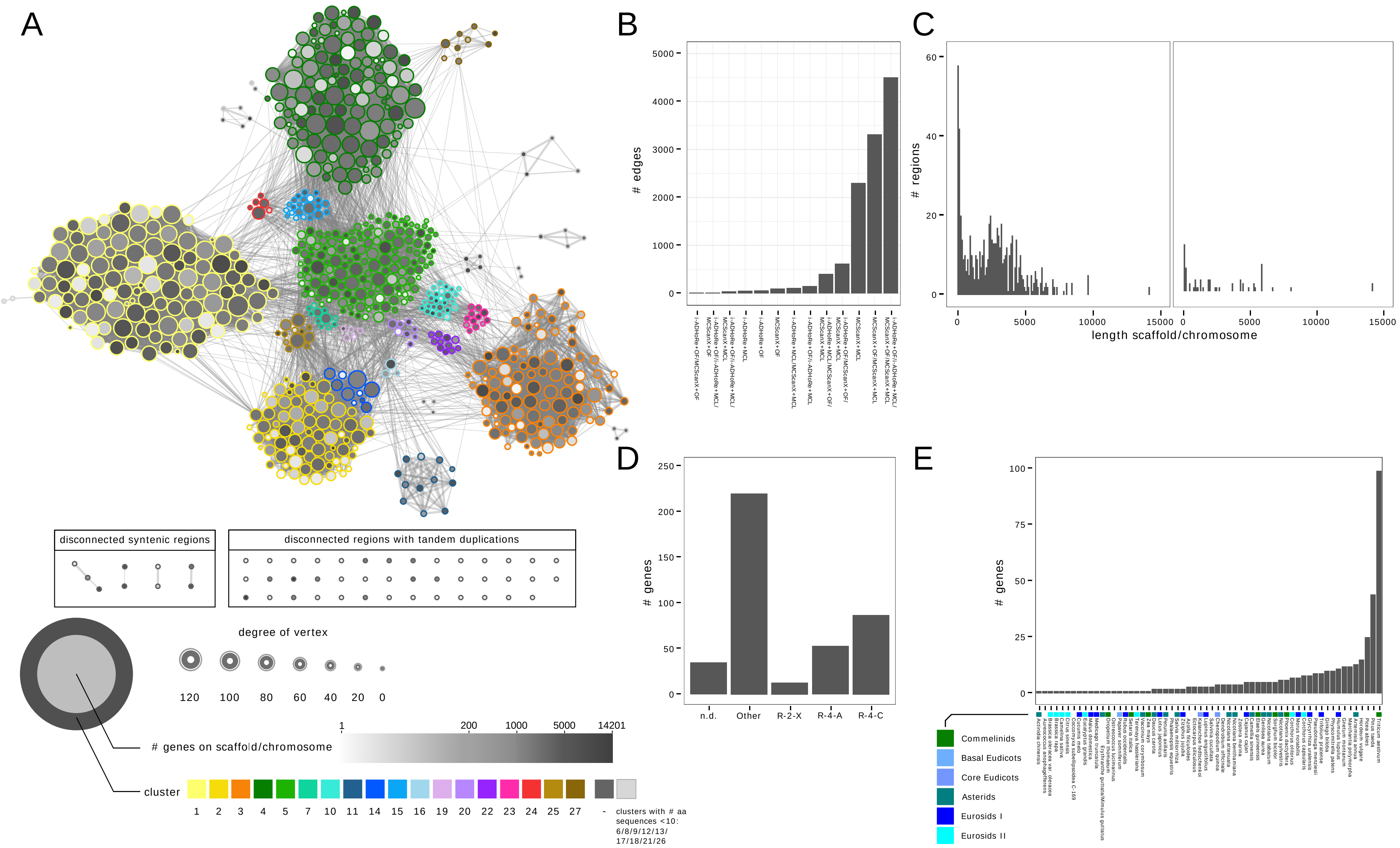

A
B
C
D
E
disconnected syntenic regions
disconnected regions with tandem duplications
degree of vertex
120
100
80
60
40
20
0
genes on scaffold/chromosome
1
200
1000
5000
14201
cluster
1
2
3
4
5
7
10
11
14
15
16
19
20
22
23
24
25
27
-
clusters with # aa sequences <10: 6/8/9/12/13/17/18/21/26
edges
0
1000
2000
3000
4000
5000
i-ADHoRe+OF/MCScanX+OF
MCScanX+OF
i-ADHoRe+OF/i-ADHoRe+MCL/
i-ADHoRe+OF/i-ADHoRe+MCL/
MCScanX+MCL
i-ADHoRe+MCL
i-ADHoRe+OF
MCScanX+OF
i-ADHoRe+MCL/MCScanX+MCL
i-ADHoRe+OF/i-ADHoRe+MCL
i-ADHoRe+MCL/MCScanX+OF/
MCScanX+MCL
i-ADHoRe+OF/MCScanX+OF/
MCScanX+MCL
MCScanX+OF/MCScanX+MCL
i-ADHoRe+OF/i-ADHoRe+MCL/
MCScanX+OF/MCScanX+MCL
regions
0
20
40
60
0
5000
10000
15000
length scaffold/chromosome
genes
0
50
100
150
200
250
n.d.
Other
R-2-X
R-4-A
R-4-C
0
25
50
75
100
Commelinids
Basal Eudicots
Core Eudicots
Asterids
Eurosids I
Eurosids II

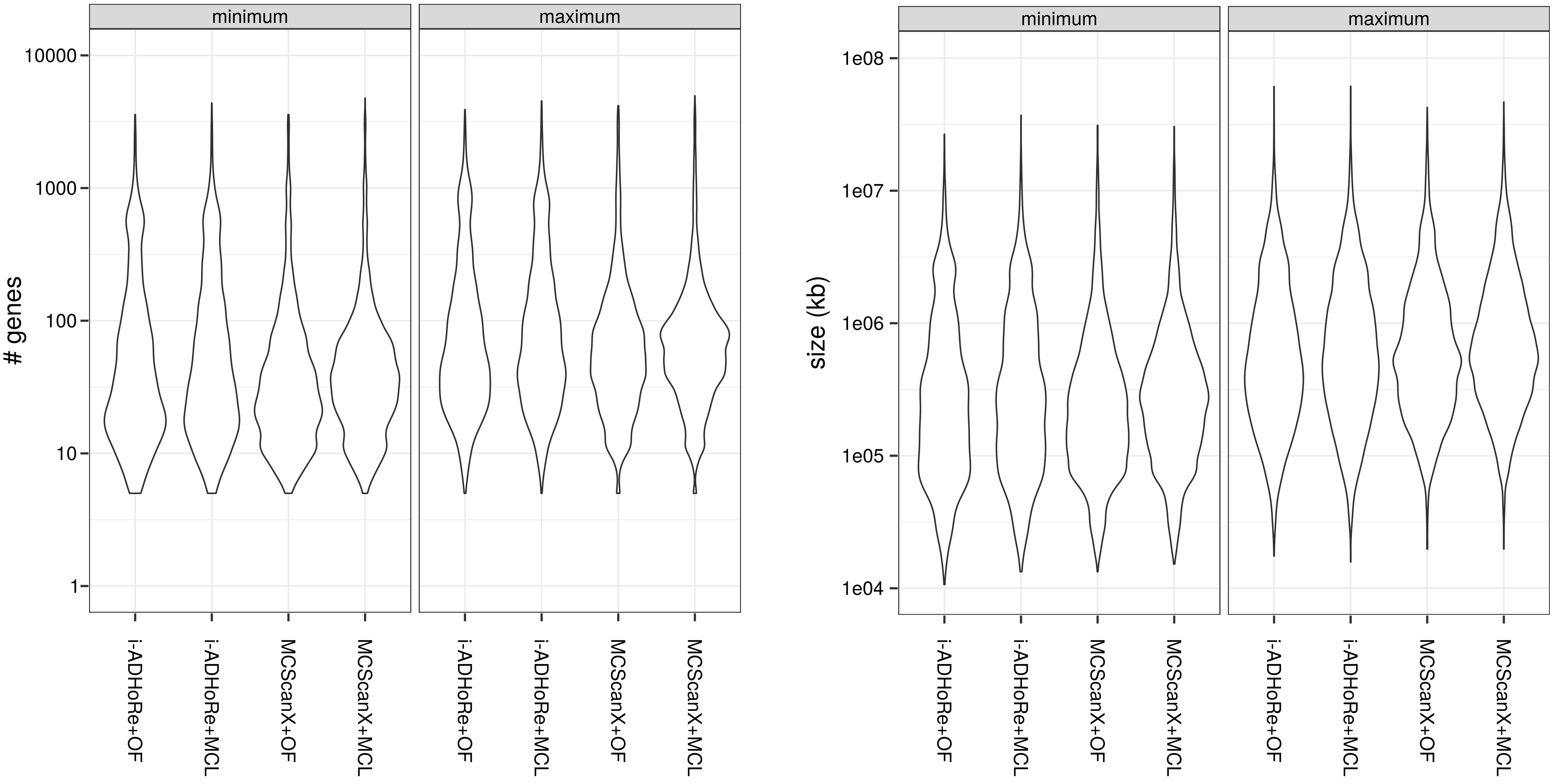

minimum
maximum
genes
10000
1000
100
10
1
i-ADHoRe+OF
i-ADHoRe+MCL
MCScanX+OF
MCScanX+MCL
size (kb)
1e08
1e07
1e06
1e05
1e04

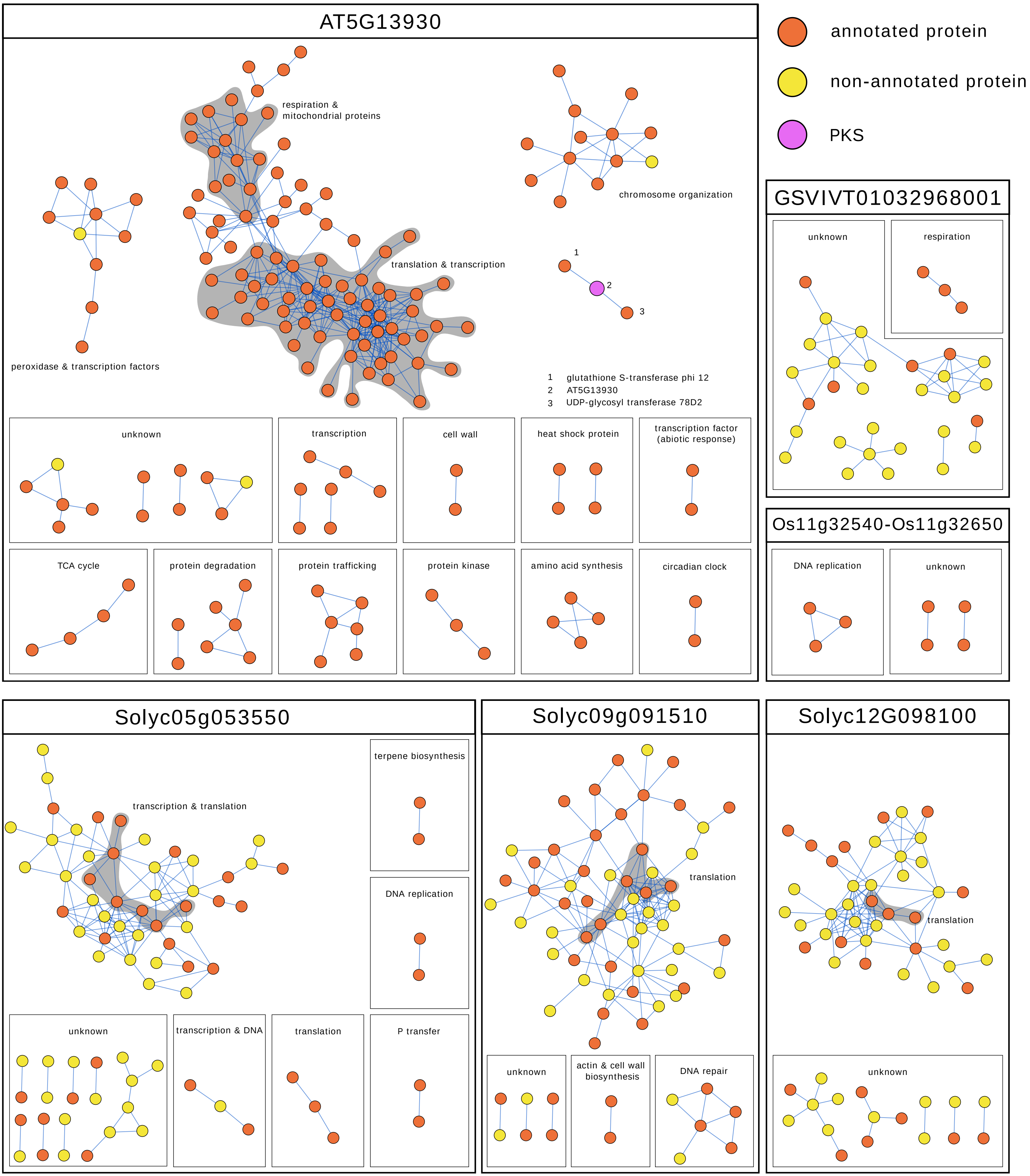
annotated protein
non-annotated protein
PKS
AT5G13930
respiration & mitochondrial proteins
chromosome organization
peroxidase & transcription factors
translation & transcription
1
2
3
1 glutathione S-transferase phi 12
2 AT5G13930
3 UDP-glycosyl transferase 78D2
unknown
transcription
cell wall
heat shock protein
transcription factor (abiotic response)
TCA cycle
protein degradation
protein trafficking
protein kinase
amino acid synthesis
circadian clock
GSVIVT01032968001
unknown
respiration
Os11g32540-Os11g32650
DNA replication
unknown
Solyc05g053550
transcription & translation
terpene biosynthesis
DNA replication
unknown
transcription & DNA
translation
P transfer
Solyc09g091510
translation
unknown
actin & cell wall biosynthesis
DNA repair
Solyc12G098100
translation
unknown

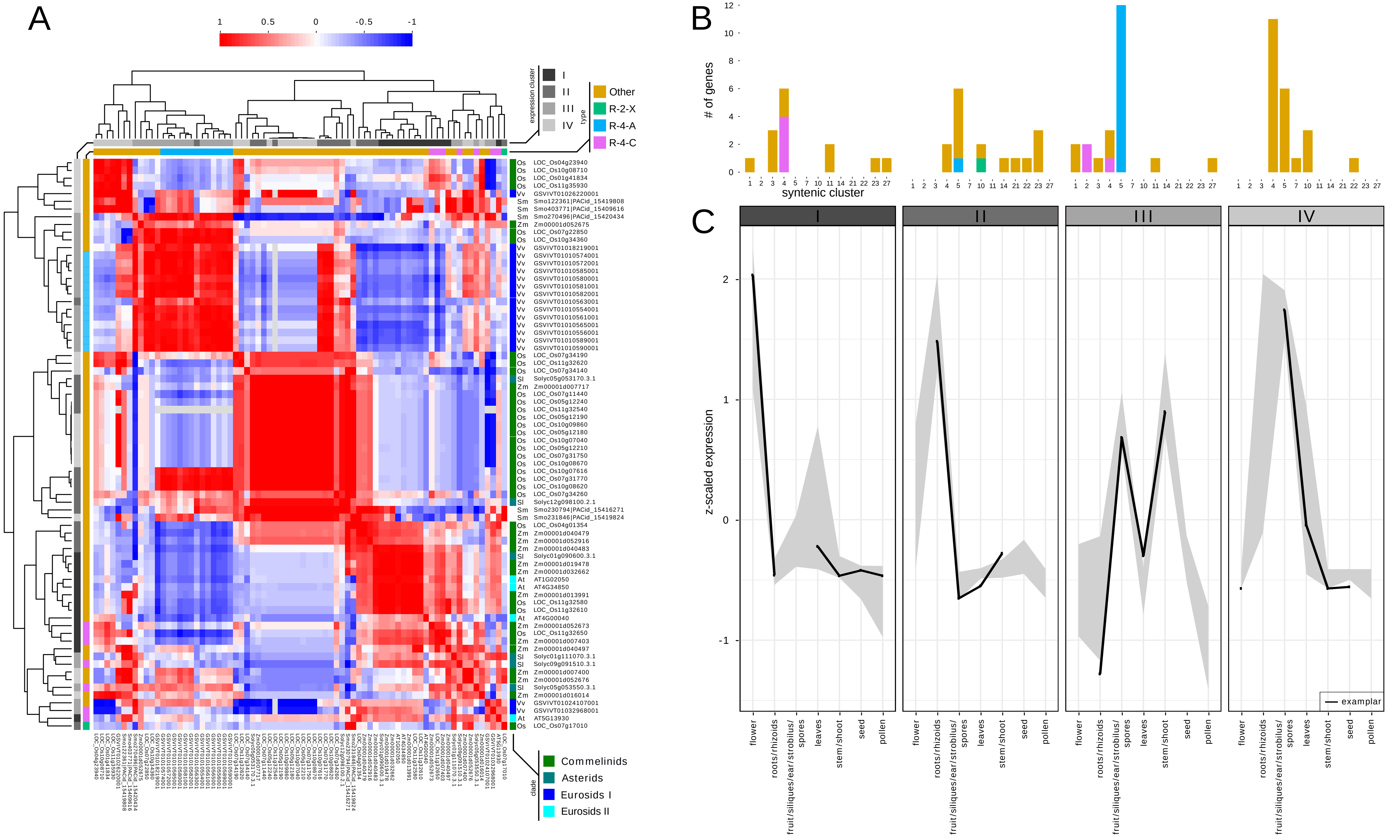
A
1
0.5
0
-0.5
-1
expression cluster
I
II
III
IV
type
Other
R-2-X
R-4-A
R-4-C
Os LOC_Os04g23940
Os LOC_Os10g08710
Os LOC_Os01g41834
Os LOC_Os11g35930
Vv GSVIVT01026220001
Sm Smo122361|PACid_15419808
Sm Smo403771|PACid_15409616
Sm Smo270496|PACid_15420434
Zm Zm00001d052675
Os LOC_Os07g22850
Os LOC_Os10g34360
Vv GSVIVT01018219001
Vv GSVIVT01010574001
Vv GSVIVT01010572001
Vv GSVIVT01010585001
Vv GSVIVT01010580001
Vv GSVIVT01010581001
Vv GSVIVT01010582001
Vv GSVIVT01010563001
Vv GSVIVT01010554001
Vv GSVIVT01010561001
Vv GSVIVT01010565001
Vv GSVIVT01010556001
Vv GSVIVT01010589001
Vv GSVIVT01010590001
Os LOC_Os07g34190
Os LOC_Os11g32620
Os LOC_Os07g34140
Sl Solyc05g053170.3.1
Zm Zm00001d007717
Os LOC_Os07g11440
Os LOC_Os05g12240
Os LOC_Os11g32540
Os LOC_Os05g12190
Os LOC_Os10g09860
Os LOC_Os05g12180
Os LOC_Os10g07040
Os LOC_Os05g12210
Os LOC_Os07g31750
Os LOC_Os10g08670
Os LOC_Os10g07616
Os LOC_Os07g31770
Os LOC_Os10g08620
Os LOC_Os07g34260
Sl Solyc12g098100.2.1
Sm Smo230794|PACid_15416271
Sm Smo231846|PACid_15419824
Os LOC_Os04g01354
Zm Zm00001d040479
Zm Zm00001d052916
Zm Zm00001d040483
Sl Solyc01g090600.3.1
Zm Zm00001d019478
Zm Zm00001d032662
At AT1G02050
At AT4G34850
Zm Zm00001d013991
Os LOC_Os11g32580
Os LOC_Os11g32610
At AT4G00040
Zm Zm00001d052673
Os LOC_Os11g32650
Zm Zm00001d007403
Zm Zm00001d040497
Sl Solyc01g111070.3.1
Sl Solyc09g091510.3.1
Zm Zm00001d007400
Zm Zm00001d052676
Sl Solyc05g053550.3.1
Zm Zm00001d016014
Vv GSVIVT01024107001
Vv GSVIVT01032968001
At AT5G13930
Os LOC_Os07g17010
clade
Commelinids
Asterids
Eurosids I
Eurosids II
B
# of genes
0
2
4
6
8
10
12
1 2 3 4 5 7 10 11 14 21 22 23 27
syntenic cluster
C
I
II
III
IV
z-scaled expression
2
1
0
-1
flower
roots/rhizoids
fruit/siliques/ear/strobilus/spores
leaves
stem/shoot
seed
pollen
exemplar
