# Supplementary material for: Kingdom-wide analysis of the evolution of the plant type III polyketide synthase superfamily
Source: Plant Physiol. 2020 Dec 30;185(3):857–75. doi: 10.1093/plphys/kiaa086 (PMC8133574; doi:10.1093/plphys/kiaa086)
Supplement: kiaa086_Supplementary_Data [file pp.01200.2020-s03.pdf › pp.01200.2020-s03.pdf]

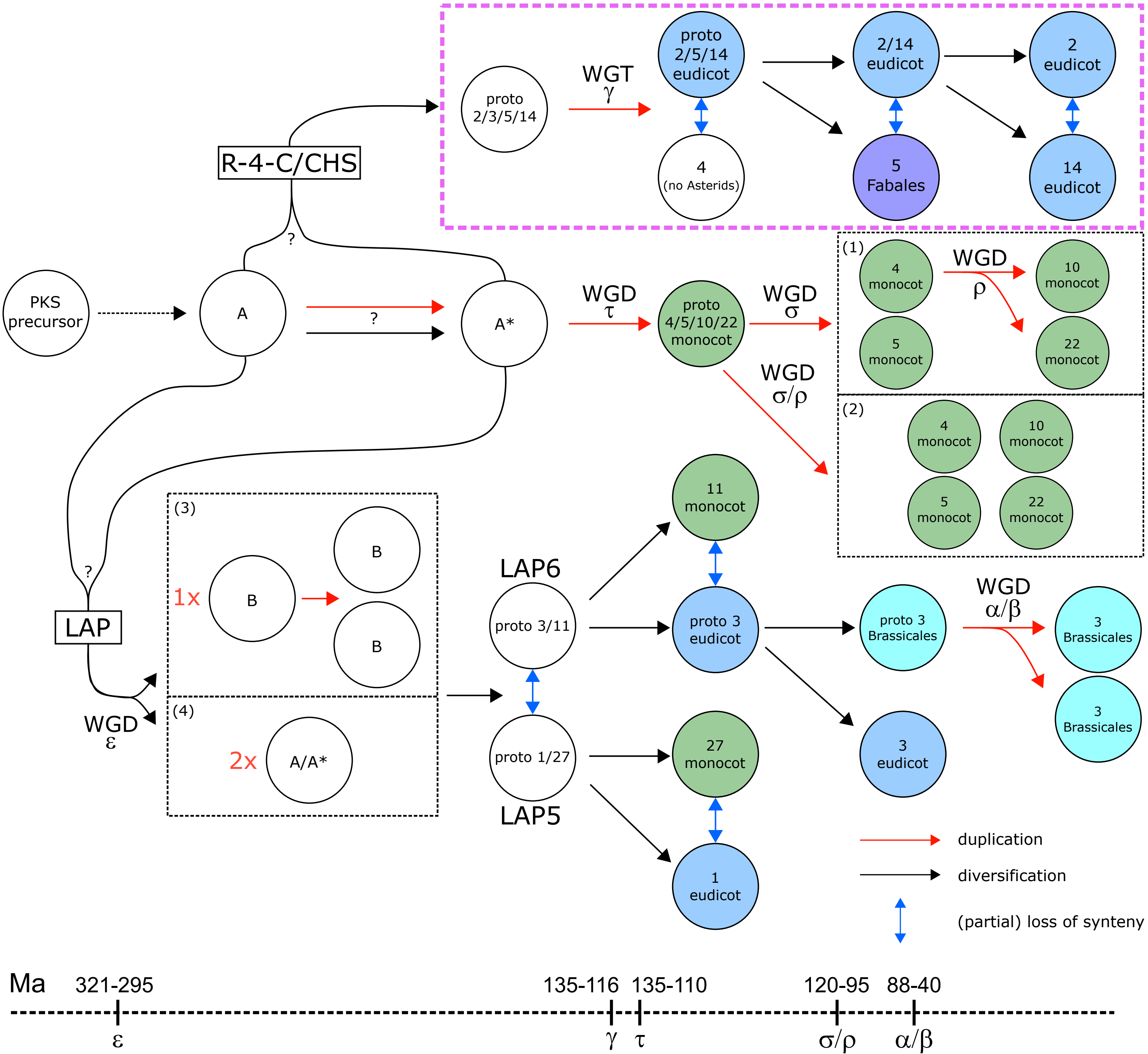

proto 2/3/5/14
WGT γ
proto 2/5/14 eudicot
4 (no Asterids)
2/14 eudicot
5 Fabales
2 eudicot
14 eudicot
R-4-C/CHS
?
PKS precursor
A
?
A*
WGD τ
proto 4/5/10/22 monocot
WGD σ
(1)
4 monocot
5 monocot
WGD ρ
10 monocot
22 monocot
WGD σ/ρ
(2)
4 monocot
10 monocot
5 monocot
22 monocot
?
LAP
WGD ε
(3)
1x
B
B
B
(4)
2x
A/A*
LAP6
proto 3/11
proto 1/27
LAP5
11 monocot
proto 3 eudicot
27 monocot
1 eudicot
proto 3 Brassicales
3 eudicot
WGD α/β
3 Brassicales
3 Brassicales
duplication
diversification
(partial) loss of synteny
Ma
321-295
135-116
135-110
120-95
88-40
ε
γ
τ
σ/ρ
α/β
